# A Mechanistic Investigation of the *N*-Hydroxyphthalimide Catalyzed Benzylic Oxidation Mediated by Sodium Chlorite

**DOI:** 10.1021/acs.joc.4c00583

**Published:** 2024-05-15

**Authors:** Thomas Grunshaw, Susanna H. Wood, Stephen Sproules, Andrew Parrott, Alison Nordon, Peter D. P. Shapland, Katherine M. P. Wheelhouse, Nicholas C. O. Tomkinson

**Affiliations:** † Department Pure and Applied Chemistry, Thomas Graham Building, University of Strathclyde, Glasgow G1 1XL, U.K.; ‡ School of Chemistry, University of Glasgow, Glasgow G12 8QQ, U.K.; § GlaxoSmithKline R&D, Gunnels Wood Road, Stevenage SG1 2NY, U.K.

## Abstract

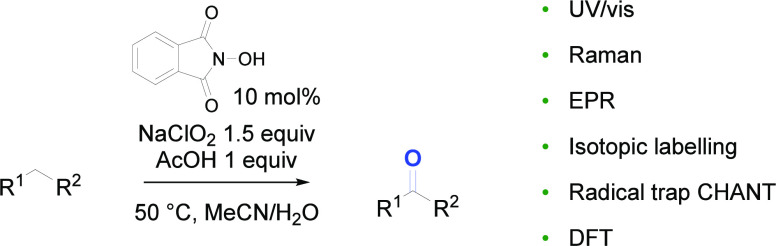

A detailed investigation into the mechanistic course of *N*-hydroxyphthalimide catalyzed oxidation of benzylic centers using sodium chlorite as the stoichiometric oxidant is reported. Through a combination of experimental, spectroscopic, and computational techniques, the transformation is interrogated, providing improved reaction conditions and an enhanced understanding of the mechanism. Performing the transformation in the presence of acetic acid or a pH 4.5 buffer leads to extended reaction times but improves the catalyst lifetime, leading to the complete consumption of the starting material. Chlorine dioxide is identified as the active oxidant that is able to oxidize the *N*-hydroxyphthalimide anion to the phthalimide-*N*-oxyl radical, the proposed catalytically active species, which is able to abstract a hydrogen atom from the substrate. A second molecule of chlorine dioxide reacts with the resultant radical and, after loss of hypochlorous acid, leads to the observed product. Through a broad variety of techniques including UV/vis, EPR and Raman spectroscopy, isotopic labeling, and the use of radical traps, evidence for the mechanism is presented that is supported through electronic structural calculations.

## Introduction

The development of green and sustainable processes represents a major driving force in the evolution of synthetic methodology. Guided by the Principles of Green Chemistry, significant inroads into achieving these goals in bond construction procedures have been made.^1−3^ Oxidation is a fundamental class of transformation within synthesis that is central to both industrial processes and laboratory research.^4−10^ To address sustainability by avoiding the use of transition metals, substantial efforts have been made to develop organocatalytic oxidation procedures, specifically those that use oxygen or peroxide as the terminal oxidant, with particular success being achieved with TEMPO and its derivatives.^11−14^ Despite delivering a solution to this important problem, when performed using organic solvents, oxygen introduces a significant risk of fire,^15^ whereas organic peroxides are often explosive^16^ and where possible should be avoided, highlighting a need for alternative stoichiometric oxidants and detailed understanding of their behavior.

Since the first report by Cohn in 1880,^17^*N*-hydroxyphthalimide (NHPI) **1** has been developed as a robust and versatile organocatalyst for a broad variety of C–C, C–O, C–N, and C–S bond forming reactions.^18−21^ Of specific note in the chemistry of this molecule is the conversion of **1** to the phthalimide-*N*-oxyl (PINO) radical **2**, a compound that is stable enough to function as a catalytically active species within many transformations. Abstraction of a hydrogen atom from organic substrates by **2** regenerates NHPI **1** and forms a reactive carbon centered radical that can participate in clean and efficient bond forming processes.^22−31^

**Scheme 1:**
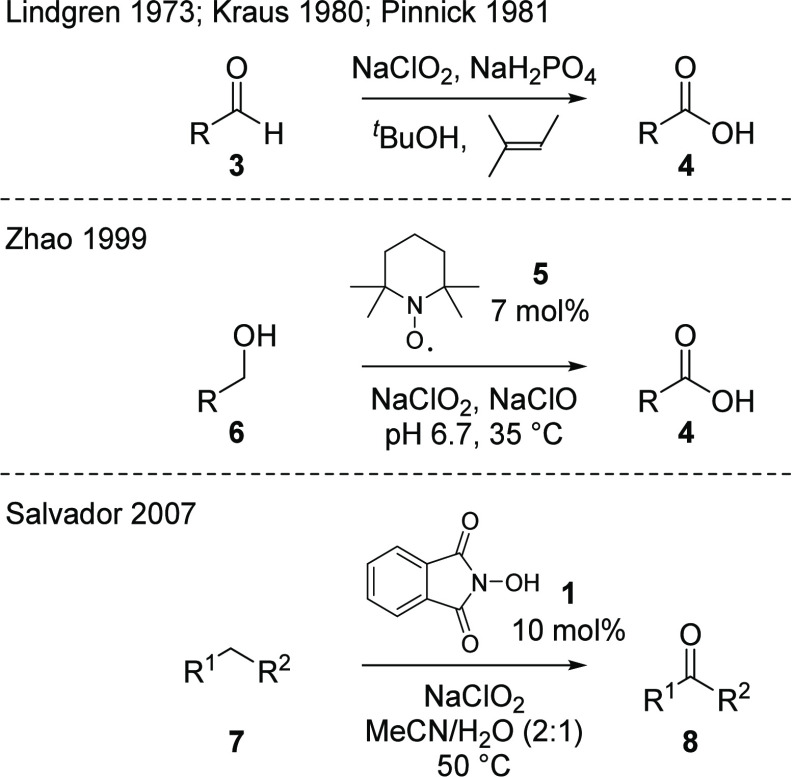
Oxidation of Aldehydes, Primary Alcohols, and Benzylic Centers Using Sodium Chlorite

Sodium chlorite is a cheap, readily available inorganic reagent used industrially for the bleaching of textiles and wood pulp and is commonly used as a disinfectant.^32,33^ In these processes, chlorite is oxidized to chlorine dioxide, which is the active oxidizing agent.^34^ Sodium chlorite has also been used as a stoichiometric oxidant in synthesis.^35,36^ From a mechanistic perspective, these transformations are diverse, highlighting the versatility and numerous pathways accessible with this convenient reagent. It is most commonly employed in the Lindgren oxidation of aldehydes **3** to carboxylic acids **4**,^37^ which was enhanced by Kraus and Roth^38^ and Kraus and Taschner^39^ and further exemplified by Pinnick et al. (Scheme 1).^40^ The analogous oxidation of imines to amides has also been demonstrated using equivalent conditions.^41^ By combining sodium chlorite with catalytic amounts of sodium hypochlorite and TEMPO **5**, the process can be extended to the direct oxidation of primary alcohols **6** to their corresponding carboxylic acids **4**, further illustrating the applicability of this reagent.^42^ A significant challenge in the use of sodium chlorite is controlling the complex equilibria exhibited by chloroxy species, where the formation of reactive chlorine moieties can be detrimental to the methodology. Several strategies have emerged to address this problem including the control of pH,^42^ use of sacrificial reagents,^38^ and judicious choice of solvent,^43^ which suggest that further exploitation of this oxidant in synthesis is possible.

**Figure 1:**
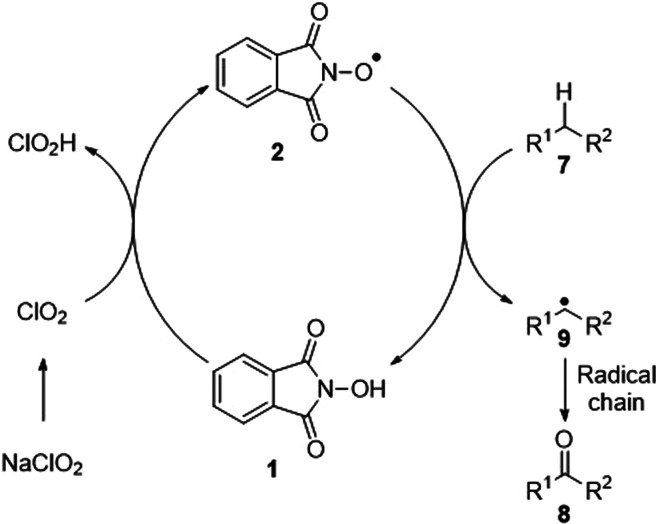
Salvador mechanistic hypothesis.^44^

In 2007, Silvestre and Salvador reported a simple and effective method for allylic and benzylic oxidation reactions using sodium chlorite as the stoichiometric oxidant.^44^ Treatment of the substrate **7** with 1.5 equiv of sodium chlorite and NHPI **1** (10 mol %) at 50 °C in a mixture of acetonitrile and water (2:1) for 1 h gave the corresponding ketone **8** (Scheme 1). Overall, this catalytic oxidation represents a green and efficient process with benign coproducts of sodium chloride and water. Within their report, it was proposed that the oxidation was a free-radical process involving formation of chlorine dioxide *in situ* that abstracted a hydrogen atom from NHPI **1** leading to the formation of PINO radical intermediate **2** (Figure 1). This reactive intermediate then removed a hydrogen atom from a benzylic or allylic center with the resulting stabilized radical **9** being oxidized in a radical chain mechanism^45,46^ to give the corresponding ketone **8**. Despite the significant potential of this reaction, its application in synthesis has curiously been limited.^47,48^ We believe that a greater understanding of the mechanistic course of the transformation would enhance its uptake and application by the synthetic community. Within this paper, we report further development of the reaction and provide experimental, spectroscopic, and theoretical insight into this complex, intriguing, and useful process.

## Results and Discussion

**Scheme 2:**
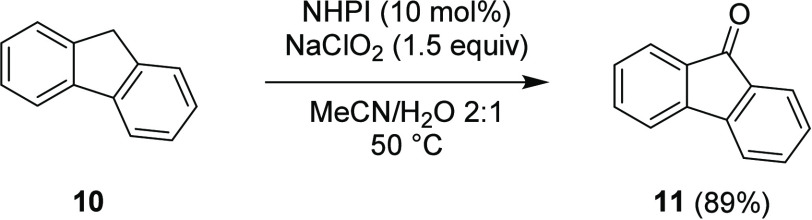
Repeat of the Salvador Work^44^

The investigation began by repeating the work of Salvador using fluorene **10** as a model substrate to allow direct comparison with the literature precedent (Scheme 2).^44^ Optimal consistency in results was found through a slightly modified protocol that involved treatment of an acetonitrile solution of fluorene **10** and NHPI **1** (10 mol %) at 50 °C with an aqueous solution of sodium chlorite (80%, 1.5 equiv) over 15 min. This resulted in a 95% conversion of the fluorene starting material **10** and an 89% isolated yield of the oxidized product **11**, in agreement with the results reported by Salvador (NaClO_2_ 1.5 equiv, NHPI **1** 10 mol %, MeCN/H_2_O (3:1), 50 °C, 1 h, 90% yield).

Of note, in this oxidation procedure, two color changes were observed. During the addition of sodium chlorite, the reaction mixture became orange. This changed to yellow soon after the addition of the oxidant was complete. The orange color was attributed to the NHPI catalyst on the basis of its appearance in basic aqueous solutions. The yellow color was attributed to a combination of the yellow fluorenone product **11** and chlorine dioxide, a proposed reactive species in Salvador’s report. The presence of chlorine dioxide within the experiment was later confirmed by a series of spectroscopic measurements (*vide infra*).

**Scheme 3:**
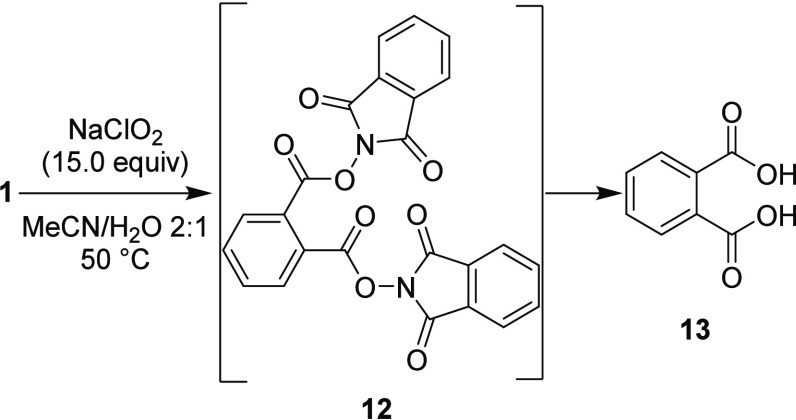
Reaction of NHPI **1** with Sodium Chlorite to Give Phthalic Acid **13**

Despite the reaction occurring upon the addition of sodium chlorite, complete conversion of the fluorene substrate **10** was not observed. Curiously, extended reaction times did not lead to increased conversion, with the reaction stalling within 10 min of chlorite addition. Further charges of sodium chlorite did not lead to increased conversion, whereas the addition of more NHPI (10 mol %) led to the consumption of the starting material. Based on these results, the stalling of the reaction was proposed to be through deactivation of the NHPI **1** catalyst. To investigate this further, an aqueous solution of sodium chlorite (15 equiv) was added to a solution of **1** in acetonitrile over 15 min. During the addition, an intense orange-red color was observed, which faded to yellow upon complete addition. Monitoring this process by LCMS showed consumption of the NHPI **1** and a peak corresponding to the trimeric species **12**, which decayed to phthalic acid **13** over time (Scheme 3). The trimerization of NHPI **1** to give **12** and its decomposition to phthalic acid **13** under basic conditions have been observed when investigating the kinetic reactivity of the PINO radical **2**.^49^ Mechanisms have been proposed for this process^50^ including a recent insightful investigation by Pratt et al. combining experimental and computational studies.^51^

Key to the literature findings was that the trimerization to **12** occurred under basic reaction conditions,^51^ and we speculated that a lower pH could increase the lifetime of the NHPI catalyst **1**. To test this hypothesis, the oxidation of fluorene **10** by sodium chlorite (1.5 equiv) and NHPI **1** (10 mol %) was performed in the presence of acetic acid (1.0 equiv). Although this reaction was slower than the transformation in the absence of acetic acid, it reached 97% conversion after 90 min and full conversion when left to stir overnight, the product **11** being isolated in 98% yield.

To probe this potential effect of pH, we ran a series of time course reactions using aqueous buffer solutions to replace the water. Buffers at pH 4, 7, and 9 were selected. Sodium chlorite (1.5 equiv) was dissolved in the appropriate buffer, and the solutions were added as single charges to an acetonitrile solution of fluorene **10** and NHPI **1** (10 mol %) maintained at 50 °C. The reactions were sampled at known times and were directly quenched into a 0.2 M solution of sodium metabisulfite in 2:1 THF/H_2_O. Quenched samples were analyzed by LCMS to establish a qualitative picture of the transformation (see the Supporting Information for copies of the time course traces).

From these reactions, several conclusions were drawn. First, the rate of conversion of fluorene **10** to fluorenone **11** was related to pH, with faster reactions being achieved at higher pH. Second, the rate of degradation of NHPI increased at higher pH. Third, in reactions conducted at higher pH, NHPI was consumed quicker than fluorene **10**, and when NHPI was fully consumed, the conversion of fluorene stopped, resulting in an incomplete transformation. Fourth, at lower pH, fluorene **10** was consumed quicker than NHPI **1**, allowing for full conversion of the starting material. Therefore, despite the slower rate of reaction, acidified reaction media provided a superior reaction outcome. We therefore conducted subsequent transformations in the presence of acetic acid (1.0 equiv) that led to a slower reaction rate but a longer catalyst lifetime leading to higher yields and improved reproducibility.

**Figure 2:**
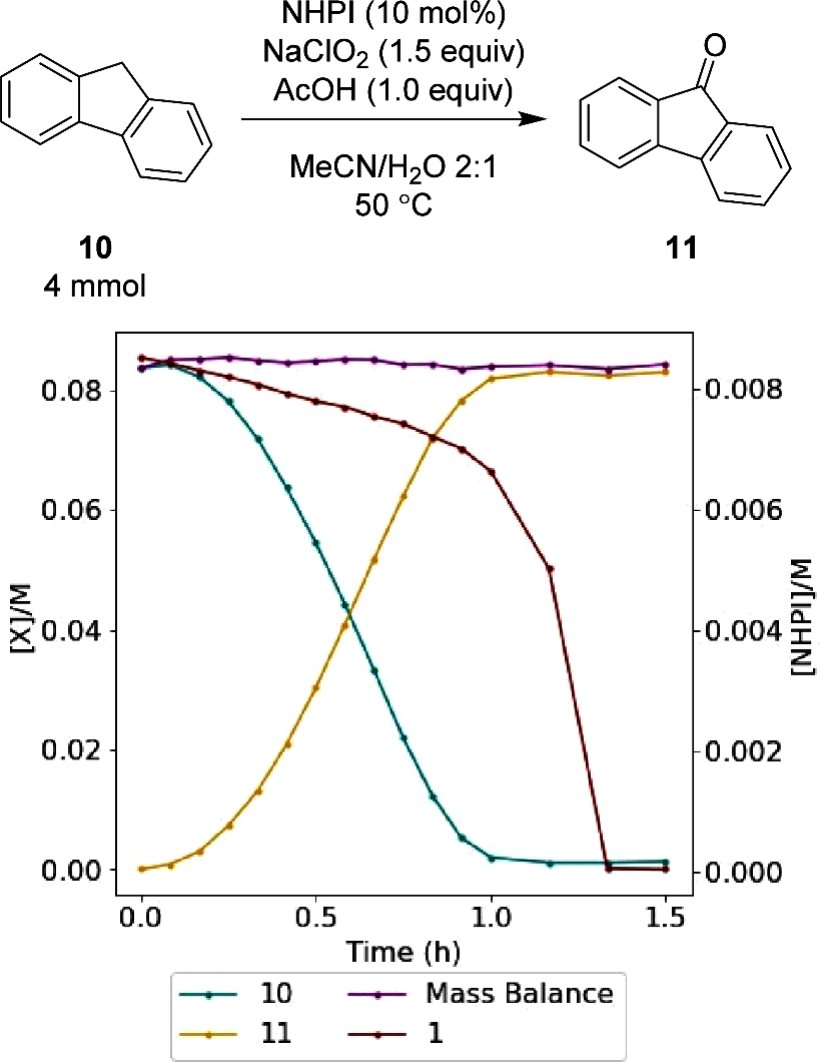
Monitoring the oxidation of fluorene **10** to fluorenone **11** by HPLC using an EasyMax 102 Advanced Thermostat system.

To enable reaction monitoring with accurate quantification, calibration curves were prepared for four species—fluorene **10**, fluorenone **11**, NHPI **1**, and phthalic acid **13**—using 1,4-dicyanobenzene as an internal standard (see the Supporting Information for full details). The reaction was performed in an EasyMax 102 Advanced Thermostat system, manually sampling every 5 min over the first hour and then every 10 min until 90 min, at which point heating and stirring were stopped (Figure 2). The reaction profile revealed a number of insights. The mass balance of fluorene **10** to fluorenone **11** was constant across the full profile, with no discernible intermediates observed by HPLC or implied by the mass balance. There was a significant induction period observed, with a steady increase in the reaction rate that reached a maximum after 30 min. In addition, the degradation of NHPI **1** was observed to be steady during the reaction, with 25% consumed during the conversion of **10** to **11**. Interestingly, the rate of degradation increased rapidly once fluorene **10** was consumed, with NHPI **1** fully consumed after 80 min to unidentified byproducts. The reaction showed good reproducibility, with replicates at 500 rpm stirring rate showing good fidelity. The mass transfer effects of the reaction were investigated with 150 rpm stirring, showing no clear difference in the reaction profile from the 500 rpm profile (see the Supporting Information for full details).

**Scheme 4:**
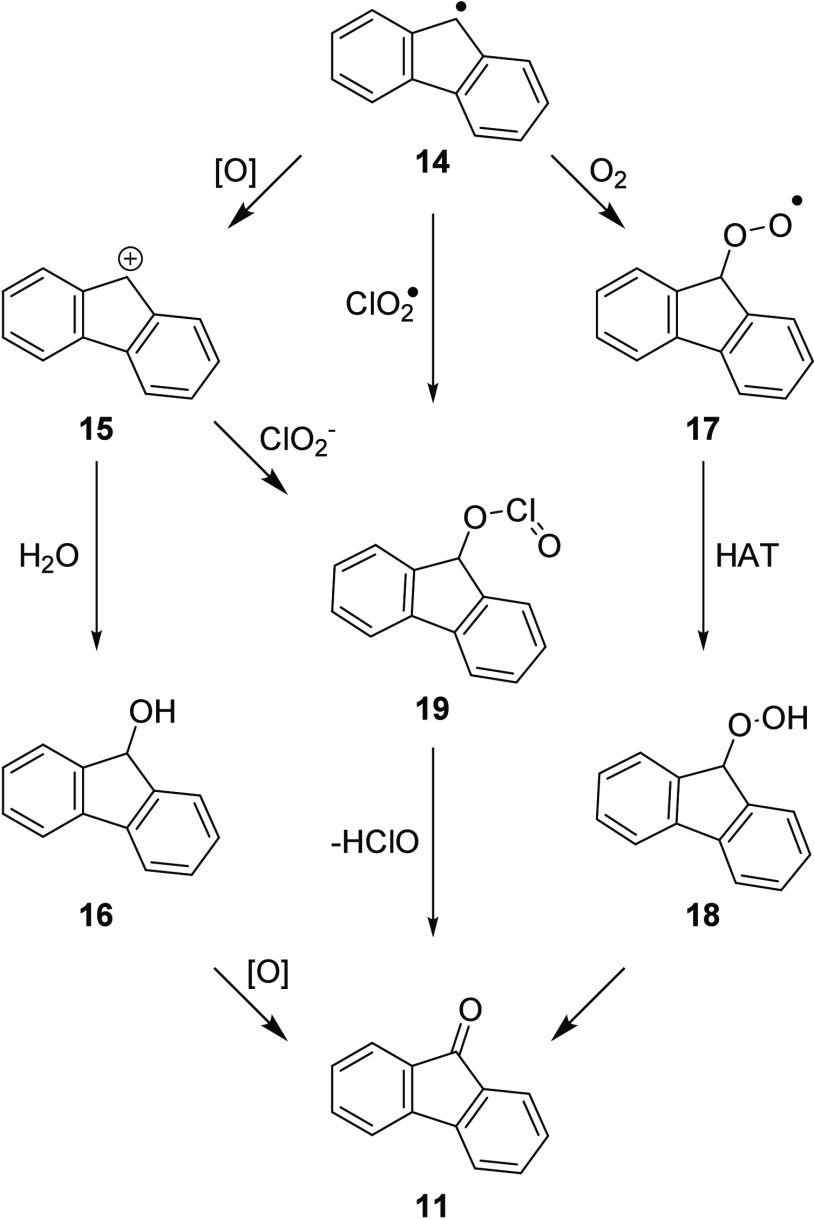
Potential Pathways for the Oxygenation of Radical Intermediate **14**

Having established a method to monitor the transformation, three potential pathways were considered for the formation of fluorenone **11** from fluorenyl radical **14**, each of which had a unique source of the carbonyl oxygen (Scheme 4): an alcohol pathway that sourced the carbonyl oxygen atom from water, an aerobic pathway where the carbonyl oxygen would originate from dioxygen, and a chlorite pathway where the oxygen source would be chlorine dioxide. A series of experiments were conducted to probe each of these potential pathways.

Exposure of the alcohol **16** to standard reaction conditions (NaClO_2_ 1.5 equiv, NHPI **1** 10 mol %, AcOH 1.0 equiv, MeCN/H_2_O (2:1), 50 °C) showed some conversion to fluorenone **11**; however, the reaction was considerably slower. Conversion of fluorenol **16** reached just 14% after 90 min and 94% after 24 h. In addition, the oxidation of fluorene **10** in the presence of ^18^OH_2_ (10% v/v) led to ketone **11** (98%) with no isotope incorporation. To support this observation, stirring a sample of fluorenone **11** with acetic acid (1.0 equiv) in 2:1 MeCN/^18^OH_2_ for 2 h at 50 °C showed no incorporation of the heavy atom by LCMS analysis. Based upon these combined experiments, the alcohol pathway was excluded from our investigations.

To probe the aerobic pathway, the reaction of **10** with sodium chlorite (1.0 equiv) in the presence of NHPI **1** (10 mol %) and acetic acid (1.0 equiv) (MeCN/H_2_O (2:1), 50 °C, 1 h) was conducted under an aerobic (79.5 ± 0.3 yield) and a nitrogen (76.7 ± 0.7 yield) atmosphere. Despite a slightly improved conversion under an aerobic atmosphere, the similar conversions under nitrogen and air indicated that dioxygen was not the primary source of the carbonyl oxygen atom; however, it could represent a minor pathway based on the marginally higher conversions observed.

Our attention moved to chlorine dioxide as the oxygen source through a radical–radical combination–elimination process. If the reaction proceeded through the elimination of hypochlorous acid via **19**, the oxygen atom incorporated within the product would arise from the sodium chlorite reagent.

Following a literature procedure for the generation of NaCl^18^O_2_,^52^^18^O-labeled NaClO_3_ was generated from NaClO_3_ and ^18^OH_2_ in one chamber of a COware^53^ apparatus (see the Supporting Information for the experimental setup). The labeled chlorate was then converted to chlorine dioxide gas by the addition of sodium sulfite,^52^ which flowed into the second chamber containing a DMSO solution of fluorene **10**, NHPI **1**, and acetic acid. GCMS of the crude reaction mixture showed the presence of fluorenone **11** containing an isotopically labeled carbonyl oxygen in 45% relative abundance (compared with 1% for the equivalent unlabeled experiment).

Having determined that chlorine dioxide provided the source of oxygen, we also examined the potential role of hypochlorite as the oxidizing agent. Use of sodium hypochlorite (3.0 equiv) as the stoichiometric oxidant in place of sodium chlorite led to a complex mixture of products. Importantly, fluorenone **11** was not observed within this reaction mixture, indicating that sodium hypochlorite alone was not a competent oxidant for this transformation.

DMSO is known to selectively quench hypochlorite and chlorine without reacting with higher chlorine oxides including chlorine dioxide and chlorite.^43^ To probe the role of hypochlorous acid, DMSO (2.0 equiv) was added to a standard reaction (**10**, NaClO_2_ 1.5 equiv, NHPI **1** 10 mol %, AcOH 1.0 equiv in MeCN/H_2_O (2:1), 50 °C), leading to a suppressed process with just 17% conversion after 2 h. Increasing the charge of DMSO to 10.0 equiv further inhibited the reaction rate, resulting in less than 10% conversion to fluorenone **11** after 2 h. Although DMSO did not completely inhibit the reaction, the suppression in the rate suggested that the hypochlorous acid was being quenched by DMSO.

**Scheme 5:**
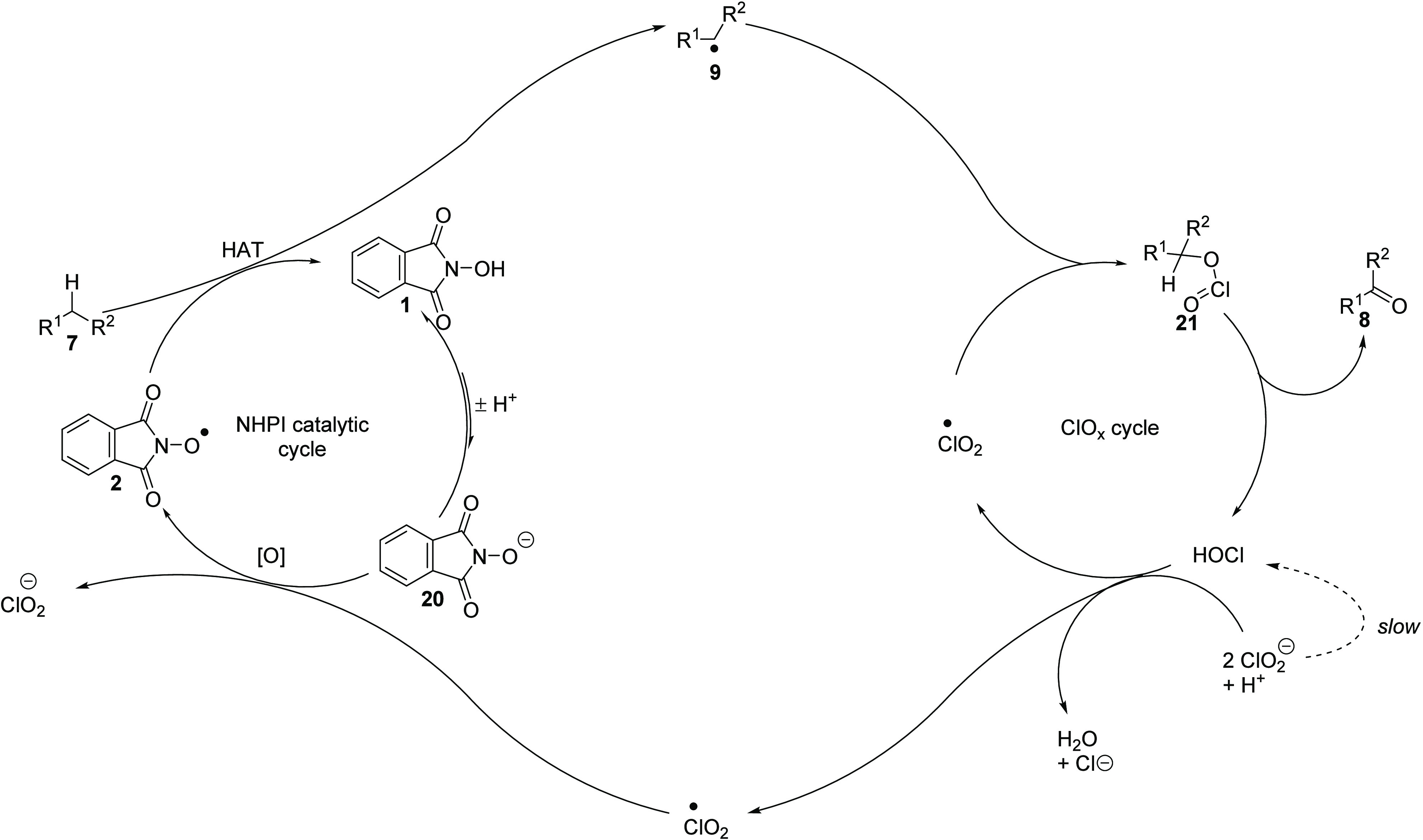
Proposed Catalytic Cycle

These studies allowed us to propose an overall mechanism to explain a number of observations (Scheme 5). Disproportionation of chlorite under acidic conditions generates hypochlorous acid.^54^ This process is slow but continuous and generates hypochlorous acid throughout the reaction. Hypochlorous acid can react with 2 equiv of chlorite and a proton to give 2 equiv of chlorine dioxide plus water and a chloride anion.^55,56^ The generation of additional hypochlorous acid from the oxidation would further accelerate the formation of chlorine dioxide, accounting for the induction period observed.

Once formed, we propose that chlorine dioxide has two roles in the reaction. The first is to oxidize PINO anion **20**, which is in equilibrium with NHPI **1** in the solution. The resulting PINO radical **2** then reacts with the organic substrate **7** via hydrogen atom transfer to provide the organic radical **9**, regenerating NHPI **1** and completing the NHPI catalytic cycle. The second function of chlorine dioxide is to undergo radical–radical coupling with **9**, resulting in chlorous acid ester **21**. This undergoes elimination or pericyclic fragmentation to yield ketone **8** and hypochlorous acid, which enables the formation of more chlorine dioxide, continuing the cycle.

NHPI **1** is a weak acid, with a p*K*_a_ of 6.3^57^; therefore, in solution, it is in equilibrium with its anionic form **20**. As the pH increases, the proportion of the anion in solution increases. It follows that the anionic form **20** is the species that is oxidized in solution, which would justify the higher rates of reaction at higher pH. This is consistent with the observations of Constentin, who found a higher oxidation potential of NHPI below pH 6.3 (i.e., the p*K*_a_), indicative of proton transfer followed by SET.^58^ This oxidation potential decreased with increasing anion concentration.

**Figure 3:**
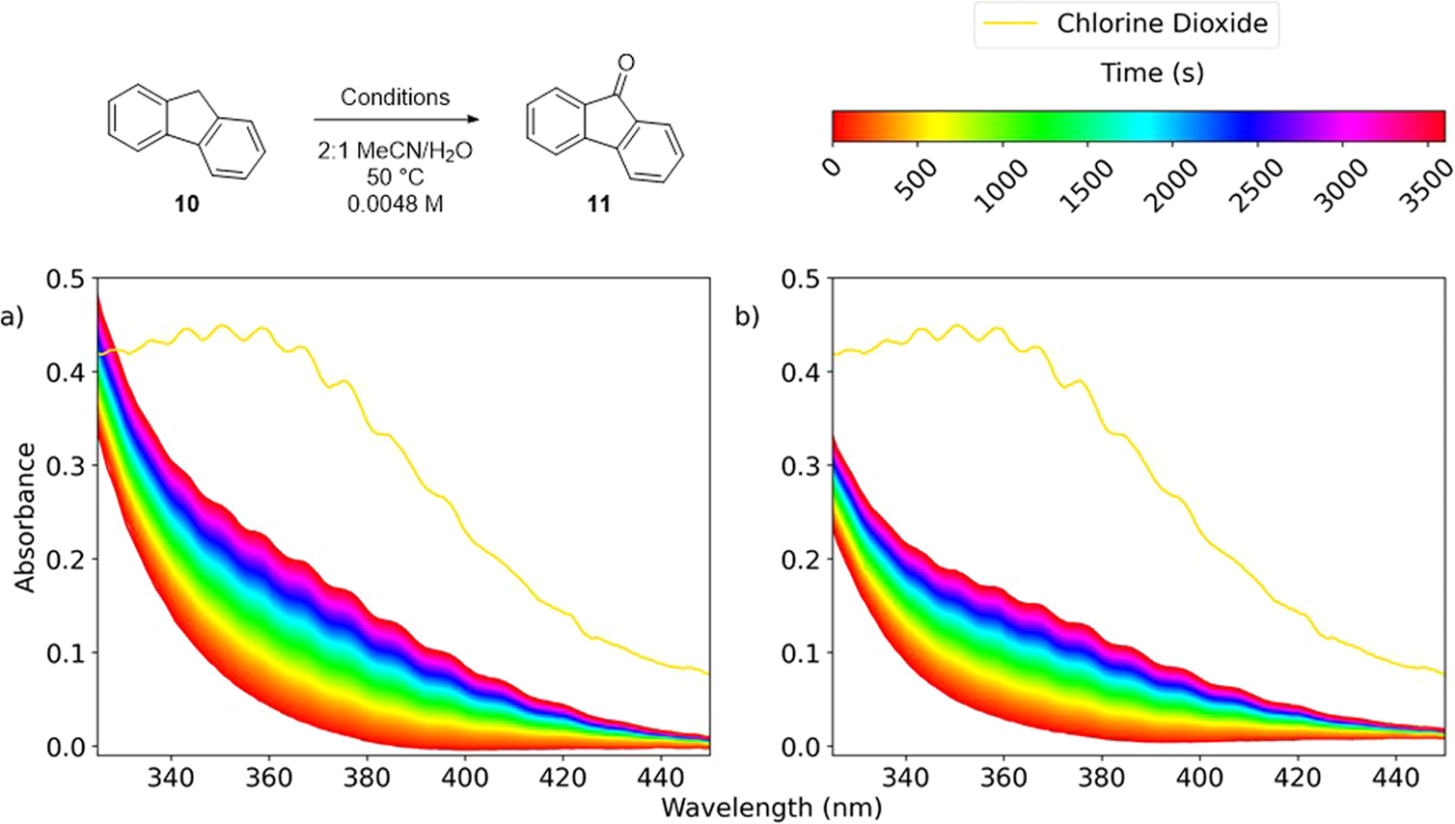
UV/vis spectroscopy experiments. (a) Fluorene **10**, NaClO_2_ 1.5 equiv, NHPI **1** 10 mol %, acetic acid 1.0 equiv, with trace of 4.37 × 10^–4^ M ClO_2_ solution overlaid. (b) Fluorene **10**, NaClO_2_ 1.5 equiv, acetic acid 1.0 equiv, with trace of 4.37 × 10^–4^ M ClO_2_ overlaid.

We sought to gain further experimental evidence to support this mechanistic hypothesis. The oxidation of fluorene **10** was monitored by UV spectroscopy (Figure 3). The UV spectrum of a 4.37 × 10^–4^ M aqueous solution of chlorine dioxide is overlaid on each spectrum in Figure 3 (yellow trace), which displays a characteristic peak at λ_max_ 360 nm with vibronic coupling evident in the spectrum.^59^ Conducting a reaction at 4.80 × 10^–3^ M concentration of **10** confirmed the formation of chlorine dioxide (Figure 3a), which had been initially proposed because of the intense yellow coloration of the reaction mixture. When the NHPI catalyst was omitted from the reaction mixture, the formation of chlorine dioxide was detected by UV/vis spectroscopy, indicating that this substance was also present in any uncatalyzed reaction pathway albeit at significantly lower concentrations (Figure 3b).

**Figure 4:**
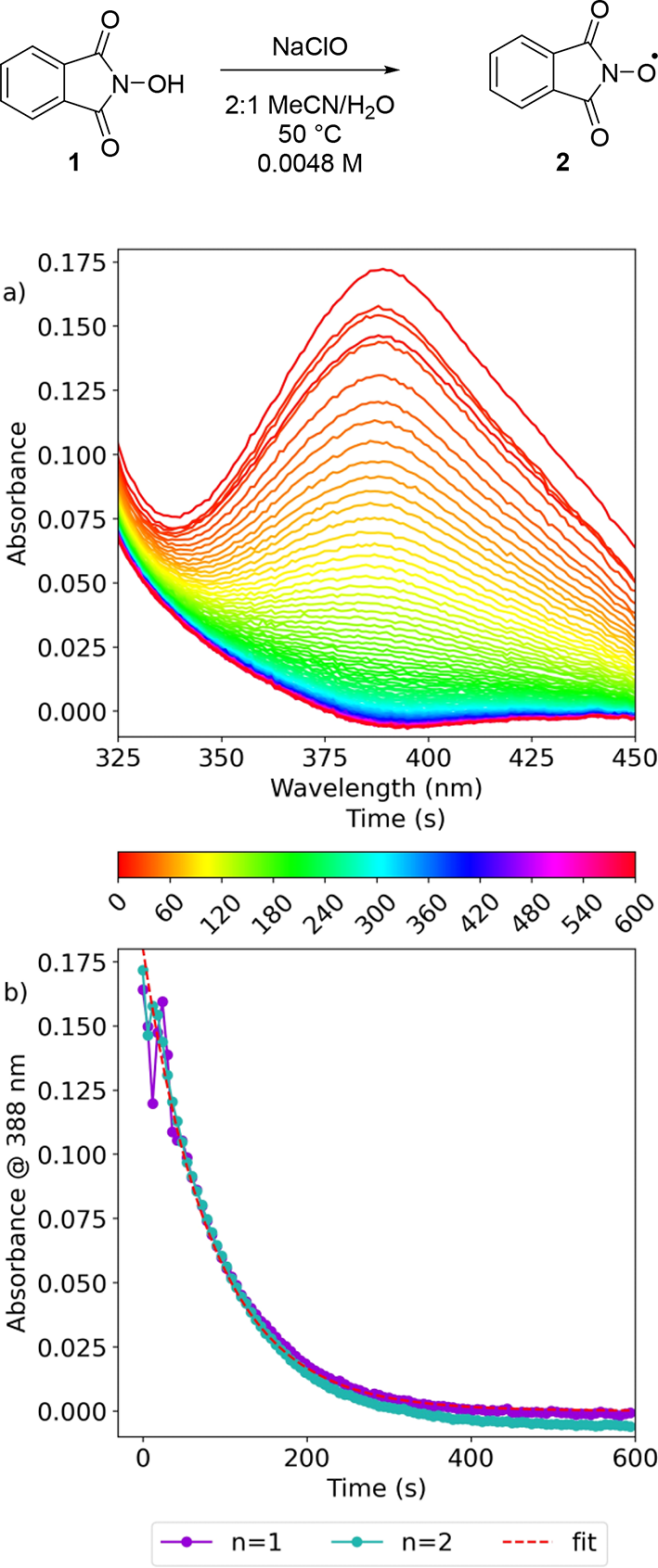
Reaction of NHPI **1** with NaClO. (a) Overlaid spectra of reaction between NHPI **1** and NaClO. (b) Absorbance at 388 nm showing the decay of PINO radical **2** (*n* = 2, *k*_1_ = 1.17 × 10^–2^ s^–1^, *k*_2_ = 1.16 × 10^–2^ s^–1^).

The PINO radical **2** has been observed previously by UV/vis spectroscopy (λ_max_ 388 nm).^60^ However, because of the broad and intense signal of chlorine dioxide at a similar wavelength (λ_max_ = 360 nm), it could not be observed under the standard reaction conditions. When sodium hypochlorite was added to a MeCN/H_2_O (2:1) solution of NHPI **1**, a signal corresponding to **2** formed that decayed over time (Figure 4). This provided evidence that it was possible for **2** to form in the reaction mixture through the oxidation of the PINO anion **20** by hypochlorite, but evidence that chlorine dioxide could facilitate this oxidation remained elusive at this stage.

**Figure 5:**
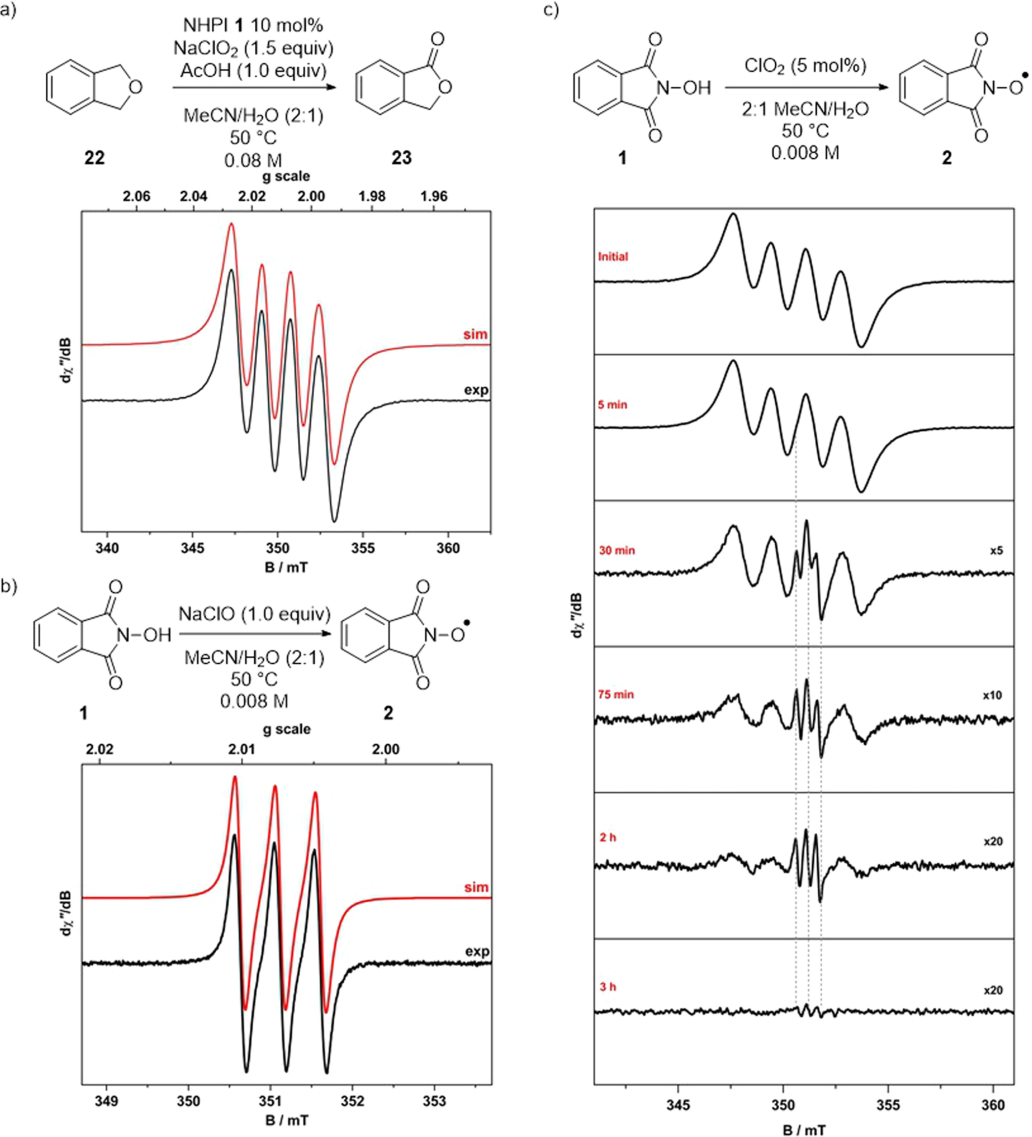
X-band EPR spectra recorded in MeCN/H_2_O (2:1) solution at ambient temperature (experimental conditions: frequency, 9.8647 GHz; power, 0.63 mW; modulation, 0.05 mT; experimental data represented by the black line; simulations depicted by the red trace): (a) chlorine dioxide generated under standard reaction conditions (*g*_iso_ = 2.0095; *A*_iso_{^35,37^Cl} = 16.5 × 10^–4^ cm^–1^). (b) PINO radical **2** by treatment of NHPI **1** with NaClO (*g*_iso_ = 2.00734; *A*_iso_{^14^N} = 4.6 × 10^–4^ cm^–1^). (c) Evolution of PINO radical **2** by treatment of excess NHPI **1** with chlorine dioxide. Spectra were enlarged to improve visibility. Drop lines indicate the emergence of the PINO radical with a concomitant decrease in the four-line signal for the chlorine dioxide radical.

To collect further evidence for the presence of radical species within the reaction mixture, EPR experiments were performed. Using phthalan **22** as the substrate under standard reaction conditions (NaClO_2_ 1.5 equiv, NHPI **1** 10 mol %, AcOH 1.0 equiv in MeCN/H_2_O (2:1)), a signal characteristic of the chlorine dioxide radical was observed to be generated (Figure 5a). Once again, the PINO radical **2** could not be observed under these conditions, likely because of the low concentrations generated in solution and its rapid consumption during the transformation. The PINO radical **2** was observed by mixing NHPI with a solution of sodium hypochlorite,^61^ which produced a signal consistent with the presence of an *N*-oxyl radical (Figure 5b). Further experiments were required to determine whether **2** could be produced via the reaction of NHPI **1** with chlorine dioxide. A standard 4.0 × 10^–4^ M solution of chlorine dioxide was generated in MeCN/water (2:1), and an NHPI **1** solution 20 times the concentration of the chlorine dioxide solution was added. The chlorine dioxide signal reduced in intensity after the addition of an aliquot of the NHPI **1** solution. Over time, the signal corresponding to PINO radical **2** began to appear as the chlorine dioxide signal diminished (Figure 5c). This is consistent with the consumption of chlorine dioxide in the oxidation of NHPI **1** and provides clear evidence that this is possible under the reaction conditions. Furthermore, when a solution of pregenerated PINO anion **20** (as the potassium salt) (MeCN/H_2_O (2:1)) was added to a chlorine dioxide solution (4.0 × 10^–4^ M), the generation of the PINO radical **2** was much faster, suggesting that the anion **20** is oxidized to the radical **2** more readily than NHPI **1** (see the Supporting Information for full details).

**Figure 6:**
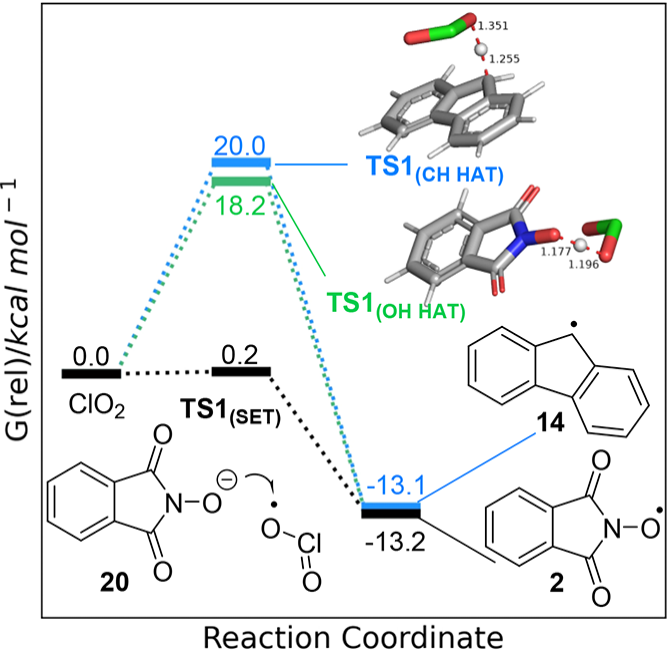
Electronic structure calculations (UM06-2X-D3/6-311++G(d,p) C-PCM acetonitrile). Potential pathways for the reaction of chlorine dioxide with the PINO anion **20** (**TS1**_**(SET)**_) (black), abstraction of a hydrogen atom from NHPI **1** (**TS1**_**(OH HAT)**_) (green), and abstraction of a hydrogen atom from **10** (**TS1**_**(CH HAT)**_) (blue).

Electronic structure calculations were carried out to develop a computational model to support the proposed mechanism. The UM06-2X functional was selected along with the 6-311++G(d,p) with Grimme’s empirical dispersion model D3 to account for intramolecular interactions as a relatively inexpensive methodology that provided acceptable performance when computing barrier heights.^62,63^ Solvation was accounted for using the C-PCM solvation model^64,65^ and built-in parameters for acetonitrile. Ground and transition state geometries were optimized, identifying stationary points corresponding to the starting materials and intermediates along with hydrogen atom transfer from the substrate to PINO radical **2** and the collapse of chlorine dioxide-substrate adduct **19**. Frequency calculations on the transition state structures yielded single imaginary frequencies consistent with the bond(s) being broken or formed whereas the starting material and intermediate computations were free of imaginary frequencies. The single electron oxidation of the PINO anion **20** to form PINO radical **2** and a chlorite anion was predicted to have a low barrier of 0.2 kcal mol^–1^**TS1**_(SET)_ (see the Supporting Information for details). In contrast, HAT from NHPI **1** (**TS1**_(OH HAT)_, Figure 6) was predicted to be more difficult, at 18.2 kcal mol^–1^. Direct HAT from fluorene **10** to chlorine dioxide (uncatalyzed pathway, **TS1**_(CH HAT)_) had the highest predicted barrier of 20.0 kcal mol^–1^.

**Figure 7:**
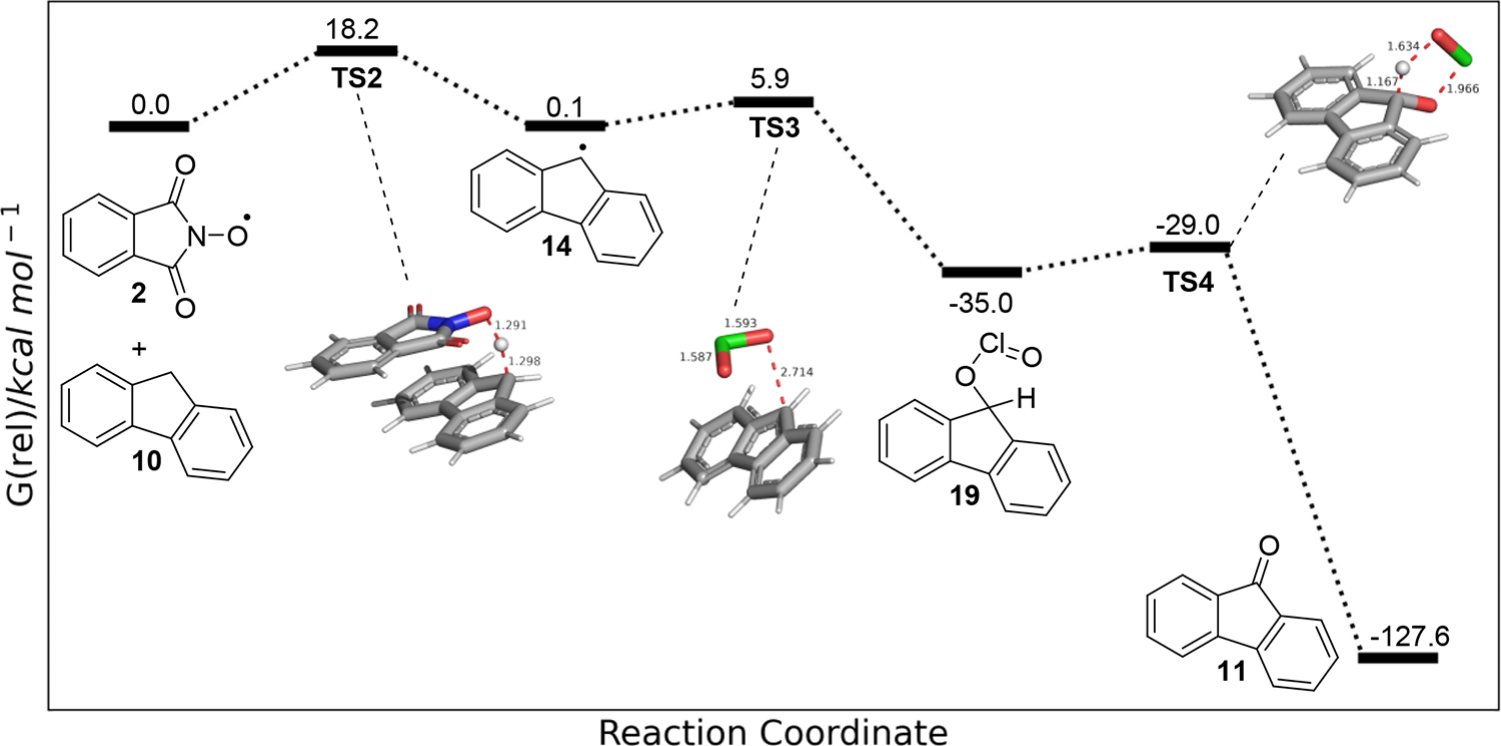
Calculated barriers for the proposed mechanism for the oxidation of **10** to **11** (UM06-2X-D3/6-311++G(d,p) C-PCM acetonitrile).

Hydrogen atom transfer from **10** to PINO radical **2** was calculated (**TS2**, Figure 7) to have the highest transition state barrier in the transformation, 18.2 kcal mol^–1^ higher in energy than **2** and **10**, forming the benzylic radical species **14** and NHPI **1**, 0.1 kcal mol^–1^ higher in energy than the starting materials. Subsequent radical–radical coupling (**TS3**) had a barrier of 5.9 kcal mol^–1^, resulting in the formation of intermediate **19**, which is 35 kcal mol^–1^ lower in energy than the reagents. An accessible transition state for the collapse of **19** to **11** via a pericyclic process (**TS4**) was found; however, an elimination pathway may also be feasible.

**Scheme 6:**
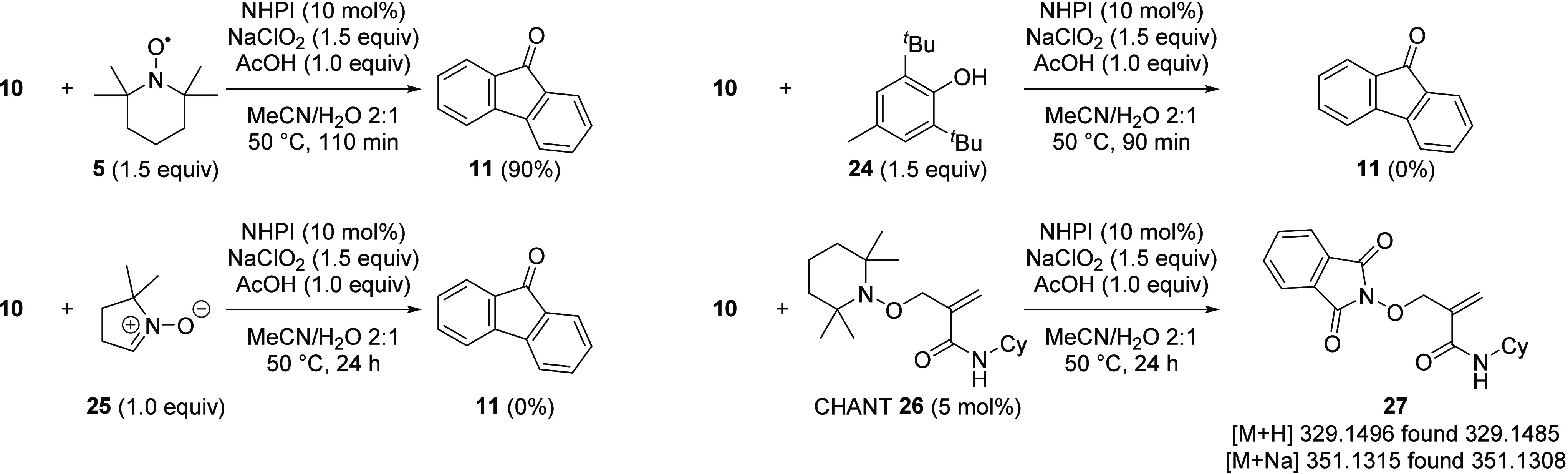
Radical Trapping and Inhibition Studies

Reactions were performed in the presence of TEMPO **5** or BHT **24** (Scheme 6). The use of TEMPO **5** (1.5 equiv) did not prevent the formation of fluorenone **11** but did significantly retard the reaction, with incomplete conversion after 110 min (90%). During this time, the NHPI catalyst **1** was degraded to unknown products, and no evidence for a fluorene-TEMPO adduct was observed by LCMS. TEMPO **5** is known to react with hypochlorous acid^66^; therefore, it is possible that **5** could sequester the hypochlorous acid generated in the reaction, resulting in a slower rate of chlorine dioxide formation, which leads to a slower oxidation process. Addition of BHT **24** (1.5 equiv) inhibited the reaction, and no fluorenone **11** was formed. To probe this further, a reaction was performed in the presence of 5,5-dimethylpyrroline-*N*-oxide **25**. In this case, no fluorenone **11** was observed, and the reaction returned only fluorene starting material **10**. The reaction between 5,5-dimethylpyrroline-*N*-oxide **25** and chlorine dioxide has been studied previously, showing chlorine dioxide to be capable of oxidizing **25** to the equivalent amide *N*-oxide.^67^ The inhibition of the reaction by **25** is consistent with the essential role of chlorine dioxide in the reaction mechanism. At this stage, in the absence of evidence for the formation of a radical adduct, it could not be determined conclusively that the reaction proceeded by a radical pathway. The innovative radical trapping reagent CHANT **26** was recently introduced to detect short-lived radical species that have been elusive using other techniques.^68^ A reaction was set up under standard conditions (**10**, NaClO_2_ 1.5 equiv, NHPI **1** 10 mol %, acetic acid 1.0 equiv in MeCN/H_2_O (2:1)) and allowed to react for 30 min to ensure completion of the induction period, after which time CHANT **26** (5 mol %) was added and the reaction mixture was sampled and analyzed by HRMS after 5, 15, 30, and 60 min and again after 19 h. In each sample, CHANT **26** was detected in the reaction mixture, indicating that this reagent is uniquely stable to chlorine dioxide, unlike the other radical trapping reagents examined. In addition, the adduct **27**, formed between the PINO radical **2** and CHANT **26**, was detected in all samples by mass spectrometry. This is in contrast to the EPR experiment in which the PINO radical **2** was not detected, indicating that the radical is present but at concentrations too low to be detected by EPR. This result is consistent with the proposed mechanism that proceeds through the PINO radical **2** and highlights a distinct advantage of **26** as a radical trap compared to more traditional reagents.

**Figure 8:**
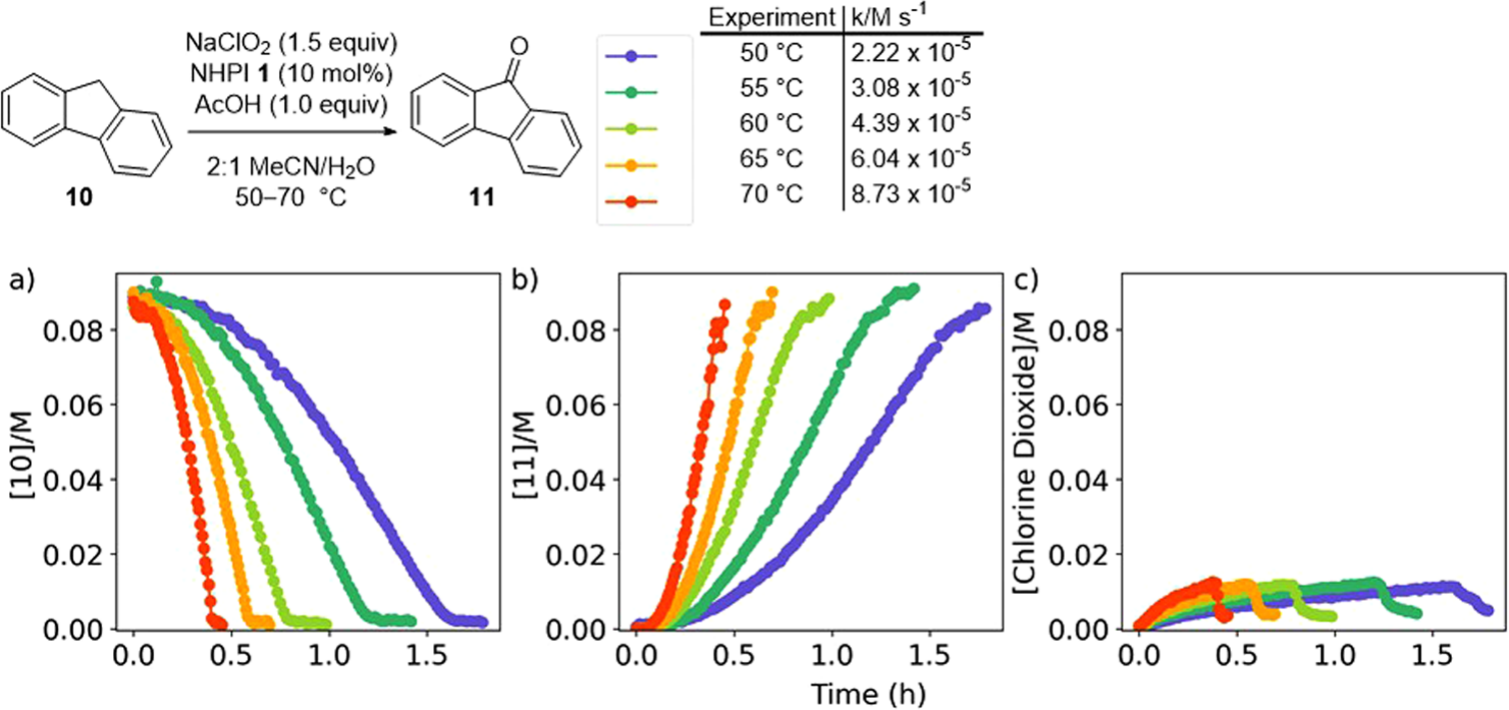
Monitoring fluorene **10** oxidation was performed by Raman spectroscopy. (a) Consumption of fluorene **10**. (b) Formation of fluorenone **11**. (c) Formation of chlorine dioxide.

The reaction between sodium chlorite and fluorene **10** could be conveniently studied by Raman spectroscopy that allowed simultaneous monitoring of the substrate **10**, the product **11**, and chlorine dioxide (Figure 8). The reaction was performed at temperatures between 50 and 70 °C, with higher temperatures resulting in an increase in reaction rate. Although the concentrations of chlorine dioxide present within the reaction were low throughout (maximum yield of chlorine dioxide of ca. 9% relative to the chlorite charge), the concentration steadily increased throughout the transformation when unreacted fluorene **10** was still present, indicating that the chlorine dioxide was formed at a faster rate than it was consumed by the oxidation process. Once the reaction was complete and all of the fluorene **10** had been consumed, the remaining chlorine dioxide decayed rapidly.

**Figure 9:**
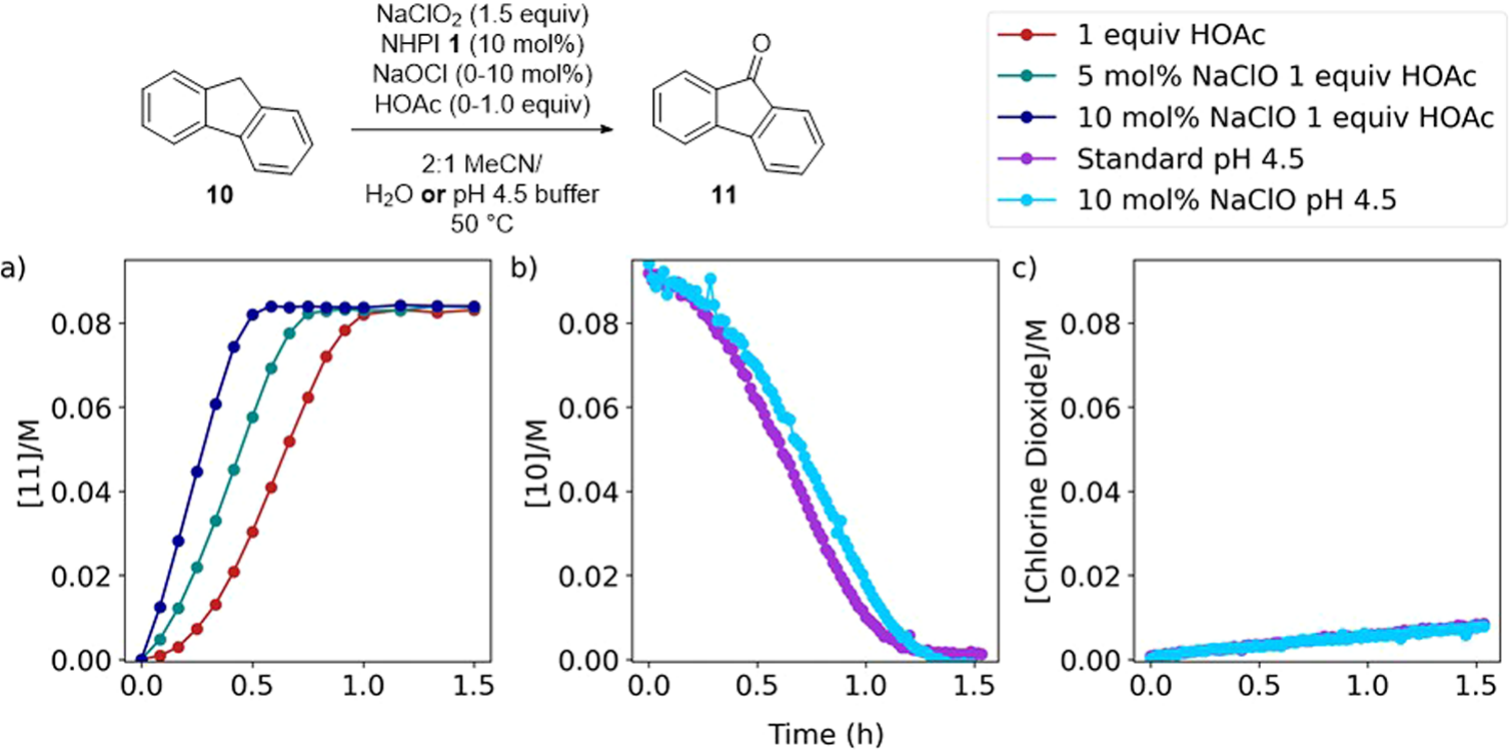
Effect of NaClO on the reaction between fluorene **10** and sodium chlorite in the presence of NHPI **1**, pH 4.5 buffer. (a) Concentration of **11** measured by HPLC. **10**, NaClO_2_ 1.5 equiv, NHPI **1** 10 mol %, AcOH 1.0 equiv in MeCN/H_2_O (2:1) with 0, 5, or 10 mol % NaClO. (b) Concentration of **10** measured by Raman spectroscopy. **10**, NaClO_2_ 1.5 equiv, NHPI **1** 10 mol % in MeCN/pH 4.5 aqueous buffer (2:1) with 0 or 10 mol % NaClO. (c) Concentration of chlorine dioxide measured by Raman spectroscopy. **10**, NaClO_2_ 1.5 equiv, NHPI **1** 10 mol % in 2:1 MeCN/pH 4.5 aqueous buffer (2:1) with 0 or 10 mol % NaClO.

The effect of sodium hypochlorite on the reaction was examined by Raman spectroscopy and HPLC. Under the standard reaction conditions (**10**, NaClO_2_ 1.5 equiv, NHPI **1** 10 mol %, AcOH 1.0 equiv in MeCN/H_2_O (2:1)), adding 5 or 10 mol % NaClO resulted in an increased rate of reaction and a decreased induction period (Figure 9a). Considering the pH sensitivity of the reaction, the reaction was repeated in a pH 4.5 buffer as the aqueous component and monitored by Raman spectroscopy (Figure 9b). Interestingly, under these conditions, the reaction time was longer than without the addition of bleach due to an extended induction period. We believe that the increased rate of reaction under nonbuffered conditions (Figure 9a) is a result of the pH sensitivity of the reaction and an increase in pH upon the addition of NaClO. When the pH of the aqueous solvent was buffered to 4.5, the formation of chlorine dioxide followed an identical profile upon the addition of NaClO (Figure 9c). These results indicate that hypochlorous acid is not responsible for the apparent autocatalytic reaction profile, and we believe that the induction period is due to the low concentration of chlorine dioxide and hence PINO radical **2** at the start of the reaction.

**Figure 10:**
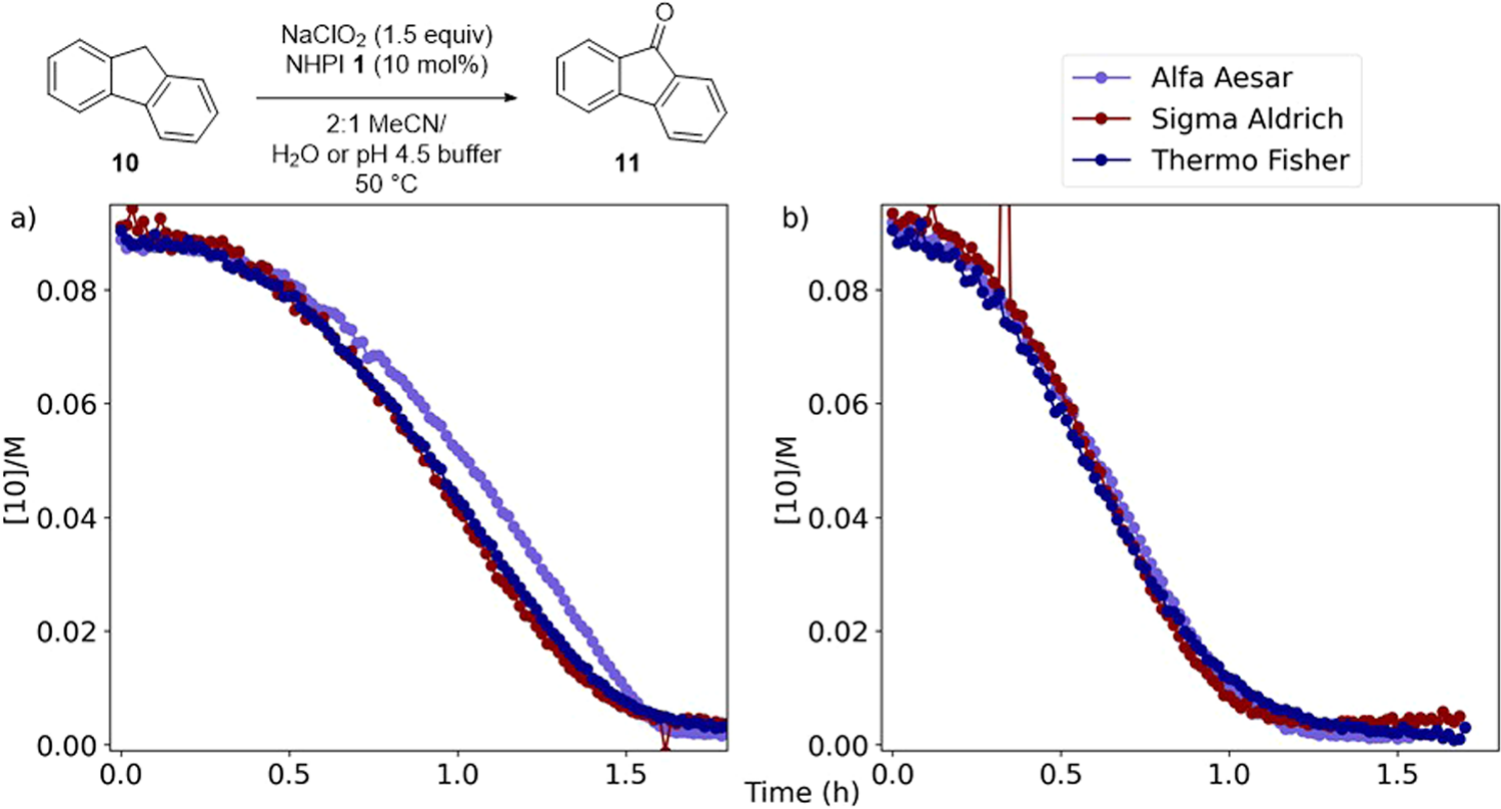
Rate of the reaction with different commercial suppliers of sodium chlorite. (a) Reactions using AcOH (1.0 equiv) to modulate pH. (b) Reactions using pH 4.5 acetate buffer as cosolvent.

During the investigation, it became apparent that sodium chlorite from different commercial suppliers performed differently in the transformation. Sodium chlorite is commonly supplied with 80% purity and was used directly without further purification. Depending upon the supplier, the composition of the remaining 20% varied, and this information was not always accessible. Reactions performed with samples of sodium chlorite from different chemical suppliers (Alfa Aesar, Sigma-Aldrich, Thermo Fisher) under standard conditions (**10**, NaClO_2_ 1.5 equiv, NHPI **1** 10 mol %, AcOH 1.0 equiv in MeCN/H_2_O (2:1), 50 °C) resulted in reactions that proceeded at different rates (Figure 10a). Each reaction produced a similar maximum concentration of chlorine dioxide (see the Supporting Information for full details). Iodometric titration of each sample showed similar levels of chlorite content (78–82%), well within the specifications of the supplier. Pleasingly, by performing the reactions in MeCN/pH 4.5 buffer (2:1), similar reaction profiles were obtained for different batches of the oxidant (Figure 10b), providing a simple solution to reproducibility issues arising from this process. It is expected that the different commercial chlorite samples contain varying amounts of an inorganic base. By performing the reaction in a buffered solution, the effects of these additives on pH and hence reaction rate were negated. Under the buffered reaction conditions, the maximum concentration of chlorine dioxide observed was slightly decreased relative to the standard conditions containing acetic acid (1.0 equiv) (see the Supporting Information for full details).

## Conclusions

In summary, we have presented a detailed investigation of an NHPI catalyzed benzylic oxidation using sodium chlorite as the stoichiometric oxidant. Through a combination of spectroscopic and experimental techniques along with electronic structure calculations, we have gained strong evidence for the mechanistic course of the transformation. Conducting the reaction in the presence of acetic acid (1.0 equiv) slowed the overall reaction rate but enabled the process to reach completion by prolonging the lifetime of the NHPI catalyst **1**. The reaction proceeds by the conversion of sodium chlorite into the active oxidant chlorine dioxide that is accelerated by hypochlorite, a coproduct of the transformation. The induction period that is observed at the beginning of the reaction can be attributed to the low initial concentration of chlorine dioxide and the active PINO radical catalyst **2**. Chlorine dioxide has two primary roles in the transformation: It is able to oxidize the PINO anion **20** to the PINO radical **2** that can abstract a hydrogen atom from the substrate to give the benzylic radical **9**. The radical species then combines with a second molecule of chlorine dioxide to form intermediate chlorous acid ester **21**, which undergoes elimination or pericyclic fragmentation to yield product ketone **8**, completing the catalytic cycle. Evidence for the presence of chlorine dioxide within the reaction mixture was obtained from UV/vis spectroscopy and EPR experiments. The PINO radical **2** could not be detected by these techniques under the optimized reaction conditions; however, it was possible to generate and detect this species independently and show that it was able to induce the transformation. In addition, the presence of **2** was confirmed by a reaction carried out with radical trapping agent CHANT **26**. Raman spectroscopy proved to be a powerful tool to study the process, being able to simultaneously detect chlorine dioxide, substrate **10**, and product **11** and providing the opportunity to determine kinetic data for the reaction. The progress of reactions conducted in acetonitrile/water mixtures varied depending on the commercial source of the sodium chlorite reagent, which could be overcome by conducting the transformation in an acetonitrile/pH 4.5 buffer, providing consistent and repeatable results. Of specific note about this process, which was originally described by Silvestre and Salvador,^44^ are the benign coproducts sodium chloride and water that are generated within the reaction, providing a green and sustainable benzylic oxidation process.

## Experimental Section

### General Procedure

Substrate (1.0 equiv), *N-*hydroxyphthalimide **1** (10 mol %), and acetic acid (1.0 equiv) were added to acetonitrile (0.125 M in substrate) and heated to 50 °C using an oil bath. A 0.375 M aqueous solution of sodium chlorite (80% technical grade, 1.5 equiv) was added dropwise, and the mixture was stirred until complete consumption of substrate by TLC. The mixture was allowed to cool to room temperature, excess oxidant was quenched via the addition of 10% (w/v) aqueous metabisulfite, and the acid was neutralized with saturated aqueous sodium bicarbonate solution. The mixture was extracted three times with TBME or diethyl ether. The combined organic phases were washed with brine, dried over MgSO_4_, filtered, and concentrated.

#### Fluorenone **11**

Fluorenone **11** was prepared according to the general procedure (1.78 g, 9.85 mmol, 98%) as a yellow solid. Mp 83–84 °C [lit: ^44^ 83–84 °C]. ^1^H NMR (400 MHz, CDCl_3_): δ 7.64 (dt, *J* = 7.4, 1.0 Hz, 2H), 7.53–7.42 (m, 4H), 7.27 (td, *J* = 7.2, 1.5 Hz, 2H). ^13^C{^1^H} NMR (101 MHz, CDCl_3_): δ 194.0, 144.5, 134.8, 134.2, 129.2, 124.4, 120.4. LCMS (formic): *t*_R_ = 1.14 min, [M + H^+^] = 181 (100% purity). Analytical data are consistent with those previously reported.^44^

#### Bis(1,3-dioxoisoindolin-2-yl) Phthalate **12**

To a 100 mL round-bottom flask were added *N*-hydroxyphthalimide **1** (500 mg, 3.07 mmol), acetonitrile (25 mL), and acetic acid (0.35 mL, 6.13 mmol). The mixture was warmed to 50 °C. In a separate vessel, sodium chlorite (693 mg, 6.13 mmol) was dissolved in water (12 mL). The sodium chlorite solution was then added to the prewarmed reaction mixture. The reaction was allowed to stir for 4 h, over which time a precipitate formed. Excess chlorite was quenched by the dropwise addition of 10% w/v sodium metabisulfite until the reaction mixture became colorless (∼1 mL). The reaction mixture was allowed to cool to room temperature and diluted with water (15 mL). The suspension was filtered, and the filtrate was washed with water (3 × 10 mL). The solid was dried *in vacuo* at 40 °C for 24 h to yield the *title compound***12** (260 mg, 0.57 mmol, 56%) as a white solid. Mp 229–231 °C [lit: ^49^ 229–233 °C]. ^1^H NMR (400 MHz, CDCl_3_) δ 8.10–8.20 (m, 2H), 7.85–7.95 (m, 4H), 7.77–7.87 (m, 6H). ^13^C{^1^H} NMR (101 MHz, CDCl_3_) δ: 162.6, 161.7, 134.9, 133.4, 131.1, 129.2, 127.8, 124.2. LCMS (formic) *t*_R_ = 1.21 min, [M + NH_4_^+^] 474.01 (>99% purity). Analytical data are consistent with those previously reported.^49^

#### Phthalide **23**

Phthalide **23** was prepared according to the general procedure to yield *title compound***23** (102 mg, 0.76 mmol, 76%) as a white solid. Mp 71–72 °C [lit: ^69^ 72–73 °C]. ^1^H NMR (400 MHz, CDCl_3_) δ 7.93 (dd, *J* = 7.5, 1.0 Hz, 1H), 7.69 (td, *J* = 7.5, 1.0 Hz, 1H), 7.54 (td, *J* = 7.5, 1.0 Hz, 1H), 7.50 (dt, *J* = 7.5, 1.0 Hz, 1H), 5.33 (s, 2H). ^13^C{^1^H} NMR (101 MHz, CDCl_3_) δ: 171.2, 146.7, 134.1, 129.2, 126.0, 125.9, 122.2, 69.8. GCMS (EI): *t*_R_ = 9.83 min, [M^+^] = 134.1. Analytical data are consistent with those previously reported.^69^

#### Electronic Calculations

Calculations were conducted using the GAUSSIAN16 software package. Geometry optimization followed by frequency calculations was performed using UM06-2X-D3/6-311++G(d,p) with the C-PCM acetonitrile implicit solvation model. Optimized structures were confirmed as energy minima by the absence of imaginary frequencies in the vibrational analysis. Transition states were confirmed as first-order saddle points on the potential energy surface by the presence of only one imaginary frequency in the vibrational analysis. Calculated transition states were confirmed as true by following the intrinsic reaction coordinate (IRC).

#### UV/vis Experiments

Standard solutions of fluorene **10** (0.0169 M), NHPI **1** (0.0067 M), acetic acid (0.0672 M), sodium chlorite (0.1008 M), and sodium hypochlorite (0.0067 M) in 2:1 MeCN/H_2_O were prepared. Aliquots were taken of each solution as required, added to a quartz cuvette, and diluted with 2:1 MeCN/H_2_O to give the final concentration (total volume 1.4 mL). Spectra were recorded on a Varian Cary 50 UV–vis spectrophotometer. The temperature was controlled using a Cary Single Cell Peltier accessory.

#### EPR Experiments

Standard solutions of phthalan **22** (0.125 M), NHPI **1** (0.012 M), acetic acid (0.125 M) in MeCN, aqueous sodium chlorite (0.375 M), chlorine dioxide in 2:1 MeCN/H_2_O (4.0 × 10^–4^ M), and NHPI **1** (0.008 M) in 2:1 MeCN/H_2_O were prepared. Spectra were collected on a Bruker ELEXSYS E500 spectrometer, and simulations were performed using Bruker’s XSophe software package.^70^

#### Raman Experiments

A solution of fluorene **10** (0.125 M), NHPI **1** (0.012 M), acetic acid (0.125 M), and 1,4-dioxane (0.129 M) in MeCN was added to a 250 mL jacketed vessel. The mixture was heated to 50 °C by using a recirculating water bath. The Raman probe was positioned on the side of the vessel, and the vessel was covered in blackout fabric. Aqueous sodium chlorite (0.375 M) was added to the mixture via a dropping funnel, and Raman spectra were acquired using a Kaiser RXN1 Raman spectrometer with a 785 nm laser fiber-coupled to a PhAT probe with a collimated beam and 6 mm diameter laser spot every 30 or 60 s. The spectra were analyzed between 700 and 1650 cm^–1^. The raw data were processed by taking a first derivative, applying a Savitzky–Golay filter (window length = 21, polynomial order = 2, as implemented in SciPy), and scaling the signal intensity by the 1,4-dioxane signal. The concentration of fluorene **10**, fluorenone **11**, and chlorine dioxide was determined based on the relative intensity of diagnostic signals to the 1,4 dioxane signal at 829.80 cm^–1^. Each experiment was conducted in duplicate, and the results were averaged to give a time course.

## Data Availability

The data underlying this study are available in the published article and its Supporting Information.

## Supporting Information Available

Additional experimental details and computational methods along with copies of HPLC traces, mass spectra, and NMR spectra (PDF)

## References

[ref1] Anastas P. T.; Warner J. C. Green chemistry: theory and practice. Oxford University Press: New York, 1994.

[ref2] Horváth I. T.; Anastas P. T. Innovations and Green Chemistry. Chem. Rev. 2007, 107, 2167–2173. 10.1021/cr078380v. 17564478

[ref3] Anastas P. T.; Eghbali N. Green Chemistry: Principles and Practice. Chem. Soc. Rev. 2010, 39, 301–312. 10.1039/B918763B. 20023854

[ref4] Modern Oxidation Methods, Bäackvall J.-E., Ed.; Wiley-VCH: Weinheim, Germany, 2004.

[ref5] Beller M. The Current Status and Future Trends in Oxidation Chemistry. Adv. Synth. Catal. 2004, 346, 107–108. 10.1002/adsc.200404008.

[ref6] Caron S.; Dugger R. W.; Ruggeri S. G.; Ragan J. A.; Ripin D.H. B. Large-Scale Oxidations in the Pharmaceutical Industry. Chem. Rev. 2006, 106, 2943–2989. 10.1021/cr040679f. 16836305

[ref7] Hermans I.; Spier E. S.; Neuenschwander U.; Turra N.; Baiker A. Selective Oxidation Catalysis: Opportunities and Challenges. Top. Catal. 2009, 52, 1162–1174. 10.1007/s11244-009-9268-3.

[ref8] Ahluwalia V. K. Oxidation in Organic Synthesis; CRC Press, 2012.

[ref9] Chen K.; Zhang P.; Wang Y.; Li H. Metal-free allylic/benzylic oxidation strategies with molecular oxygen: recent advances and future prospects. Green Chem. 2014, 16, 2344–2374. 10.1039/c3gc42135j.

[ref10] Landaeta V. R.; Rodriguez-Lugo R. E. Catalytic Aerobic Oxidations: Aerobic Oxidation Reactions in the Fine Chemicals and Pharmaceutical Industries. *In:* Catalytic Aerobic Oxidations. Mejía E., Ed. Royal Society of Chemistry: Cambridge, UK*:* 2020. *pp.* 252–290.

[ref11] Leifert D.; Studer A. Organic Synthesis Using Nitroxides. Chem. Rev. 2023, 123, 10302–10380. 10.1021/acs.chemrev.3c00212. 37578429

[ref12] Wang Y.; Yao J.; Li H. Aerobic oxidations via organocatalysis: A mechanistic perspective. Synthesis 2022, 54, 535–544. 10.1055/a-1661-6124.

[ref13] Lopat’eva E. R.; Krylov I. B.; Lapshin D. A.; Terent’ev A. O. Redox-active molecules as organocatalysts for selective oxidative transformations – an unperceived organocatalysis field. Beilstein J. Org. Chem. 2022, 18, 1672–1695. 10.3762/bjoc.18.179. 36570566 PMC9749543

[ref14] Cao Q.; Dornan L. M.; Rogan L.; Hughes N. L.; Muldoon M. J. Aerobic oxidation catalysis with stable radicals. Chem. Commun. 2014, 50, 4524–4543. 10.1039/C3CC47081D. 24667871

[ref15] Bretherick’s Handbook of Reactive Chemical Hazards (Eighth ed.), O1. Urben P. G., Ed.; Elsevier, 2017, 1094.

[ref16] Bretherick’s Handbook of Reactive Chemical Hazards (Eighth ed.), C1. Urben P. G., Ed.; Elsevier, 2017, 81–882.

[ref17] Cohn L. Phtalylhydroxylamin: Ueberführung der Phtalsäure in Salicylsäure. Justus Liebigs Ann. Chem. 1880, 205, 295–314. 10.1002/jlac.18802050304.

[ref18] Bhardwaj M.; Grover P.; Rasool B.; Mukherjee D. Recent Advances in N-Hyrdoxypthalimide: As a Free Radical Initiator and its Applications. Asian J. Org. Chem. 2022, 11, e202200442 10.1002/ajoc.202200442.

[ref19] Melone L.; Punta C. Metal-free aerobic oxidations mediated by N-hydroxyphthalimide. A concise review. Beilstein J. Org. Chem. 2013, 9, 1296–1310. 10.3762/bjoc.9.146. 23843925 PMC3701383

[ref20] Coseri S. Phthalimide-*N*-oxyl (PINO) Radical, a Powerful Catalytic Agent: Its Generation and Versatility Towards Various Organic Substrates. Catal. Rev. 2009, 51, 218–292. 10.1080/01614940902743841.

[ref21] Recupero F.; Punta C. Free Radical Functionalization of Organic Compounds Catalyzed by *N*-Hydroxyphthalimide. Chem. Rev. 2007, 107, 3800–3842. 10.1021/cr040170k. 17848093

[ref22] Rowlands G. J. Radicals in organic synthesis. Part 1. Tetrahedron 2009, 65, 8603–8655. 10.1016/j.tet.2009.07.001.

[ref23] Rowlands G. J. Radicals in organic synthesis. Part 2. Tetrahedron 2010, 66, 1593–1636. 10.1016/j.tet.2009.12.023.

[ref24] Yan M.; Lo J. C.; Edwards J. T.; Baran P. S. Radicals: Reactive Intermediates with Translational Potential. J. Am. Chem. Soc. 2016, 138, 12692–12714. 10.1021/jacs.6b08856. 27631602 PMC5054485

[ref25] Hung K.; Hu X.; Maimone T. J. Total synthesis of complex terpenoids employing radical cascade processes. Nat. Prod. Rep. 2018, 35, 174–202. 10.1039/C7NP00065K. 29417970 PMC5858714

[ref26] Inoue M. Evolution of Radical-Based Convergent Strategies for Total Syntheses of Densely Oxygenated Natural Products. Acc. Chem. Res. 2017, 50, 460–464. 10.1021/acs.accounts.6b00475. 28945405

[ref27] Smith J. M.; Harwood S. J.; Baran P. S. Radical Retrosynthesis. Acc. Chem. Res. 2018, 51, 1807–1817. 10.1021/acs.accounts.8b00209. 30070821 PMC6349421

[ref28] Romero K. J.; Galliher M. S.; Pratt D. A.; Stephenson C. R. J. Radicals in natural product synthesis. Chem. Soc. Rev. 2018, 47, 7851–7866. 10.1039/C8CS00379C. 30101272 PMC6205920

[ref29] Pitre S. P.; Weires N. A.; Overman L. E. Forging C(sp3)–C(sp3) Bonds with Carbon-Centered Radicals in the Synthesis of Complex Molecules. J. Am. Chem. Soc. 2019, 141, 2800–2813. 10.1021/jacs.8b11790. 30566838 PMC6511262

[ref30] Crespi S.; Fagnoni M. Generation of Alkyl Radicals: From the Tyranny of Tin to the Photon Democracy. Chem. Rev. 2020, 120, 9790–9833. 10.1021/acs.chemrev.0c00278. 32786419 PMC8009483

[ref31] Pitre S. P.; Overman L. E. Strategic Use of Visible-Light Photoredox Catalysis in Natural Product Synthesis. Chem. Rev. 2022, 122, 1717–1751. 10.1021/acs.chemrev.1c00247. 34232019

[ref32] Kaczur J. J.; Cawlfield D. W. Chlorine Oxygen Acids and Salts, Chlorous Acid, Chlorites, and Chlorine Dioxide. Kirk-Othmer Encycl. Chem. Technol. 2000, 1. 10.1002/0471238961.0308121511010326.a01

[ref33] Taylor M. C.; Whitte J. F.; Vincent G. P.; Cunnigham G. I. Sodium chlorite properties and reactions. Ind. Eng. Chem. 1940, 32, 899–903. 10.1021/ie50367a007.

[ref34] Aieta E. M.; Berg J. D. A Review of Chlorine Dioxide in Drinking Water Treatment. J. Am. Water Work. Assoc. 1986, 78, 62–72. 10.1002/j.1551-8833.1986.tb05766.x.

[ref35] Krapcho A. P. Uses of sodium chlorite and sodium bromate in organic synthesis. A review. Org. Prep. Proced. Int. 2006, 38, 177–216. 10.1080/00304940609355988.

[ref36] Hase T.; Wahala K. Sodium Chlorite. in Encyclopedia of Reagents for Organic Synthesis, Paquette L. A., Ed., Vol. 7, p 4533, John Wiley & Sons: New York, 1995.

[ref37] Lindgren B. O.; Nilsson T.; Husebye S.; Mikalsen Ø.; Leander K.; Swahn C. G. Preparation of Carboxylic Acids from Aldehydes (Including Hydroxylated Benzaldehydes) by Oxidation with Chlorite. Acta Chem. Scand. 1973, 27, 888–890. 10.3891/acta.chem.scand.27-0888.

[ref38] Kraus G. A.; Roth B. Synthetic studies toward verrucarol. 2. Synthesis of the AB ring system. J. Org. Chem. 1980, 45, 4825–4830. 10.1021/jo01312a004.

[ref39] Kraus G. A.; Taschner M. J. Model studies for the synthesis of quassinoids. 1. Construction of the BCE ring system. J. Org. Chem. 1980, 45, 1175–1176. 10.1021/jo01294a058.

[ref40] Bal B. S.; Childers W. E.; Pinnick H. W. Oxidation of α,β-unsaturated aldehydes. Tetrahedron 1981, 37, 2091–2096. 10.1016/S0040-4020(01)97963-3.

[ref41] Mohamed M. A.; Yamada K.-i.; Tomioka K. Accessing the amide functionality by the mild and low-cost oxidation of imine. Tetrahedron Lett. 2009, 50, 3436–3438. 10.1016/j.tetlet.2009.02.174.

[ref42] Zhao M.; Li J.; Mano E.; Song Z.; Tschaen D. M.; Grabowski E. J. J.; Reider P. J. Oxidation of Primary Alcohols to Carboxylic Acids with Sodium Chlorite Catalyzed by TEMPO and Bleach. J. Org. Chem. 1999, 64, 2564–2566. 10.1021/jo982143y.

[ref43] Imaizumi N.; Kanayama T.; Oikawa K. Effect of dimethylsulfoxide as a masking agent for aqueous chlorine in the determination of oxychlorines. Analyst 1995, 120, 1983–1987. 10.1039/an9952001983.

[ref44] Silvestre S. M.; Salvador J. A. R. Allylic and benzylic oxidation reactions with sodium chlorite. Tetrahedron 2007, 63, 2439–2445. 10.1016/j.tet.2007.01.012.

[ref45] Ishii Y.; Sakaguchi S.; Iwahama T. Innovation of Hydrocarbon Oxidation with Molecular Oxygen and Related Reactions. Adv. Synth. Catal. 2001, 343, 393–427. 10.1002/1615-4169(200107)343:5<393::AID-ADSC393>3.0.CO;2-K.

[ref46] Sheldon R. A.; Arends I. W. C. E. Organocatalytic Oxidations Mediated by Nitroxyl Radicals. Adv. Synth. Catal. 2004, 346, 1051–1071. 10.1002/adsc.200404110.

[ref47] Yamaoka M.; Nakazaki A.; Kobayashi S. Total synthesis of fomitellic acid B. Tetrahedron Lett. 2009, 50, 6764–6768. 10.1016/j.tetlet.2009.09.088.

[ref48] Sasaki I.; Yamasaki N.; Kasai Y.; Imagawa H.; Yamamoto H. A synthetic protocol for (−)-ketorolac; development of asymmetric gold(I)-catalyzed cyclization of allyl alcohol with pyrrole ring core. Tetrahedron Lett. 2020, 61, 151564 10.1016/j.tetlet.2019.151564.

[ref49] UEDA C.; NOYAMA M.; OHMORI H.; MASUI M. Reactivity of Phthalimide-N-oxyl: A Kinetic Study. Chem. Pharm. Bull. 1987, 35, 1372–1377. 10.1248/cpb.35.1372.

[ref50] Amorati R.; Lucarini M.; Mugnaini V.; Pedulli G. F.; Minisci F.; Recupero F.; Fontana F.; Astolfi P.; Greci L. Hydroxylamines as Oxidation Catalysts: Thermochemical and Kinetic Studies. J. Org. Chem. 2003, 68, 1747–1754. 10.1021/jo026660z. 12608787

[ref51] Yang C.; Farmer L. A.; Pratt D. A.; Maldonado S.; Stephenson C. R. J. Mechanism of Electrochemical Generation and Decomposition of Phthalimide-N-oxyl. J. Am. Chem. Soc. 2021, 143, 10324–10332. 10.1021/jacs.1c04181. 34213314

[ref52] Zdilla M. J.; Lee A. Q.; Abu-Omar M. M. Concerted Dismutation of Chlorite Ion: Water-Soluble Iron-Porphyrins As First Generation Model Complexes for Chlorite Dismutase. Inorg. Chem. 2009, 48, 2260–2268. 10.1021/ic801681n. 19138154

[ref53] Friis S. D.; Lindhardt A. T.; Skrydstrup T. The Development and Application of Two-Chamber Reactors and Carbon Monoxide Precursors for Safe Carbonylation Reactions. Acc. Chem. Res. 2016, 49 (4), 594–605. 10.1021/acs.accounts.5b00471. 26999377

[ref54] Kieffer R. G.; Gordon G. Disproportionation of Chlorous Acid. II. Kinetics. Inorg. Chem. 1968, 7, 239–244. 10.1021/ic50060a014.

[ref55] Horváth A. K.; Nagypál I.; Peintler G.; Epstein I. R.; Kustin K. Kinetics and Mechanism of the Decomposition of Chlorous Acid. J. Phys. Chem. A 2003, 107, 6966–6973. 10.1021/jp027411h.

[ref56] Peintler G.; Nagypal I.; Epstein I. R. Systematic design of chemical oscillators. 60. Kinetics and mechanism of the reaction between chlorite ion and hypochlorous acid. J. Phys. Chem. 1990, 94, 2954–2958. 10.1021/j100370a040.

[ref57] Gambarotti C.; Punta C.; Recupero F.; Zlotorzynska M.; Sammis G. N-Hydroxyphthalimide In Encyclopedia of Reagents for Organic Synthesis 2013.

[ref58] Costentin C. Proton-Coupled Electron Transfer Catalyst: Homogeneous Catalysis. Application to the Catalysis of Electrochemical Alcohol Oxidation in Water. ACS Catal. 2020, 10, 6716–6725. 10.1021/acscatal.0c01195.

[ref59] Sutton S. C.; Cleland W. E.; Hammer N. I. Introducing Students to a Synthetic and Spectroscopic Study of the Free Radical Chlorine Dioxide. J. Chem. Educ. 2017, 94, 515–520. 10.1021/acs.jchemed.6b00599.

[ref60] Kushch O.; Hordieieva I.; Novikova K.; Litvinov Y.; Kompanets M.; Shendrik A.; Opeida I. Kinetics of N-Oxyl Radicals’ Decay. J. Org. Chem. 2020, 85, 7112–7124. 10.1021/acs.joc.0c00506. 32412243

[ref61] Annunziatini C.; Gerini M. F.; Lanzalunga O.; Lucarini M. Aerobic Oxidation of Benzyl Alcohols Catalyzed by Aryl Substituted N-Hydroxyphthalimides. Possible Involvement of a Charge-Transfer Complex. J. Org. Chem. 2004, 69, 3431–3438. 10.1021/jo049887y. 15132553

[ref62] Zhao Y.; Truhlar D. G. The M06 Suite of Density Functionals for Main Group Thermochemistry, Thermochemical Kinetics, Noncovalent Interactions, Excited States, and Transition Elements: Two New Functionals and Systematic Testing of Four M06-Class Functionals and 12 Other Functionals and Inorganometallic Chemistry and for Noncovalent Interactions. Theor. Chem. Acc. 2008, 120, 215–241. 10.1007/s00214-007-0310-x.

[ref63] Grimme S.; Antony J.; Ehrlich S.; Krieg H. A Consistent and Accurate Ab Initio Parametrization of Density Functional Dispersion Correction (DFT-D) for the 94 Elements H-Pu. J. Chem. Phys. 2010, 132, 154104 10.1063/1.3382344. 20423165

[ref64] Barone V.; Cossi M. Quantum Calculation of Molecular Energies and Energy Gradients in Solution by a Conductor Solvent Model. J. Phys. Chem. A 1998, 102, 1995–2001. 10.1021/jp9716997.

[ref65] Cossi M.; Rega N.; Scalmani G.; Barone V. Energies, Structures, and Electronic Properties of Molecules in Solution with the C-PCM Solvation Model. J. Comput. Chem. 2003, 24, 669–681. 10.1002/jcc.10189. 12666158

[ref66] Lucio Anelli P.; Biffi C.; Montanari F.; Quici S. Fast and selective oxidation of primary alcohols to aldehydes or to carboxylic acids and of secondary alcohols to ketones mediated by oxoammonium salts under two-phase conditions. J. Org. Chem. 1987, 52, 2559–2562. 10.1021/jo00388a038.

[ref67] Ozawa T.; Miura Y.; Ueda J.-I. Oxidation of spin-traps by chlorine dioxide (ClO_2_) radical in aqueous solutions: First ESR evidence of formation of new nitroxide radicals. Free Radic. Biol. Med. 1996, 20, 837–841. 10.1016/0891-5849(95)02092-6. 8728032

[ref68] Williams P. J. H.; Boustead G. A.; Heard D. E.; Seakins P. W.; Rickard A. R.; Chechik V. New approach to the detection of short-lived radical intermediates. J. Am. Chem. Soc. 2022, 144, 15969–15976. 10.1021/jacs.2c03618. 36001076 PMC9460783

[ref69] Okamoto K.; Sakata N.; Ohe K. Copper-Catalyzed Cyanation of Aryl- and Alkenylboronic Reagents with Cyanogen Iodide. Org. Lett. 2015, 17, 4670–4673. 10.1021/acs.orglett.5b01924. 26360959

[ref70] Hanson G. R.; Gates K. E.; Noble C. J.; Griffin M.; Mitchell A.; Benson S. XSophe-Sophe-XeprView®. A computer simulation software suite (v. 1.1.3) for the analysis of continuous wave EPR spectra. J. Inorg. Biochem. 2004, 98, 903–916. 10.1016/j.jinorgbio.2004.02.003. 15134936

