# Supplementary material for: A Mechanistic Investigation of the N-Hydroxyphthalimide Catalyzed Benzylic Oxidation Mediated by Sodium Chlorite
Source: J Org Chem. 2024 May 15;89(11):7933–45. doi: 10.1021/acs.joc.4c00583 (PMC11165572; doi:10.1021/acs.joc.4c00583)
Supplement: Supplementary file 1 — jo4c00583_si_001.pdf [file jo4c00583_si_001.pdf]

Supporting Information

A mechanistic investigation of the *N*-hydroxyphthalimide catalyzed benzylic oxidation mediated by sodium chlorite

Thomas Grunshaw,[†,§] Susanna H. Wood,[†] Stephen Sproules,[‡] Andrew Parrott,[†] Alison Nordon,[†] Peter D. P. Shapland,[§] Katherine M. P. Wheelhouse[§] and Nicholas C. O. Tomkinson*,[†]

[†]Department Pure and Applied Chemistry, Thomas Graham Building, University of Strathclyde, Glasgow, G1 1XL, U.K.

[‡]School of Chemistry, University Avenue, University of Glasgow, Glasgow, G12 8QQ, U.K.

[§]GlaxoSmithKline R&D, Gunnels Wood Road, Stevenage, SG1 2NY, U.K.

E-mail: Nicholas.Tomkinson@strath.ac.uk

Contents

General Experimental S3

Synthetic Procedures S5

pH Buffered Reactions S7

HPLC Calibration Curves S8

General Procedure for HPLC Monitored Reactions S9

Effect of stirring on reaction S10

Oxidation of fluorenol **16** S11

Oxidation of fluorene **10** under air *vs*. N_2 S12

Generation and reaction of [^{18}O]-ClO_2 with fluorene **10** S13

Inhibition of oxidation of fluorene **10** by DMSO S16

UV/vis Study S18

EPR Experiments S22

Radical Trapping Experiments S25

Raman Experiments S34

Computational Methods S46

Copies of NMR Spectra S78

References S81

General Experimental

Nuclear magnetic resonance (NMR) spectra were recorded on a Bruker Avance III 400 (^{1}H 400 MHz and ^{13}C 101 MHz), Avance III HD NanoBay (^{1}H 400 MHz and ^{13}C 101 MHz) or Bruker AV–400 (^{1}H 400 MHz, ^{13}C 101 MHz) spectrometer. NMR spectra were recorded at 25 °C. Chemical shifts are given in parts per million (ppm) referenced to residual protium or carbon of the solvents. Data for ^{1}H NMR spectra are reported as follows: chemical shift, multiplicity, coupling constant, and integration. Coupling constants (*J* values) are reported in Hertz (Hz) and multiplicities are expressed according to the usual conventions. LCMS analysis was performed on a Waters® Acquity UPLC instrument equipped with a BEH column (50.0 mm × 2.10 mm with 1.70 µm packing diameter) and a Waters® Micromass ZQ MS using alternate-scan positive and negative electrospray ionisation. Analytes were detected as a summed UV wavelength spectrum between 210–350 nm. Analysis was conducted at 40 °C with a flow rate of 1 mL/min. Three methods were used:

Formic acid: The eluents employed were: A: 0.1% *v/v* solution of formic acid in water, B: 0.1% *v/v* solution of formic acid in acetonitrile. The gradient employed was linear from 97:3 A:B to 5:95 A:B over 1.5 mins, held at 5:95 A:B for 0.4 mins before linear return to 97:3 A:B over 0.1 mins.

High pH: The eluents employed were: A: 10 mM ammonium bicarbonate in water adjusted to pH 10 with ammonia solution. B: Acetonitrile. The gradient employed was linear from 97:3 A:B to 5:95 A:B over 1.5 mins, held at 5:95 A:B for 0.4 mins before linear return to 97:3 A:B over 0.1 mins.

8-minute TFA: The eluents employed were: A: 0.1% *v/v* solution of trifluoroacetic acid (TFA) in water, B: 0.1% *v/v* solution of trifluoroacetic acid in acetonitrile. The gradient employed was linear 97:3 A:B to 3:97 A:B over 6 minutes, held at 3:97 A:B for 1.9 minutes before linear return to 97:3 A:B over 0.1 minutes.

HPLC was conducted on a Waters X-select CSH C18 column (XP) with 2.5 µm particle size (2.1 × 30 mm i.d.) at 40 °C, at a flow rate of 1 mL/min. The eluents employed were: A: 0.1% *v/v* solution of trifluoroacetic acid (TFA) in water, B: 0.1% *v/v* solution of trifluoroacetic acid in acetonitrile. The gradient was linear 100% A to 5:95 A:B over 8 minutes.

High-resolution mass spectra were recorded on a Micromass Q-Tof Ultima® time-of-flight mass spectrometer and analytes were separated on an Agilent® 1100 Liquid Chromatograph equipped with a Penomenex® Luna C18 (2) reversed phase column (100 mm × 2.1 mm, 3 µm packing diameter). Conditions used were 0.5 mL/min flow rate at 35 °C, and an injection volume of 2-5 µL. The eluents employed were: A: 0.1% *v/v* solution of formic acid in water, B: 0.1% *v/v* solution of formic acid in acetonitrile. The gradient employed was linear from 95:5 A:B to 100% B over 6 minutes, held at 100% B for 2.5 minutes, before a linear return to 95:5 A:B over 1 minute. Mass to charge (*m/z*) ratios are reported in Daltons (Da). GC-MS was performed using an Agilent 7890A GC system, equipped with a

30 m DB5MS column connected to a 5975C inert XL CI MSD with Triple-Axis Detector. When required, organic solvents were removed under reduced pressure. Evaporation was carried out at about 20 mmHg using Büchi rotary evaporators and water bath, followed by evaporation to dryness under high vacuum (4 mbar). Analytical thin-layer chromatography (TLC) was carried out using 0.2 mm commercial silica gel plates (silica gel 60, F254, EMD chemical), and visualised under UV light (at 254 nm) or by staining with a solution of 2% aqueous potassium permanganate followed by gentle heating. Melting points were determined on a Stuart SMP11 and are uncorrected. UV/vis spectra were recorded on a Varian Cary 50 UV-Vis spectrophotometer using quartz cuvettes. Raman spectra were acquired using a Kaiser RXN1 Raman spectrometer with a 785 nm laser, fibre-coupled to a PhAT probe with a collimated beam and 6 mm diameter laser spot. EPR spectroscopic analyses were conducted using a Bruker ELEXSYS E500 spectrometer, and simulations were performed using Bruker's Xsophe software.[1]

Synthetic Procedures

General Procedure – Oxidation Under Acidified Conditions. Substrate (1 equiv), *N*-hydroxyphthalimide **1** (10 mol%) and acetic acid (1 equiv) were added to acetonitrile (0.125 M in substrate) and heated to 50 °C. A 0.375 M aqueous solution of sodium chlorite (80% technical grade, 1.5 equiv.) was added dropwise and the mixture stirred until complete consumption of substrate by TLC. The mixture was allowed to cool to room temperature, excess oxidant quenched *via* the addition of 10% (*w/v*) aqueous metabisulfite and acid neutralised with saturated aqueous sodium bicarbonate solution. The mixture was extracted three times with TBME or diethyl ether. The combined organic phases were washed with brine, dried over $MgSO_4$, filtered and concentrated.

Fluorenone 11

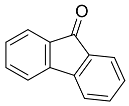

Prepared according to General Procedure (1.78 g, 9.85 mmol, 98% yield) as a yellow solid.

^{1}H NMR (400 MHz, $CDCl_3$): δ 7.64 (dt, *J* = 7.4, 1.0 Hz, 2H), 7.53–7.42 (m, 4H), 7.27 (td, *J* = 7.2, 1.5 Hz, 2H).

^{13}C{^{1}H} NMR (101 MHz, $CDCl_3$): δ 194.0, 144.5, 134.8, 134.2, 129.2, 124.4, 120.4.

LCMS (formic): t_R = 1.14 min, [M+H$^+$] = 181, (100% purity).

m.p.: 83–84 °C (lit.:[2] 80–82 °C)

Analytical data consistent with that previously reported.[2]

Phthalide 23

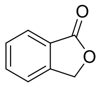

Prepared according to General Procedure, to yield *title compound* (102 mg, 0.76 mmol, 76%) as a white solid.

^{1}H NMR (400 MHz, $CDCl_3$): δ 7.93 (dd, *J* = 7.5, 1.0 Hz, 1H), 7.69 (td, *J* = 7.5, 1.0 Hz, 1H), 7.54 (td, *J* = 7.5, 1.0 Hz, 1H), 7.50 (dt, *J* = 7.5, 1.0 Hz, 1H), 5.33 (s, 2H).

^{13}C{^{1}H} NMR (101 MHz, $CDCl_3$): δ 171.2, 146.7, 134.1, 129.2, 126.0, 125.9, 122.2, 69.8.

GCMS (EI): t_R = 9.83 min, [M$^+$] = 134.1.

m.p.: 71–72 °C (lit.:[3] 72–73 °C)

Analytical data consistent with that previously reported.[3]

Bis(1,3-dioxoisoindolin-2-yl) phthalate 12

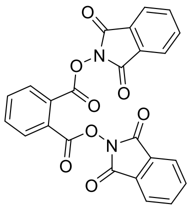

To a 100 mL round-bottom flask was added *N*-hydroxyphthalimide **1** (500 mg, 3.07 mmol), acetonitrile (25 mL) and acetic acid (0.35 mL, 6.13 mmol). The mixture was warmed to 50 °C. In a separate vessel, sodium chlorite (693 mg, 6.13 mmol) was dissolved in water (12 mL). The sodium chlorite solution was then added to the prewarmed reaction mixture. The reaction was allowed to stir for 4 hours, over which time a precipitate formed. Excess chlorite was quenched by the dropwise addition of 10% *w/v* sodium metabisulfite until the reaction mixture became colourless (~1 mL). The reaction mixture was allowed to cool to room temperature and diluted with water (15 mL). The suspension was filtered, and the filtrate washed with water (3 × 10 mL). The solid was dried *in vacuo* at 40 °C for 24 h to yield the *title compound* (260 mg, 0.57 mmol, 56% yield) as a white solid.

^{1}H NMR (400 MHz, $CDCl_3$): δ = 8.10–8.20 (m, 2H), 7.85–7.95 (m, 4H), 7.77–7.87 (m, 6H).

^{13}C{^{1}H} NMR (101 MHz, $CDCl_3$): δ = 162.6, 161.7, 134.9, 133.4, 131.1, 129.2, 127.8, 124.2.

LCMS (formic): t_R = 1.21 min, [M+NH_4^+] 474.01, (>99 % purity).

m.p.: 229–231 °C (lit.:[4] 229–233 °C)

Analytical data consistent with that previously reported.[4]

pH Buffered Reactions

Sodium chlorite (1.5 equiv) was dissolved in the appropriate buffer and the solutions added as single charges to an acetonitrile solution of fluorene **10** and NHPI **1** (10 mol%) maintained at 50 °C. The reactions were sampled at known times and were directly quenched into a 0.2 M solution of sodium metabisulfite in 2:1 THF/H_2O. Quenched samples were analyzed by LCMS to establish a qualitative picture of the transformation.

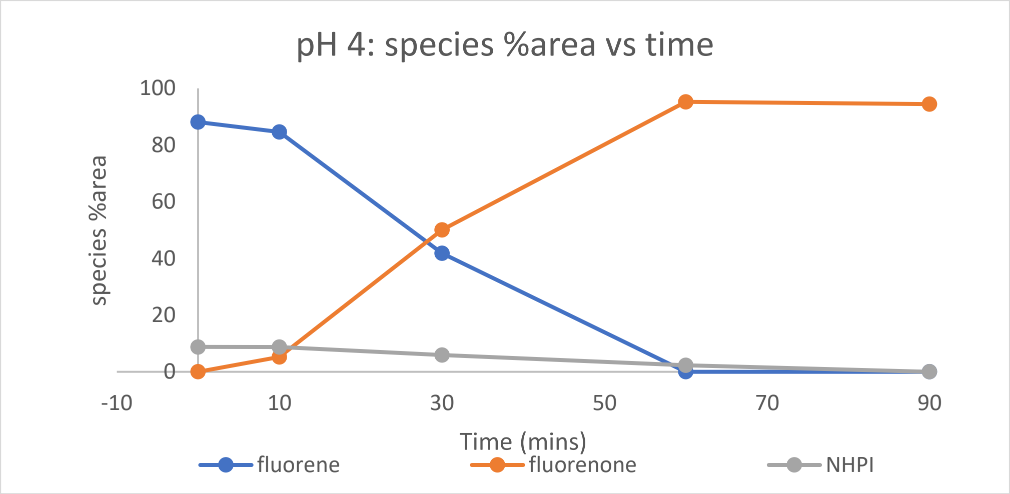

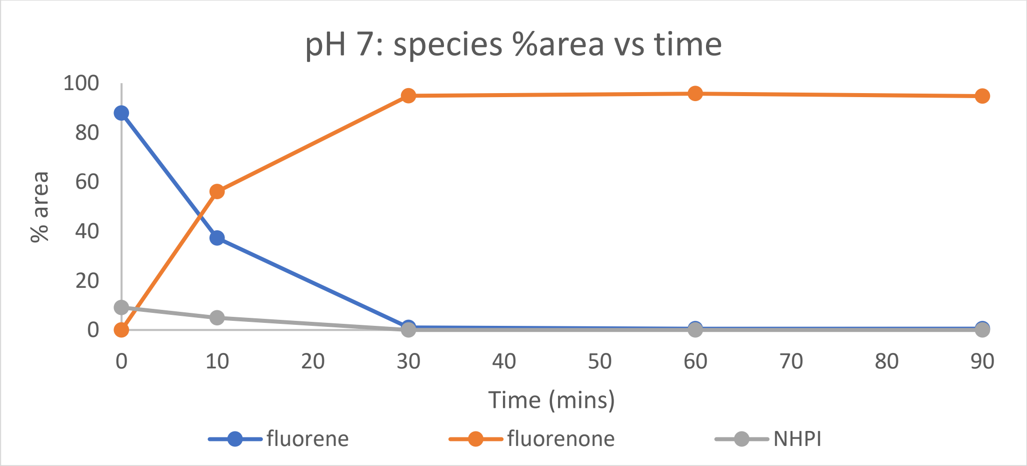

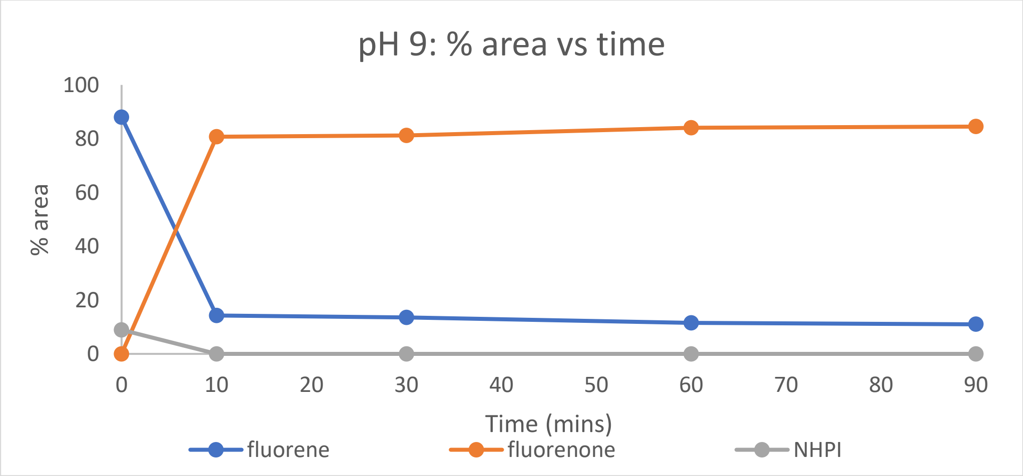

HPLC Calibration Curves

HPLC calibration curves showing the response at 220 nm for serial dilutions of standard solutions of fluorene **10**, fluorenone **11**, NHPI **1**, 1,4-dicyanobenzene, phthalic acid **13** and fluorenol **16**.

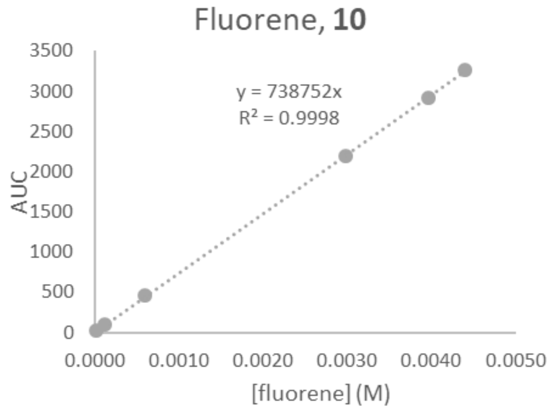

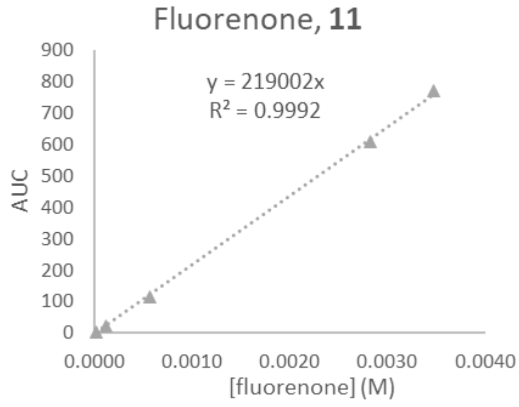

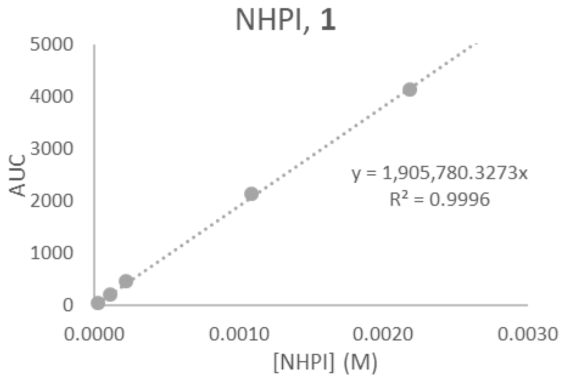

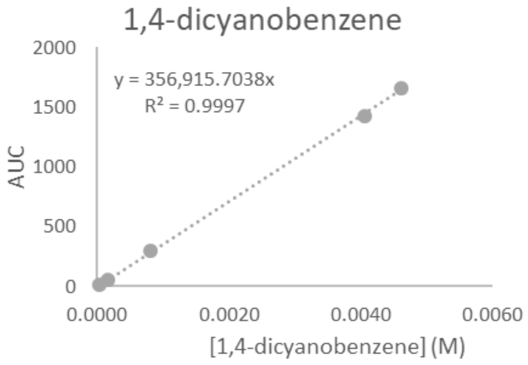

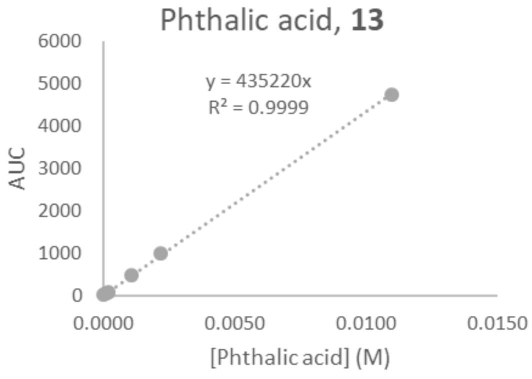

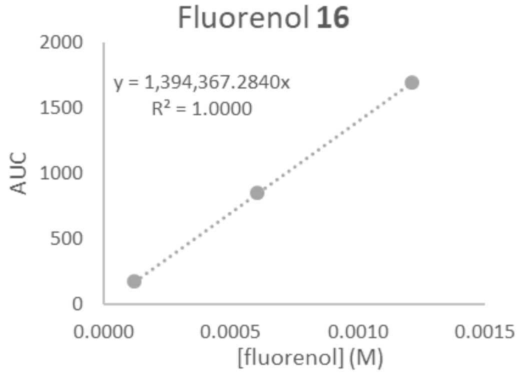

General Procedure for HPLC Monitored Reactions

To a 50 mL EasyMax vessel was added the substrate (4 mmol) and the required amounts of NHPI **1**, and 1,4-dicyanobenzene (internal standard, 103 mg, 0.8 mmol) with acetonitrile (32 mL) and the required amount of acetic acid. The vessel was assembled, and the mixture was heated to 50 °C (target solution temperature) in the EasyMax reactor.

An appropriate number of HPLC vials were prepared with 0.49 mL of a 0.02 M solution of sodium metabisulfite in 2:1 *v/v* THF/H_2O. Prior to starting the reaction, 10 µL of the reaction mixture was sampled and added to the first HPLC vial.

The required amount of aqueous sodium chlorite (16 mL) was added to the reaction mixture. Further samples were taken at 5-minute intervals over the first 60 minutes, and then every 10 minutes thereafter until the reaction was complete. Samples were analysed by HPLC (8-minute TFA method).

Effect of stirring on reaction

Reaction carried out according to general HPLC monitoring procedure on 0.5 mmol scale in 25 mL RBF: **10** 1 equiv, NHPI **1** 10 mol%, $NaClO_2$ 1.5 equiv, AcOH 1 equiv, $MeCN:H_2O$ (2:1), 50 °C.

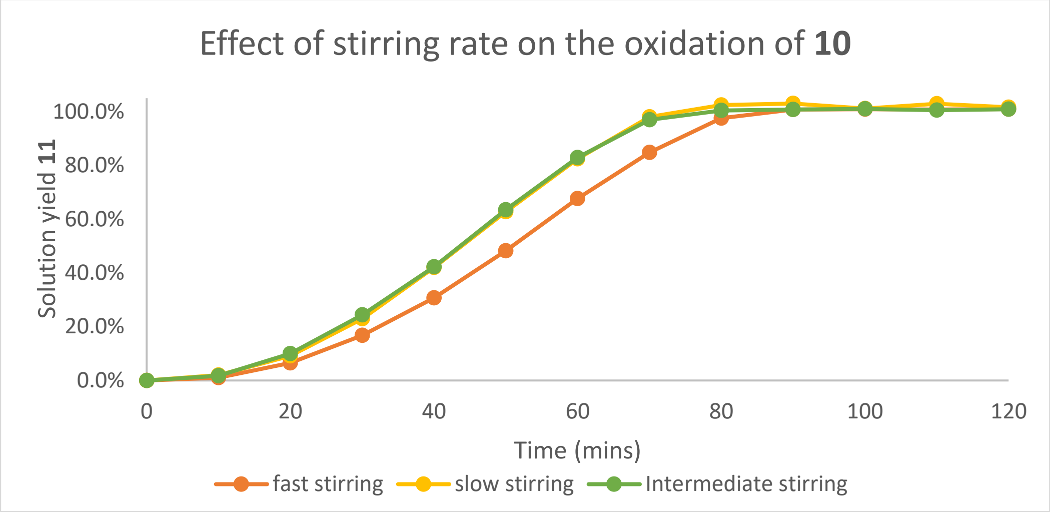

Reaction carried out according to general HPLC monitoring procedure on 0.5 mmol scale in EasyMax reactor: **10** 1 equiv, NHPI **1** 10 mol%, $NaClO_2$ 1.5 equiv, AcOH 1 equiv, $MeCN:H_2O$ (2:1), 50 °C.

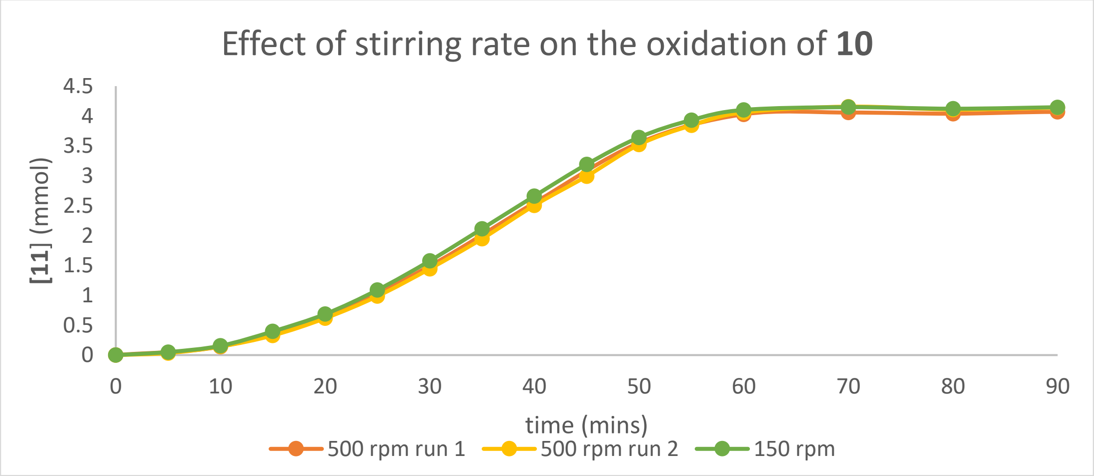

Oxidation of fluorenol 16

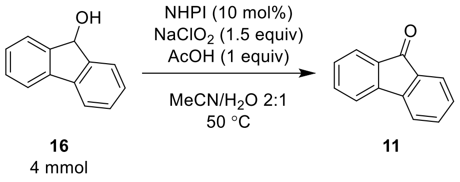

Reaction carried out according to general HPLC monitoring procedure. **16** 4 mmol, NHPI **1** 10 mol%, $NaClO_2$ 1.5 equiv, AcOH 1 equiv, MeCN:H_2O (2:1), 50 °C.

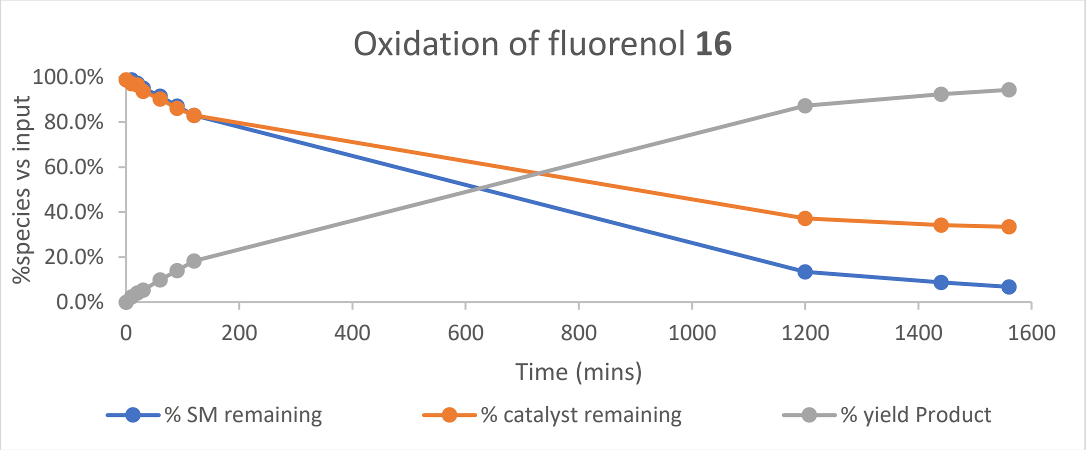

Oxidation of fluorene 10 under air vs. N_2

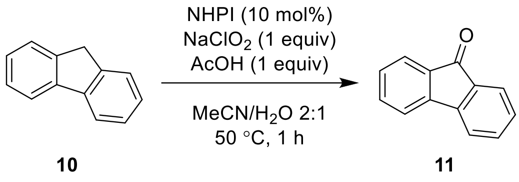

Reaction carried out according to general HPLC monitoring procedure under air or nitrogen atmosphere.

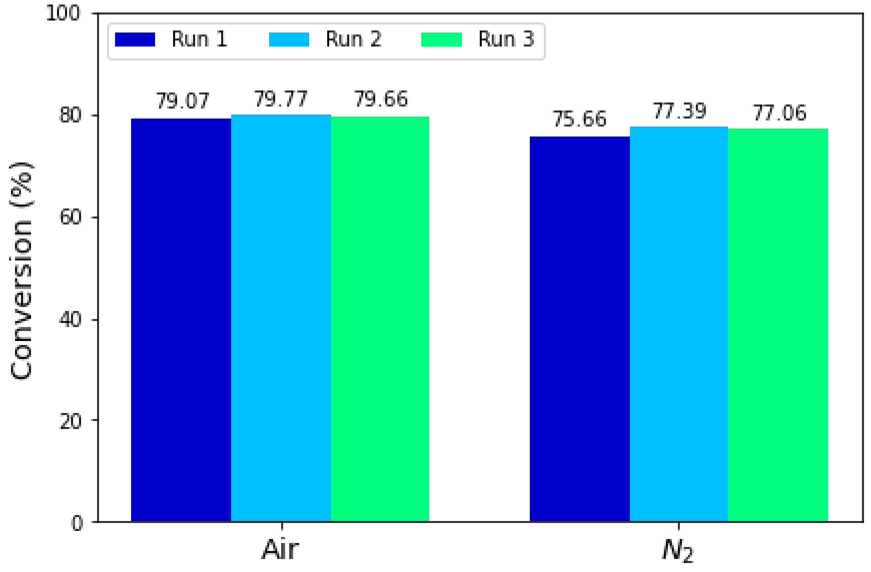

Generation and reaction of [^{18}O]-ClO_2 with fluorene 10

Procedure for the generation of [^{18}O]-$NaClO_2$ was adapted.[5]

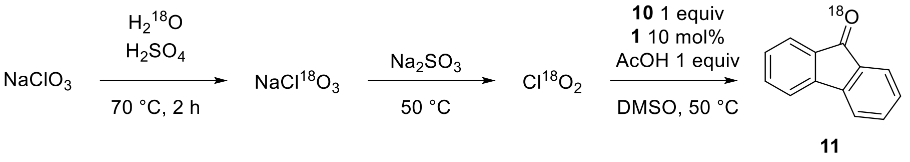

In one chamber of a COware reaction vessel, sodium chlorate (426 mg, 4 mmol) was dissolved in 1 mL of [^{18}O]-H_2O ([^{18}O], 97%). H_2SO_4 (166 µL, 0.78 equiv, 3.12 mmol) was added and the vessel sealed and stirred at 70 °C. After 2 hours, the mixture was frozen in a dry ice-acetone bath and solid sodium sulfite (227 mg, 0.45 equiv, 1.8 mmol) was added to the chamber containing the chlorate mixture. A solution of fluorene **10** (5 mg, 0.03 mmol), NHPI **1** (0.5 mg, 0.003 mmol) and acetic acid (1.7 µL, 0.03 mmol) in 0.5 mL DMSO was added to the second chamber. The vessel was sealed and both chambers heated to 50 °C with stirring. This resulted in the generation of yellow vapours from the chlorite chamber. The vessel was left to stir for 19 hours before cooling to room temperature and the DMSO mixture extracted with 3 mL 10% ethyl acetate in hexane and analysed by GCMS indicating the formation of [^{18}O]-fluorenone (*m/z* predicted 182.1, found 182.1) in 45% relative abundance in addition to [^{16}O]-fluorenone (*m/z* predicted 180.1, found 180.1).

If should be noted that sodium chlorite solutions generated by following the complete procedure from reference 5 were not suitable for use in our oxidation procedure due to the high pH of the solutions prepared and the low concentrations of chlorite generated.

Initially, we examined the oxidation of **10** using the chlorine dioxide generated from the sodium chlorate oxidation procedure outlined above in acetonitrile/water mixtures, however, this chlorine dioxide generation method also produced appreciable concentrations of elemental chlorine which resulted in ring chlorination of fluorenone product. The reaction medium was therefore changed to DMSO as described above to consume any reactive chlorine species.

The procedure was initially conducted with [^{16}O]-H_2O in the absence of fluorene **10**, NHPI **1** and acetic acid in order to obtain an approximate concentration of chlorine dioxide produced as determined by UV/vis spectroscopy. This allowed us to determine the reaction concentration in fluorene **10** (assuming 2 molar equivalents of chlorine dioxide were required for complete reaction). The procedure was then repeated with [^{16}O]-H_2O in the presence of fluorene **10**, NHPI **1** and acetic acid to establish a baseline [^{18}O]-content in the fluorenone product **11**. This was found to be 1% relative abundance in the absence of [^{18}O]-H_2O, showing the large increase in [^{18}O] under the labelling conditions.

COWare glassware used in the labelling experiments.

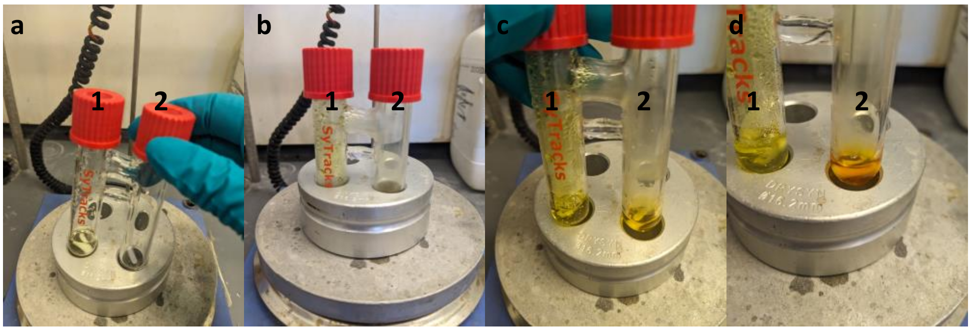

a) Chamber 1: $NaClO_3$, $H_2{}^{18}O$, H_2SO_4.

b) Chamber 1: $NaClO_3$, $H_2{}^{18}O$, H_2SO_4 heated to 70 °C for 2 h to generate $NaCl^{18}O_3$.

c) Chamber 1: $NaCl^{18}O_3$, Na_2SO_3, $H_2{}^{18}O$, H_2SO_4. Chamber 2: Fluorene **10**, NHPI **1** 10 mol%, AcOH 1 equiv, DMSO, 50 °C. Chlorine dioxide can be seen transferring from Chamber 1 to Chamber 2.

d) COware reaction upon completion of transformation.

GC trace and mass spectrum of fluorenone **11** (12.3 min) using unlabelled chlorine dioxide.

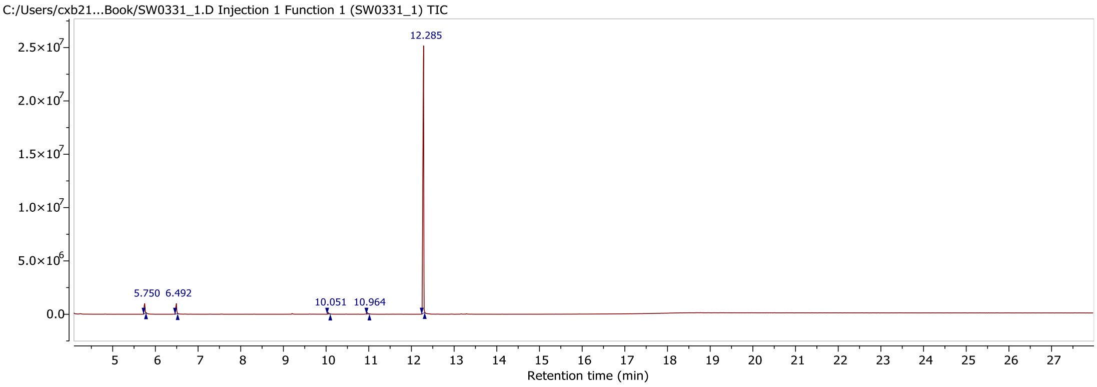

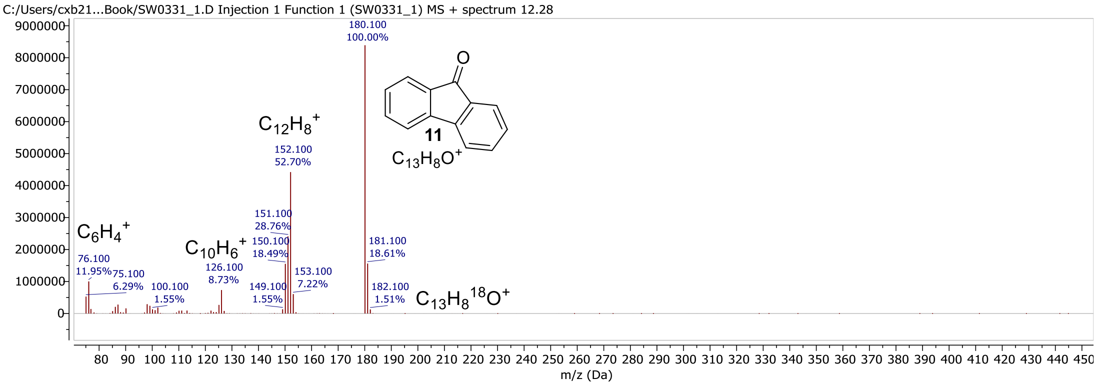

GC trace and mass spectrum of fluorenone **11** (12.3 min) using ^{18}O labelled chlorine dioxide.

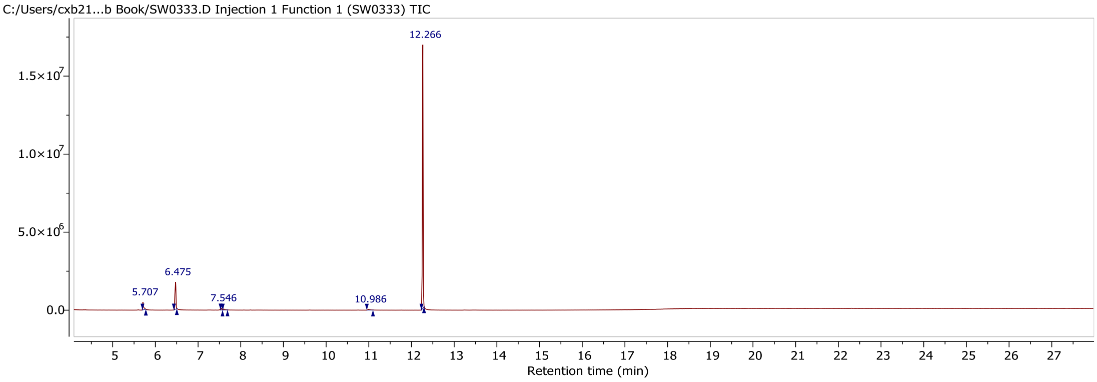

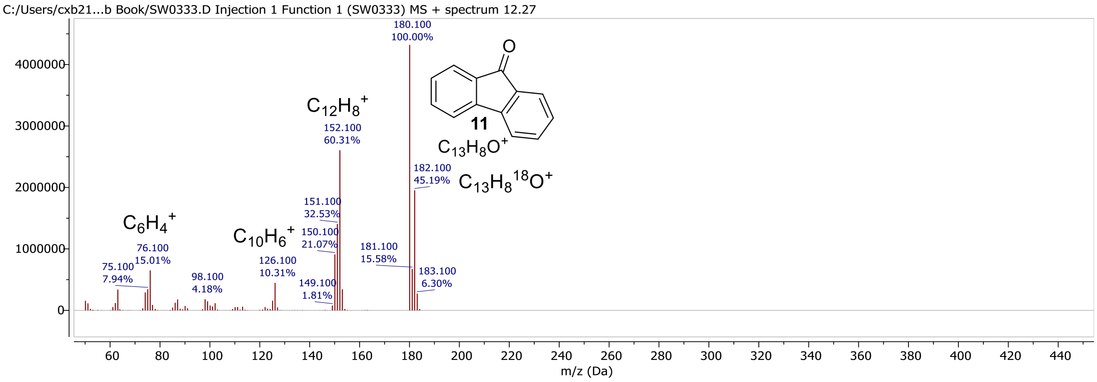

Inhibition of oxidation of fluorene 10 by DMSO

Reactions carried out according to general HPLC monitoring procedure. Fluorene **10** 0.5 mmol, NHPI **1** 10 mol%, $NaClO_2$ 1.5 equiv, AcOH 1 equiv, MeCN:H_2O (2:1), 50 °C in the presence of 0, 2 and 10 equiv DMSO.

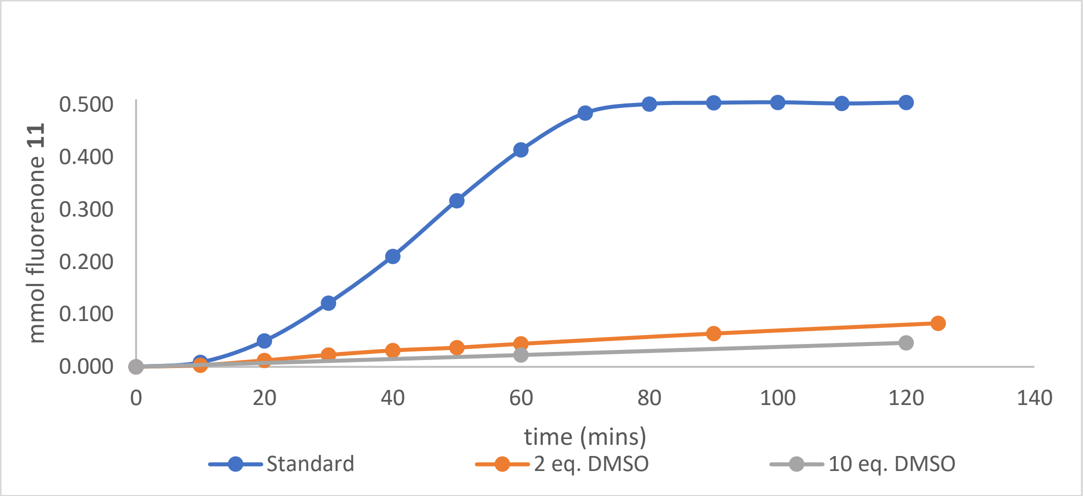

Generation of standard solutions of chlorine dioxide

Solutions of chlorine dioxide were generated following a literature procedure.[6] Aqueous sodium chlorite was reacted with an aqueous solution of sodium peroxydisulfate, and the evolved gas was passed through a column containing ascarite and collected in a flask containing cold acetonitrile. The chlorine dioxide concentration was determined *via* iodometric titration. A calibration curve was obtained by measuring the absorbance of different concentration solutions at 360 nm.

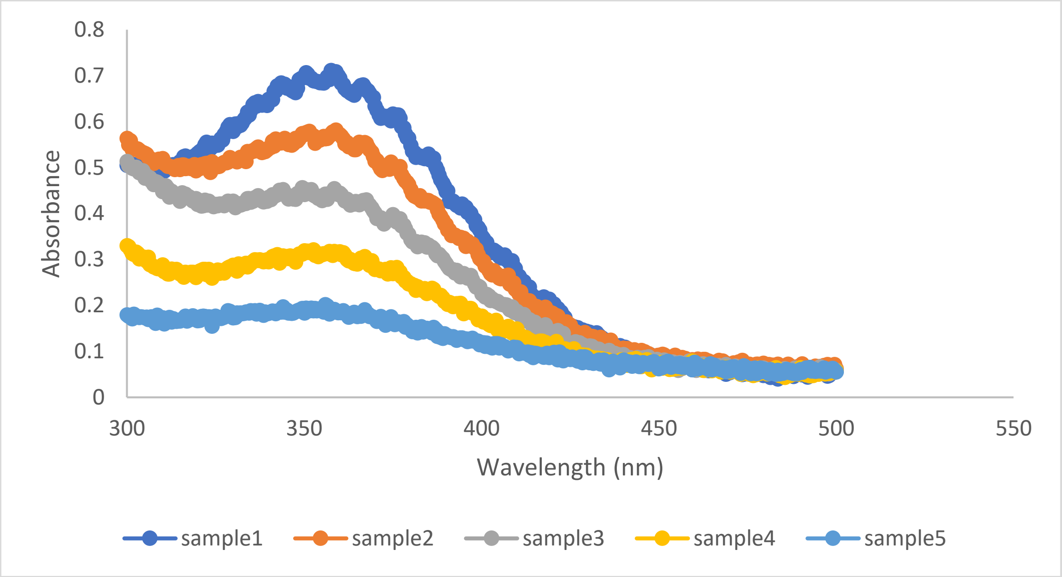

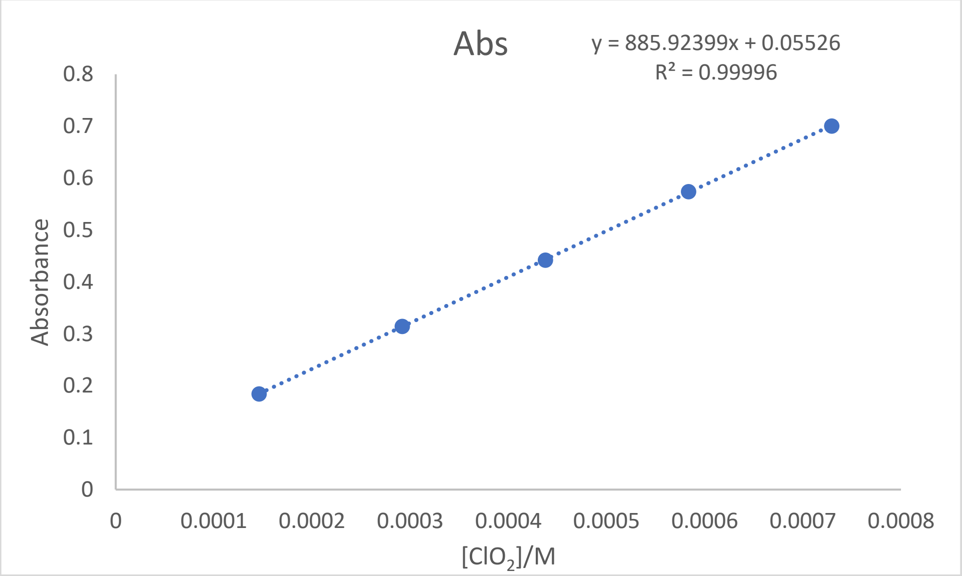

UV/vis Study

Standard solutions of fluorene **10** (0.0169 M), NHPI **1** (0.0067 M), acetic acid (0.0672 M), sodium chlorite (0.1008 M) and sodium hypochlorite (0.0067 M) in 2:1 MeCN/H_2O were prepared. Aliquots were taken of each solution as required, added to a quartz cuvette and diluted with 2:1 MeCN/H_2O to give the given final concentration (total volume 1.4 mL). Spectra were recorded on a Varian Cary 50 UV-Vis spectrophotometer. The temperature was controlled using a Cary Single Cell Peltier accessory.

NHPI 1 + NaClO

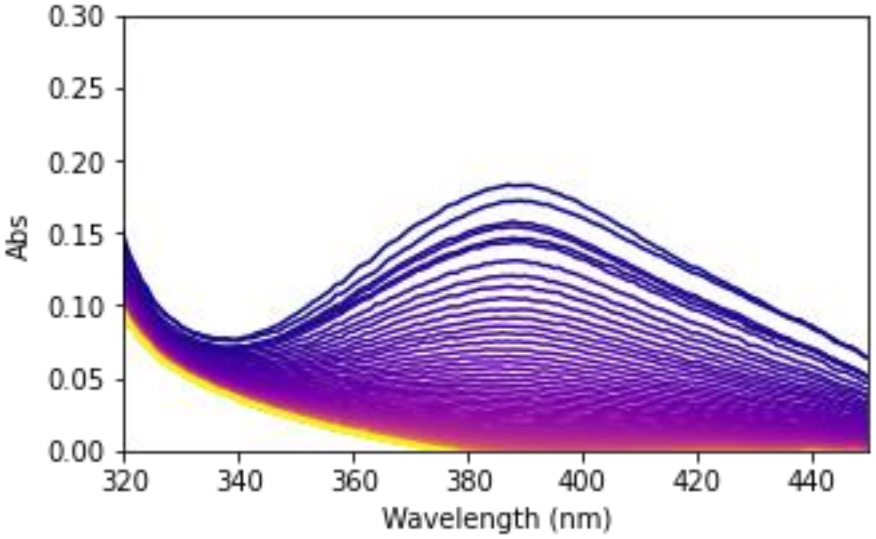

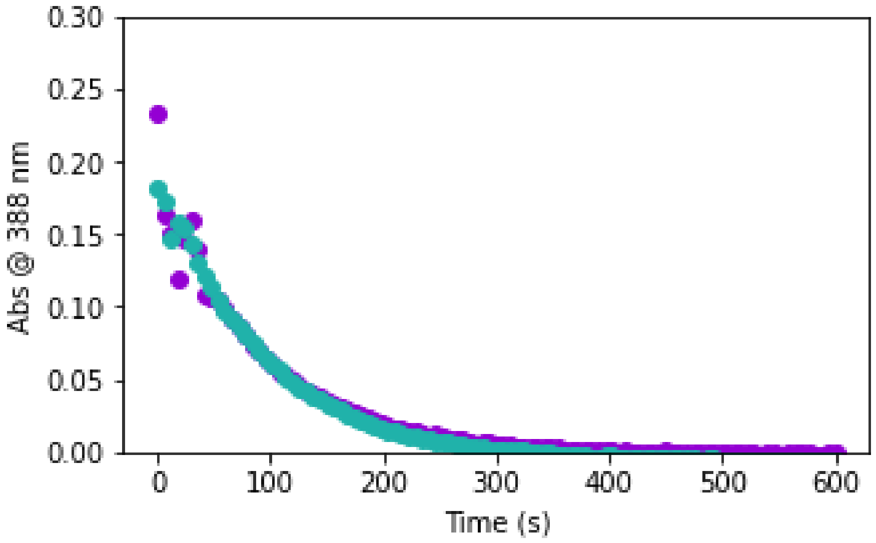

UV/vis spectra of a cuvette charged with 0.1 mL NHPI solution, 0.1 mL NaClO solution and 1.2 mL solvent mixture (2:1 MeCN/H_2O).

Fluorene 10 + NHPI 1 + HOAc + $NaClO_2$

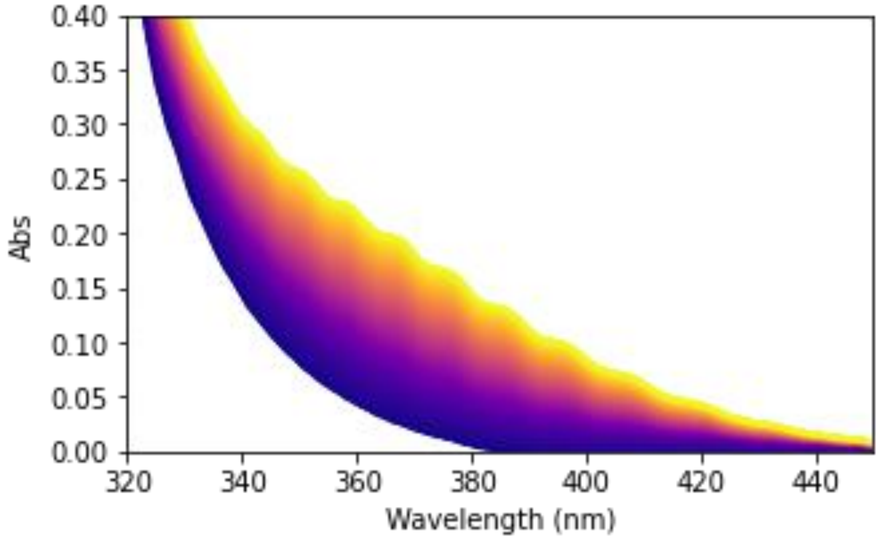

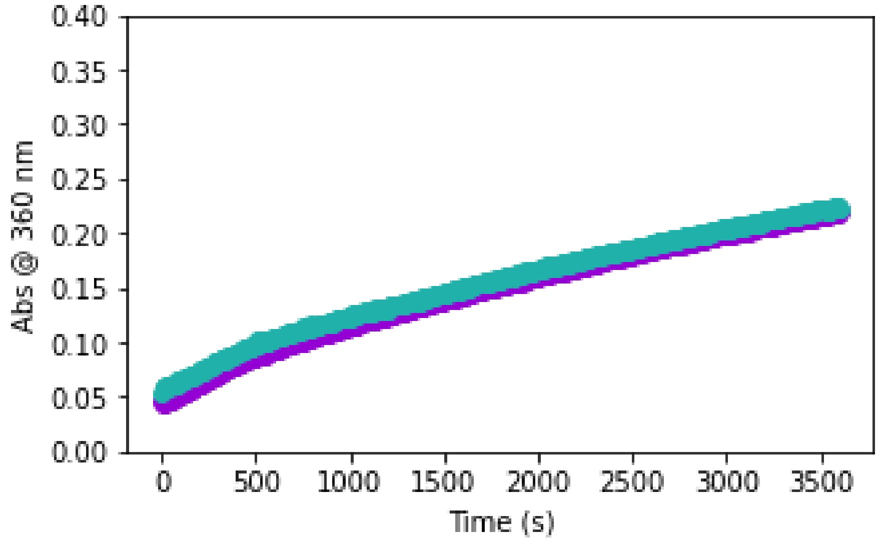

UV/vis spectra of a cuvette charged with 0.4 mL fluorene **10** solution, 0.1 mL NHPI **1** solution, 0.1 mL acetic acid solution, 0.1 mL $NaClO_2$ solution and 0.7 mL solvent mixture (2:1 $MeCN/H_2O$).

Fluorene 10 + HOAc + $NaClO_2$

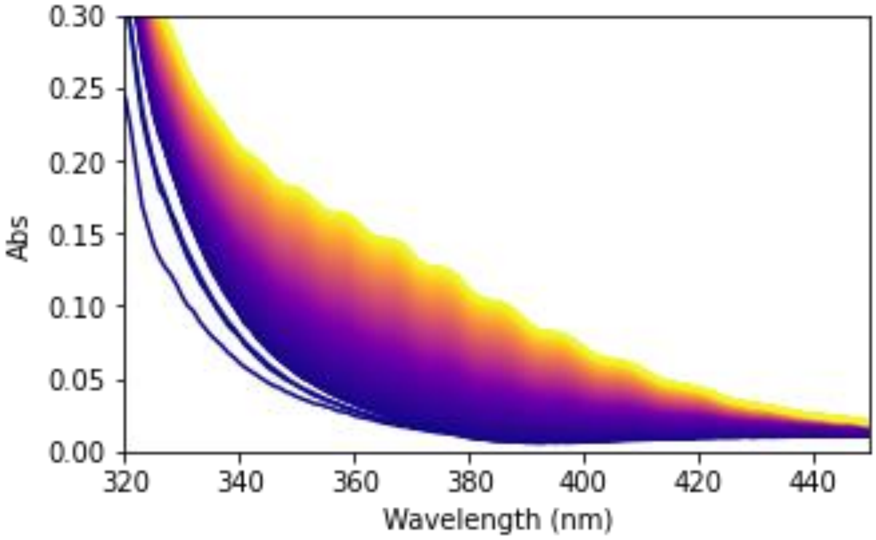

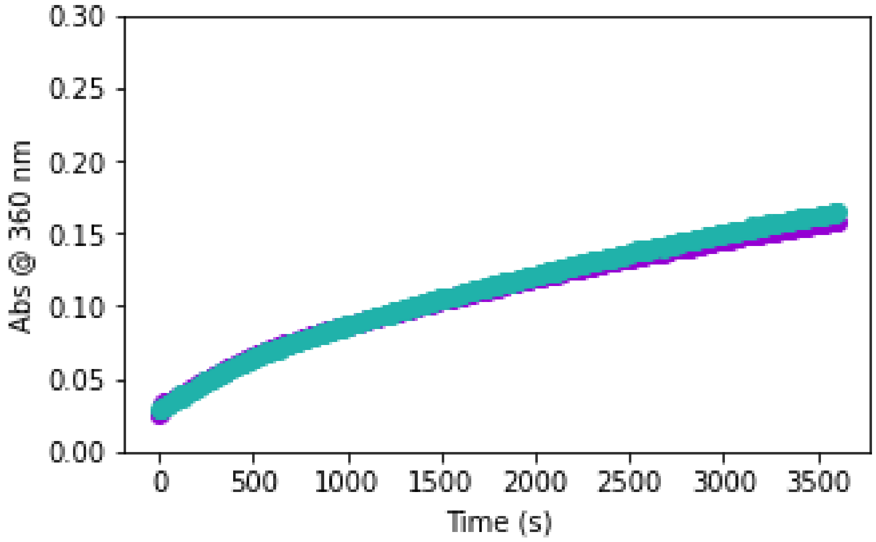

UV/vis spectra of a cuvette charged with 0.4 mL fluorene **10** solution, 0.1 mL acetic acid solution, 0.1 mL $NaClO_2$ solution and 0.8 mL solvent mixture (2:1 $MeCN/H_2O$).

Entry	Conditions	$[ClO_2]_{max}$/M
1	**10** 1 equiv, **1** 10 mol%, AcOH 1 equiv	1.88×10^{-4} (10%)
2	**10** 1 equiv, AcOH 1 equiv	1.21×10^{-4} (7%)

EPR Experiments

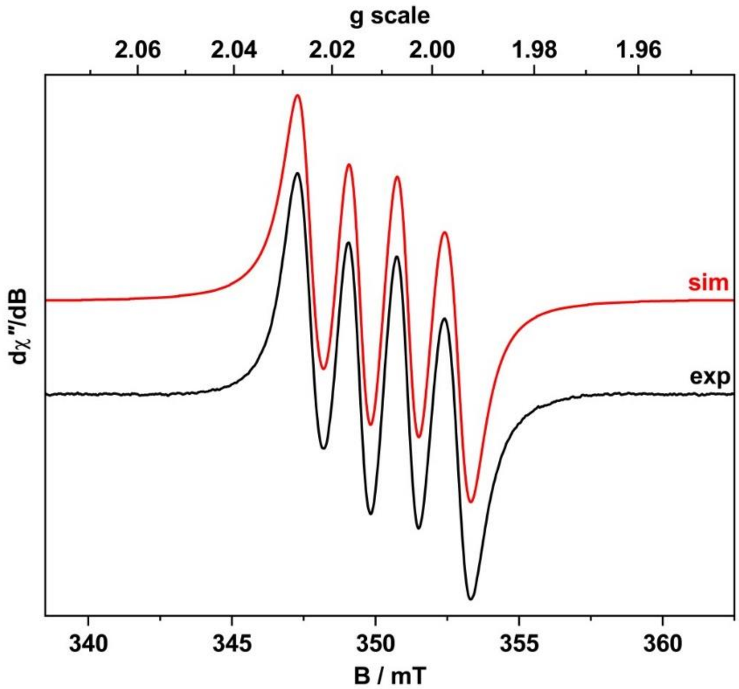

X-band EPR spectrum of the chlorine dioxide radical generated by mixing 2 vol. acetonitrile solution of phthalan **22** (1 equiv, 0.125 M), NHPI **1** (10 mol%, 0.0125 M) and acetic acid (1 equiv, 0.125 M) with 1 vol. aqueous sodium chlorite (0.375 M). Experimental data represented by the black line; simulation is depicted by the red trace; g_{iso} = 2.0095, $A\{^{35,37}Cl\}$ = 16.4 × 10^{-4} cm^{-1} (where 1 Gauss ≈ 10^{-4} cm^{-1}).

Comparison of the X-band EPR spectrum of the mixture of $ClO_2^{\bullet}$ (4.0×10^{-4} M) with 20 equiv of NHPI **1** in 2:1 MeCN/H_2O.

Experimental conditions: frequency, 9.8654 GHz; power, 0.63 mW; modulation, 0.2 mT.

Spectra have been enlarged to improve visibility. Drop lines indicate the emergence of the PINO$^{\bullet}$ radical **2** with a concomitant decrease in the four-line signal for the $ClO_2^{\bullet}$ radical.

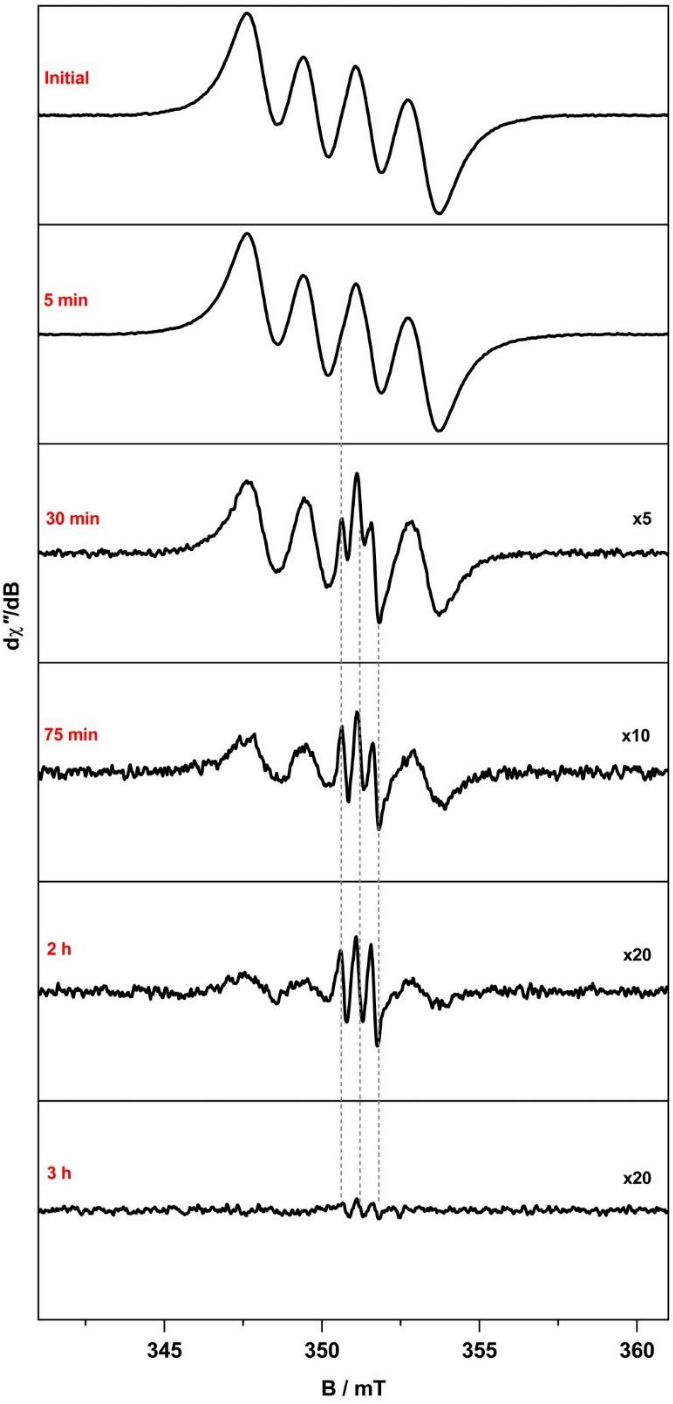

Comparison of the X-band EPR spectrum of a mixture of $ClO_2^{\bullet}$ (4.0×10^{-4} M) with 20 equiv of K-salt of PINO anion **20** in 2:1 MeCN/H_2O.

Experimental conditions: frequency, 9.8654 GHz; power, 0.63 mW; modulation, 0.2 mT.

Spectra have been enlarged to improve visibility. Drop lines indicate the emergence of the PINO$^{\bullet}$ radical **2** with a concomitant decrease in the four-line signal for the $ClO_2^{\bullet}$ radical.

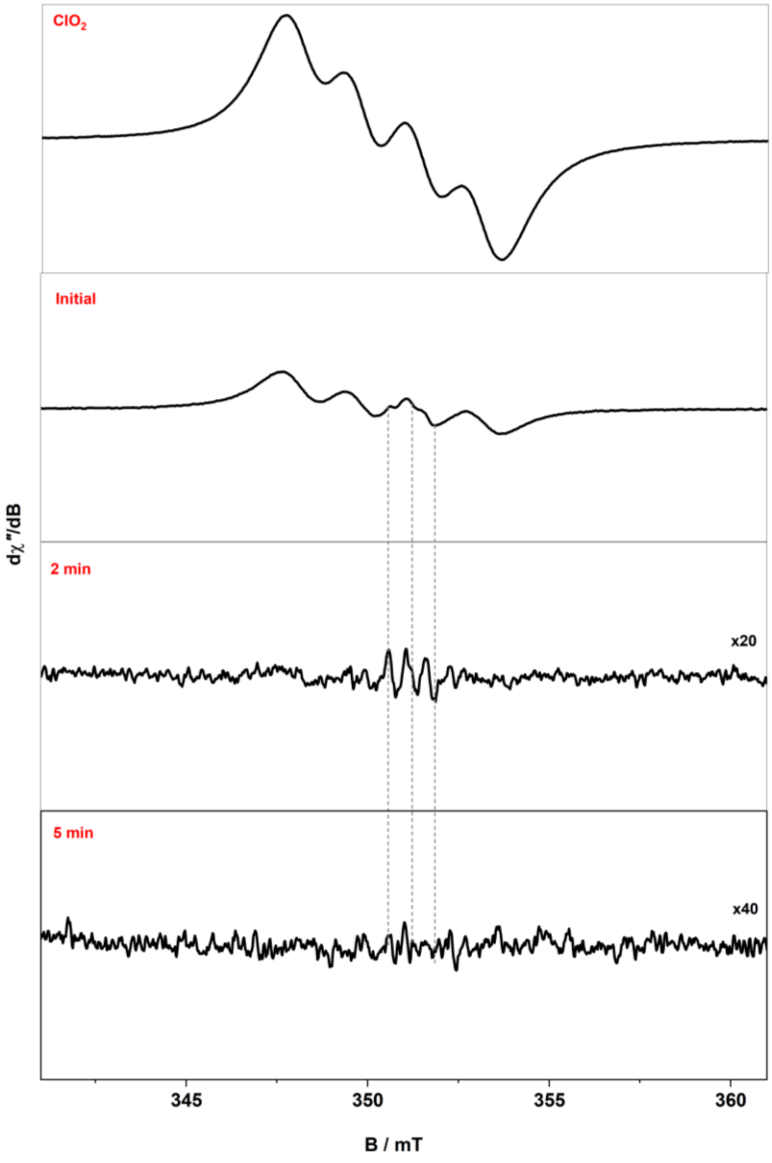

X-band EPR spectrum of the PINO$^{\bullet}$ radical **2** generated by addition of 1 mL of 0.008 M NaClO solution to 0.008 M NHPI in 2:1 MeCN/H_2O (experimental conditions: frequency, 9.8647 GHz; power, 0.63 mW; modulation, 0.05 mT). Experimental data represented by the black line; simulation is depicted by the red trace: g_{iso} = 2.00734; $A_{iso}\{^{14}N\}$ = 4.6 × 10^{-4} cm^{-1}.

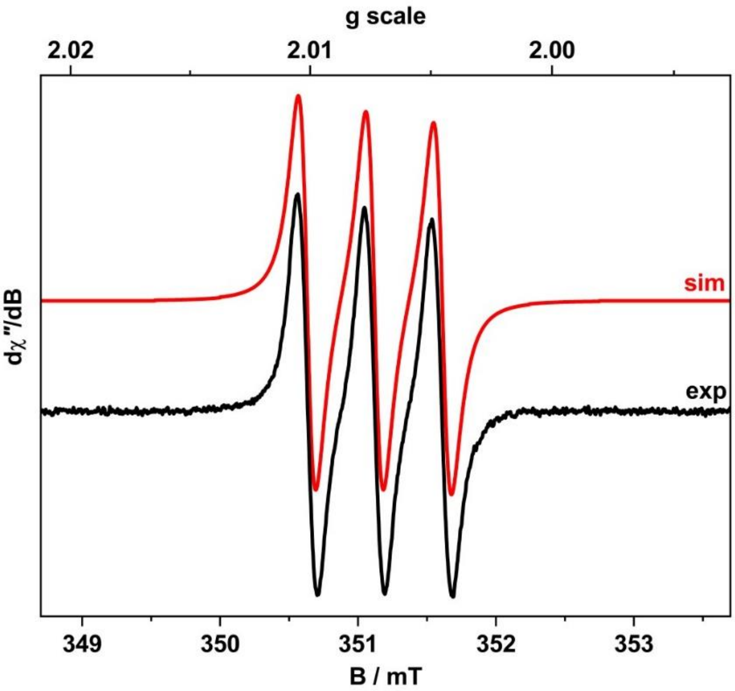

Radical Trapping Experiments

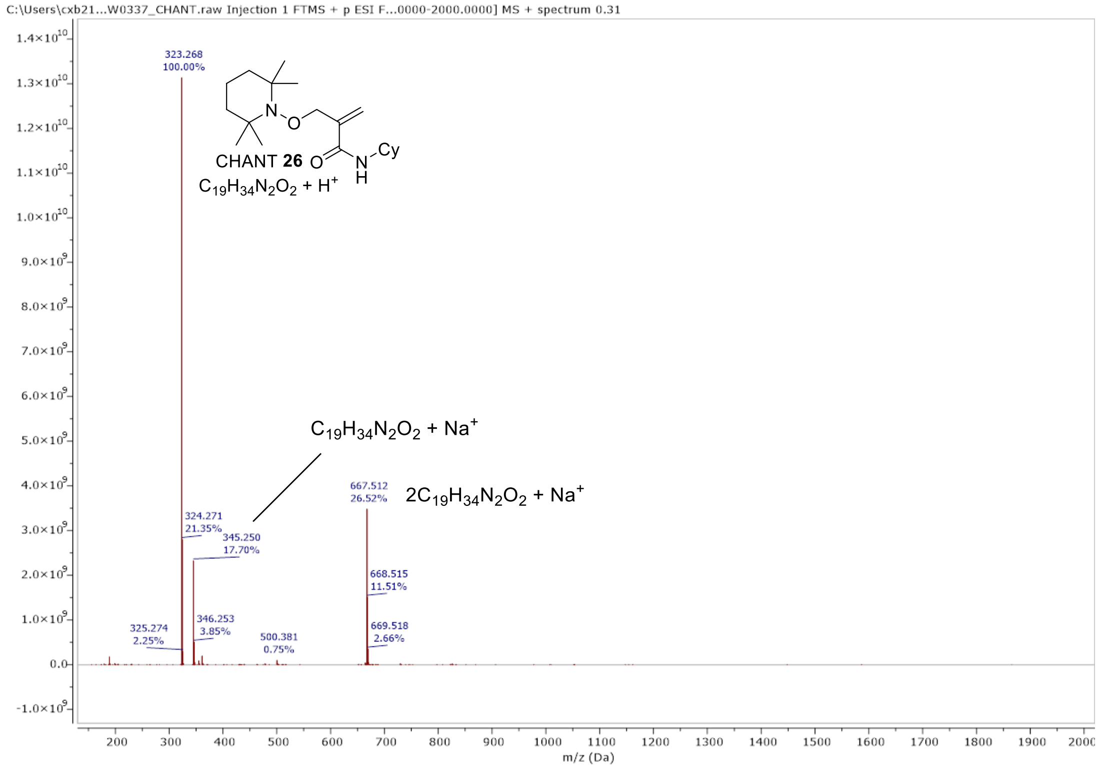

Copy of mass spectrum of CHANT **26**.

Reaction in presence of 5 mol% CHANT – Trapping After Induction Period

Fluorene **10** (20 mg, 0.12 mmol), NHPI **1** (0.1 equiv, 2 mg, 0.012 mmol) and acetic acid (1 equiv, 7 µL, 0.12 mmol) were dissolved in 0.96 mL acetonitrile. Aqueous sodium chlorite (0.48 mL) (80% technical grade, 1.5 equiv, 20 mg, 0.18 mmol) was added and the mixture stirred at 50 °C for 30 minutes. CHANT **26** (0.05 equiv, 2 mg, 0.006 mmol) in 0.48 mL acetonitrile was added to the reaction mixture and stirring was continued at 50 °C. A 10 µL sample was taken after 15 minutes, diluted in 1 mL acetonitrile and analysed by HRMS. Mass ion 351.1308 was detected, consistent with the sodium adduct of adduct **27**. No other expected radical adducts from the reaction mixture were detected.

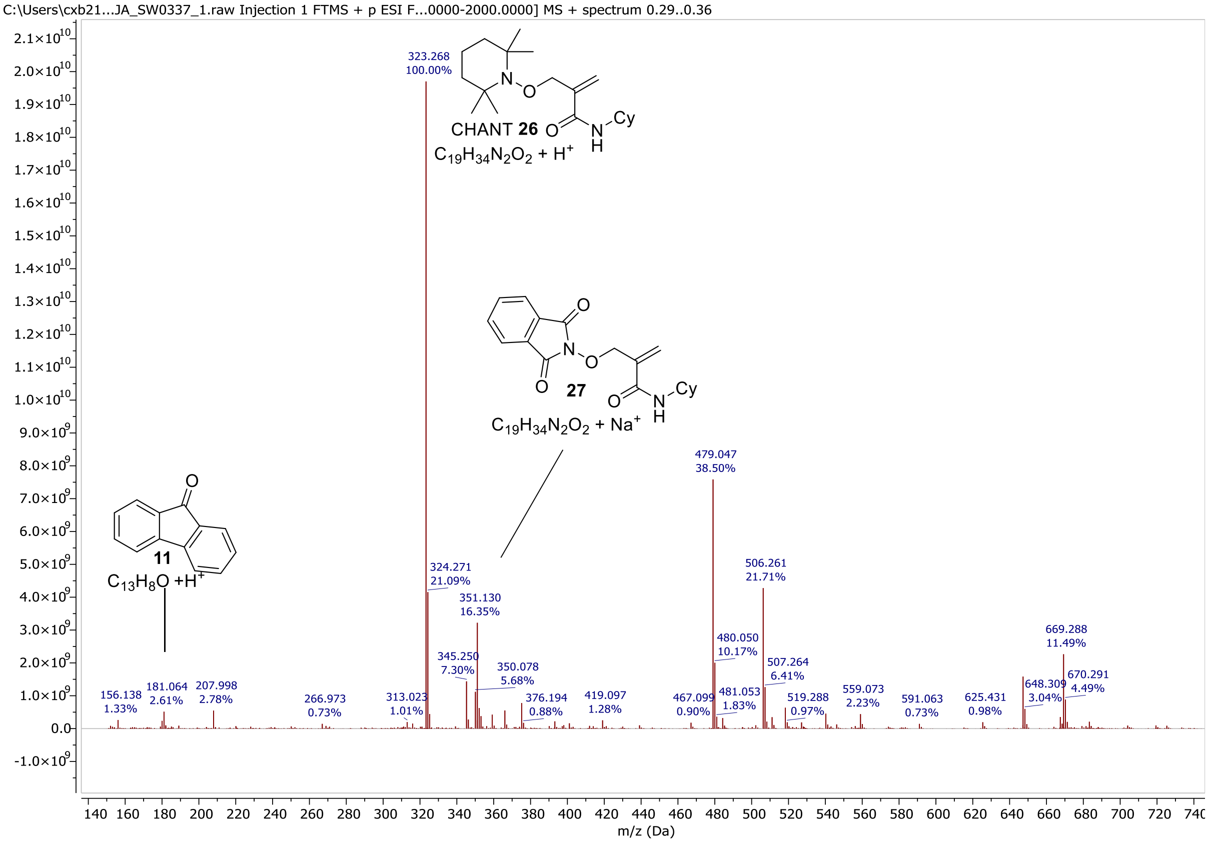

Copy of mass spectrum from reaction sample after 5 minutes.

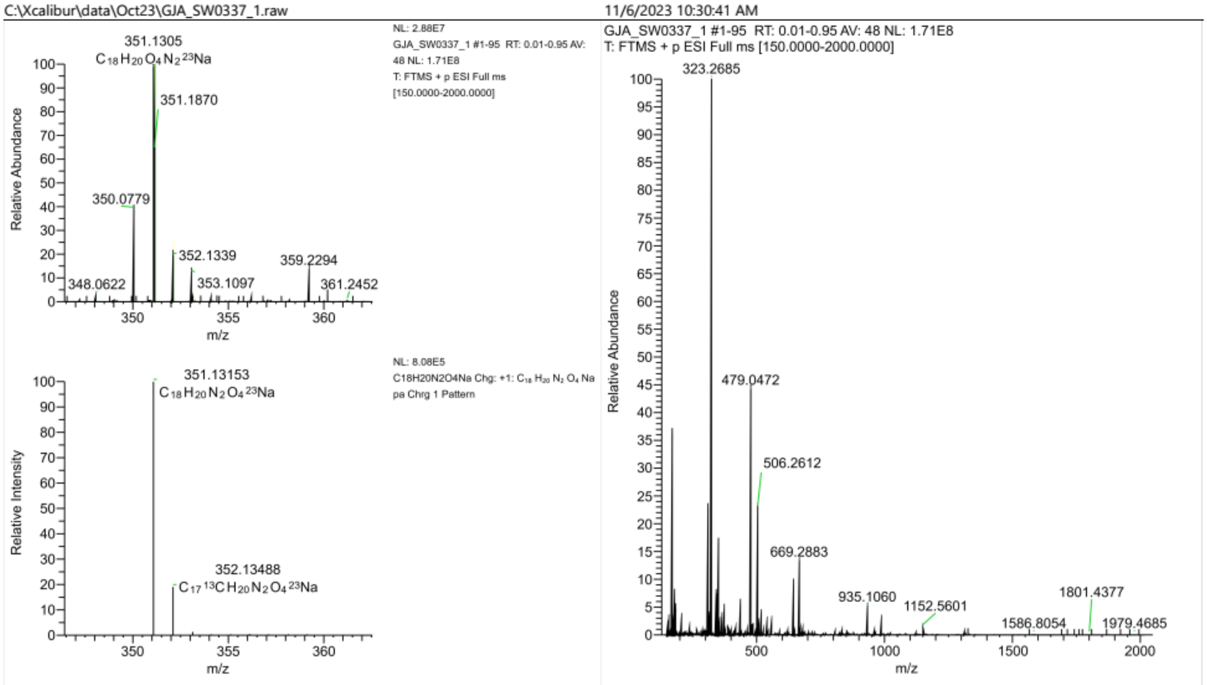

Peak Mass	Display For...	S Fit	RDB	Delta [ppm]	Theo. mass	Rank	Combined S...	# Matched I...	# Missed Iso.	MS Cov. [%]	Pattern Cov....	MSMS Matc...
351.1305	$C_{16}H_{15}O_2N_8$	65.3289260374687	13.50	-2.13	351.13125	1	86.55	4	0	87.73	100	(Collection)
351.1305	$C_{18}H_{20}O_4N_2{}^{23}Na$	38.6493660013642	9.50	-2.92	351.13153	2	85.05	4	0	87.62	99.8	(Collection)

Mass matching for adduct **27** + Na^+ (5 minutes).

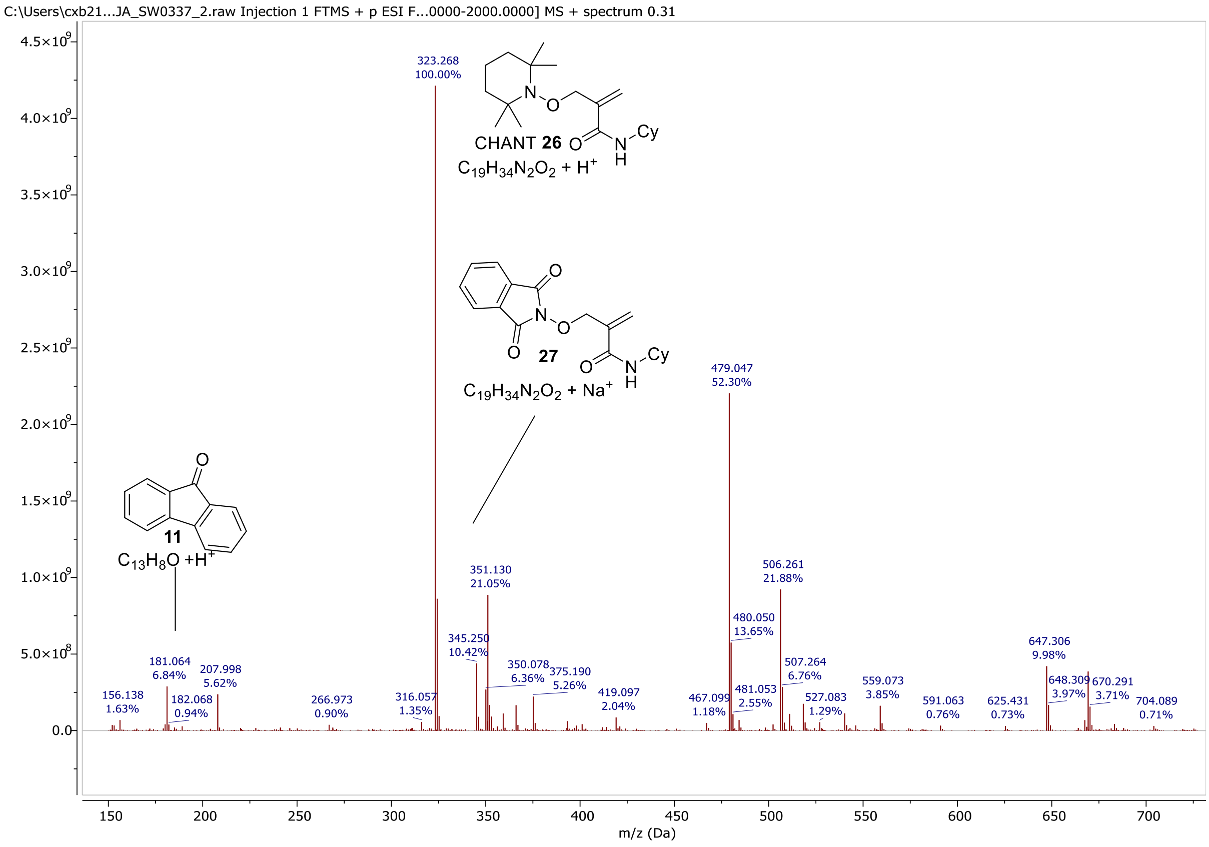

Copy of mass spectrum from reaction sample after 15 minutes.

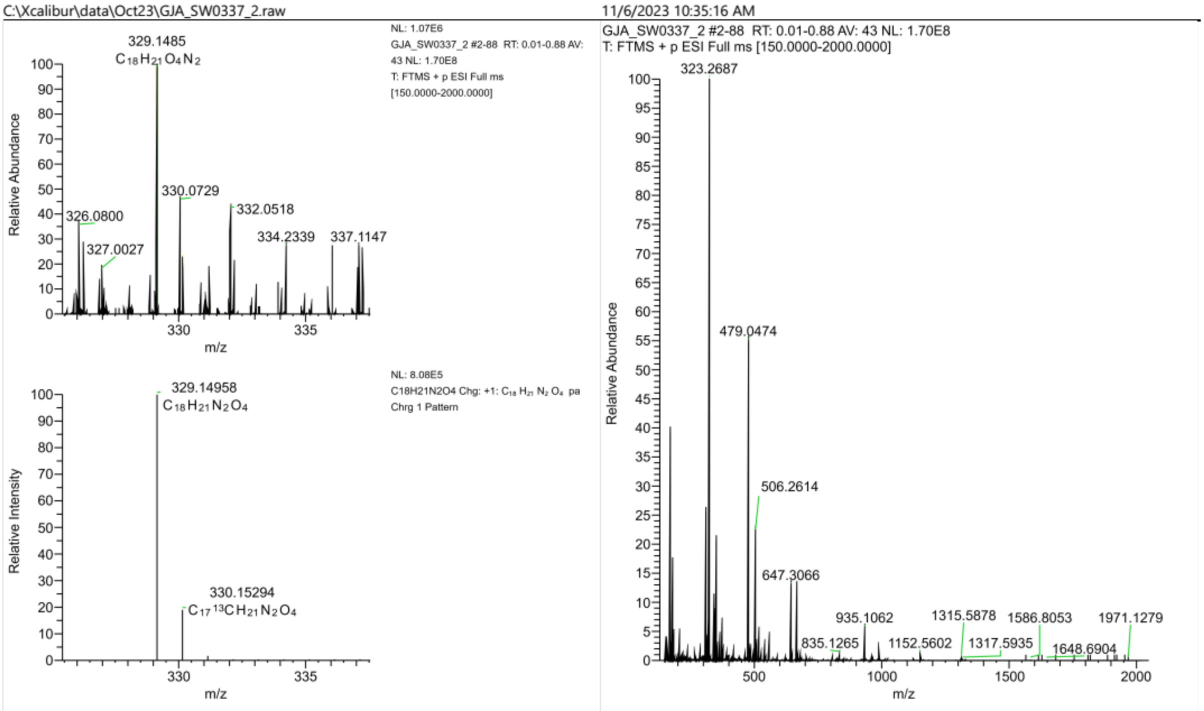

Peak Mass	Display For...	S Fit	RDB	Delta [ppm]	Theo. mass	Rank	Combined S...	# Matched I...	# Missed Iso.	MS Cov. [%]	Pattern Cov....	MSMS Matc...
329.1485	$C_{14}H_{17}O_2N_8$	8.86333751705699	10.50	4.96	329.14690	2	44.09	3	0	46.04	85.65	(Collection)
329.1485	$C_{18}H_{21}O_4N_2$	34.1598628679167	9.50	-3.20	329.14958	1	54.54	3	0	55.67	98.08	(Collection)

Mass matching for adduct **27** + H^+ (15 minutes).

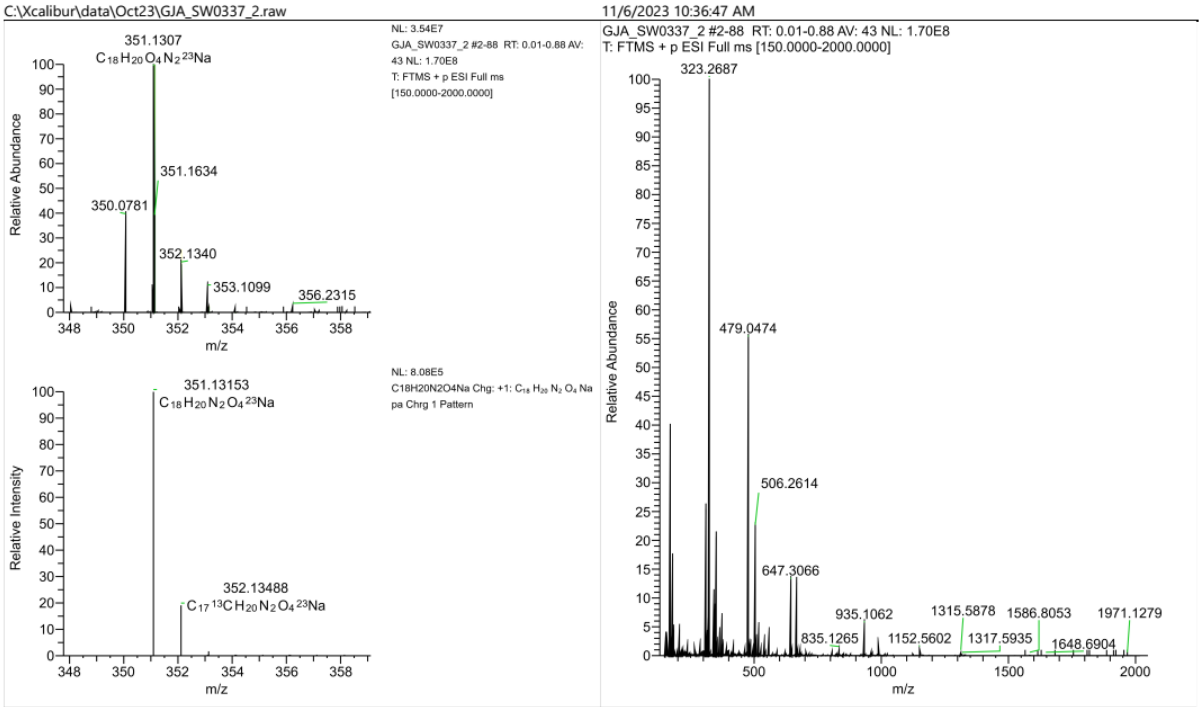

Peak Mass	Display For...	S Fit	RDB	Delta [ppm]	Theo. mass	Rank	Combined S...	# Matched I...	# Missed Iso.	MS Cov. [%]	Pattern Cov....	MSMS Matc...
351.1307	$C_{16}H_{15}O_2N_8$	56.493851176789	13.50	-1.53	351.13125	1	87.56	4	0	89.28	100	(Collection)
351.1307	$C_{18}H_{20}O_4N_2{}^{23}Na$	52.3002460006003	9.50	-2.33	351.13153	2	87.34	4	0	89.28	100	(Collection)

Mass matching for adduct **27** + Na^+ (15 minutes).

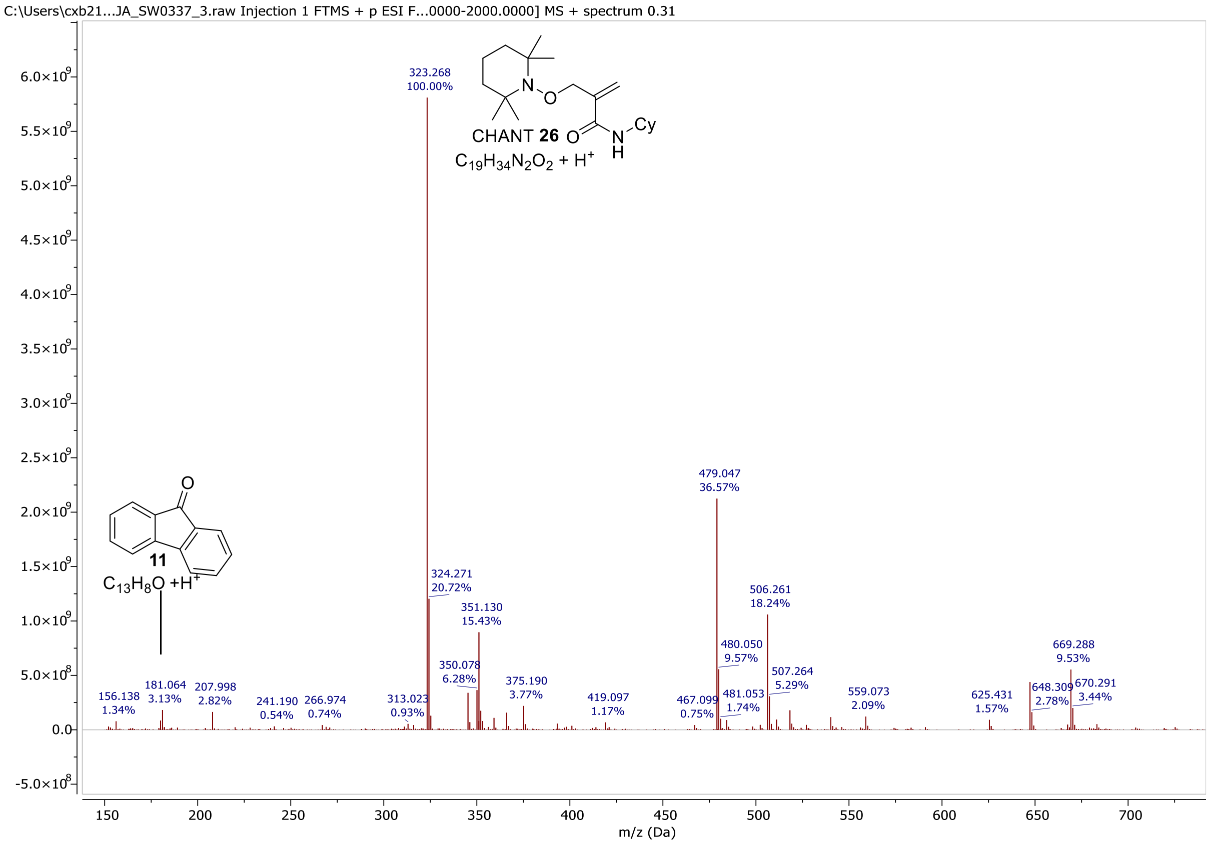

Copy of mass spectrum from reaction sample after 30 minutes.

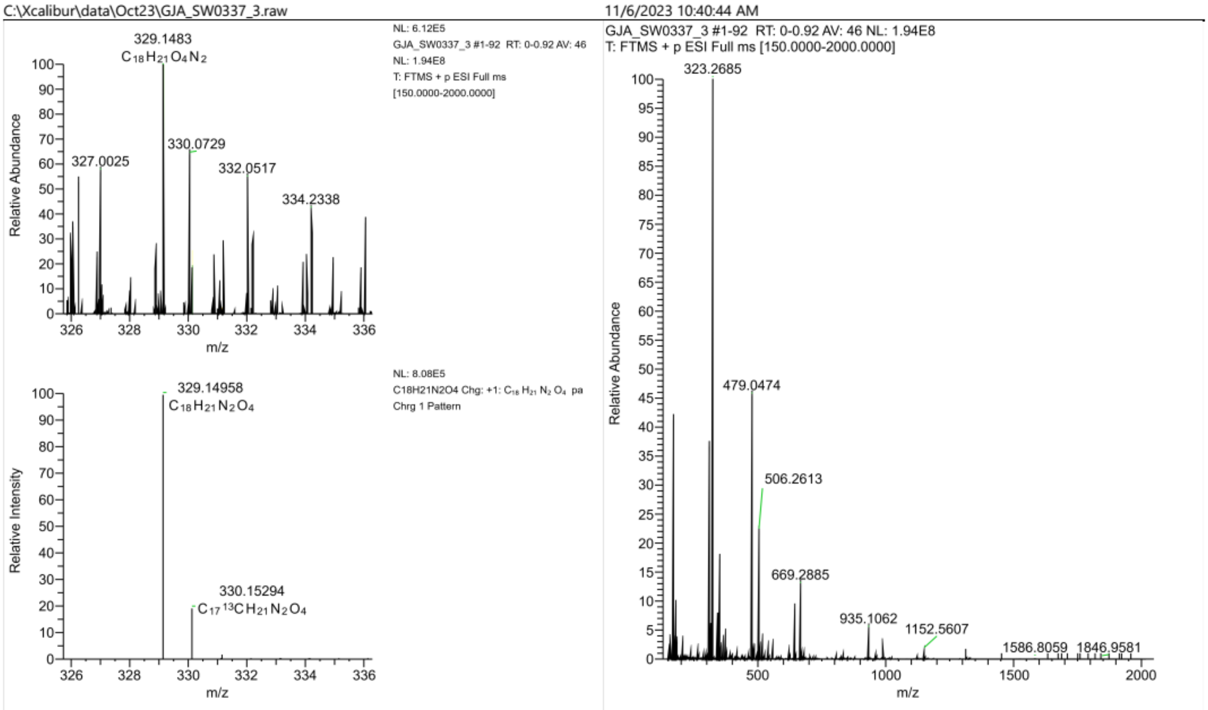

Peak Mass	Display For...	S Fit	RDB	Delta [ppm]	Theo. mass	Rank	Combined S...	# Matched I...	# Missed Iso.	MS Cov. [%]	Pattern Cov....	MSMS Matc...
329.1483	$C_{14}H_{17}O_2N_8$	11.34922692 62814	10.50	4.23	329.14690	2	38.73	3	0	40.25	85.72	(Collection)
329.1483	$C_{18}H_{21}O_4N_2$	29.18930697 08245	9.50	-3.93	329.14958	1	46.13	3	0	47.07	98.08	(Collection)

Mass matching for adduct **27** + H^+ (30 minutes).

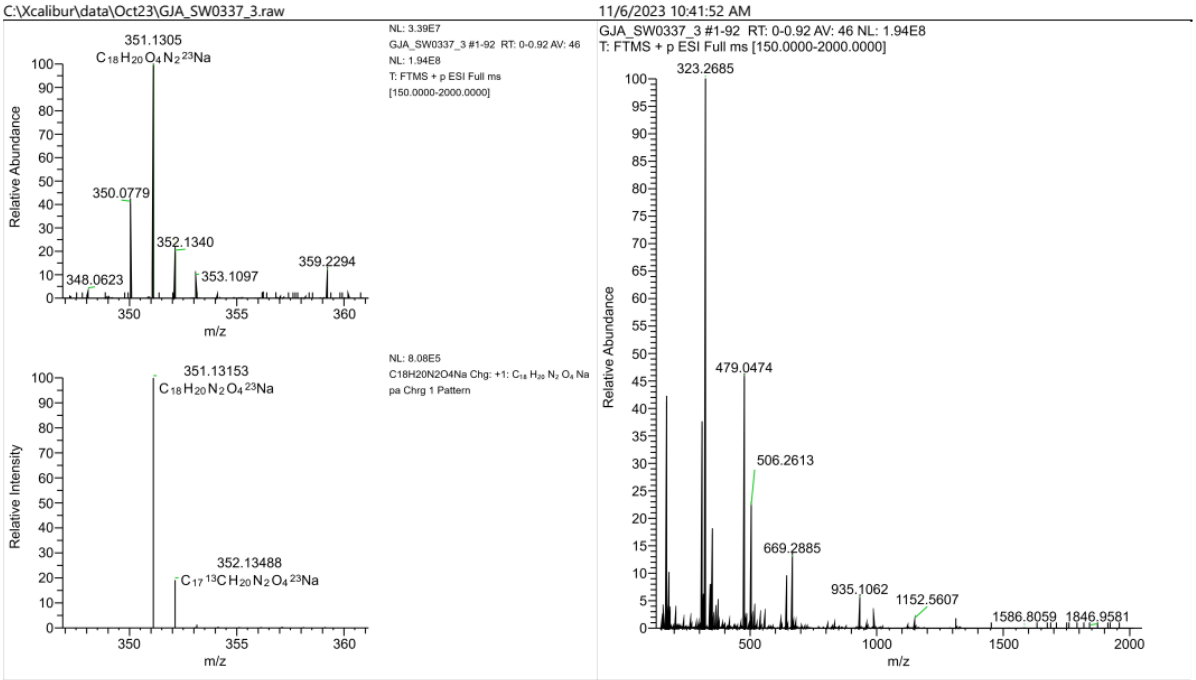

Peak Mass	Display For...	S Fit	RDB	Delta [ppm]	Theo. mass	Rank	Combined S...	# Matched I...	# Missed Iso.	MS Cov. [%]	Pattern Cov....	MSMS Matc...
351.1305	$C_{16}H_{15}O_2N_8$	57.28749726 11479	13.50	-2.07	351.13125	1	88.87	4	0	90.62	100	(Collection)
351.1305	$C_{18}H_{20}O_4N_2{}^{23}Na$	39.44329486 49846	9.50	-2.87	351.13153	2	87.85	4	0	90.54	99.8	(Collection)

Mass matching for adduct **27** + Na^+ (30 minutes).

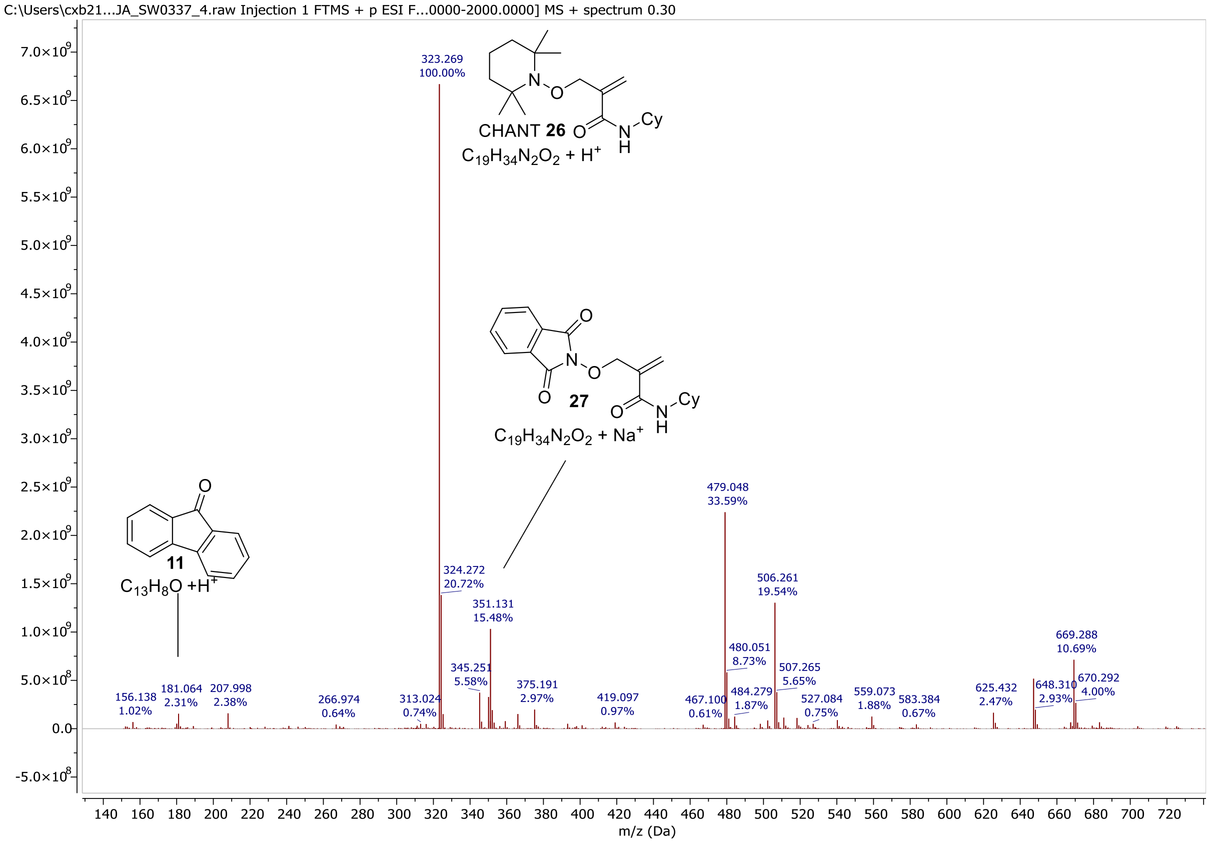

Copy of mass spectrum from reaction sample after 1 hour.

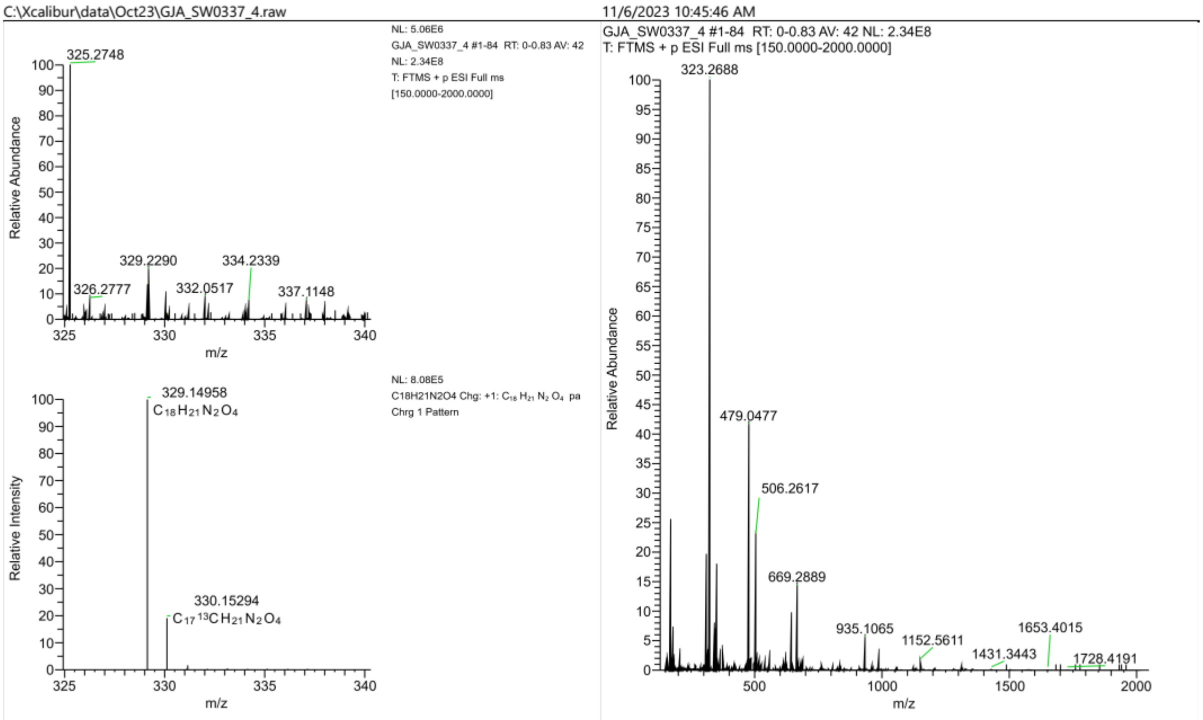

Peak Mass	Display For...	S Fit	RDB	Delta [ppm]	Theo. mass	Rank	Combined S...	# Matched I...	# Missed Iso.	MS Cov. [%]	Pattern Cov....	MSMS Matc...
329.1484	$C_{14}H_{17}O_2N_8$	10.25429877 59043	10.50	4.56	329.14690	2	21.7	3	0	22.33	85.69	(Collection)
329.1484	$C_{18}H_{21}O_4N_2$	25.33080907 01141	9.50	-3.60	329.14958	1	25.58	3	0	25.6	98.07	(Collection)

Mass matching for adduct **27** + H^+ (1 hour).

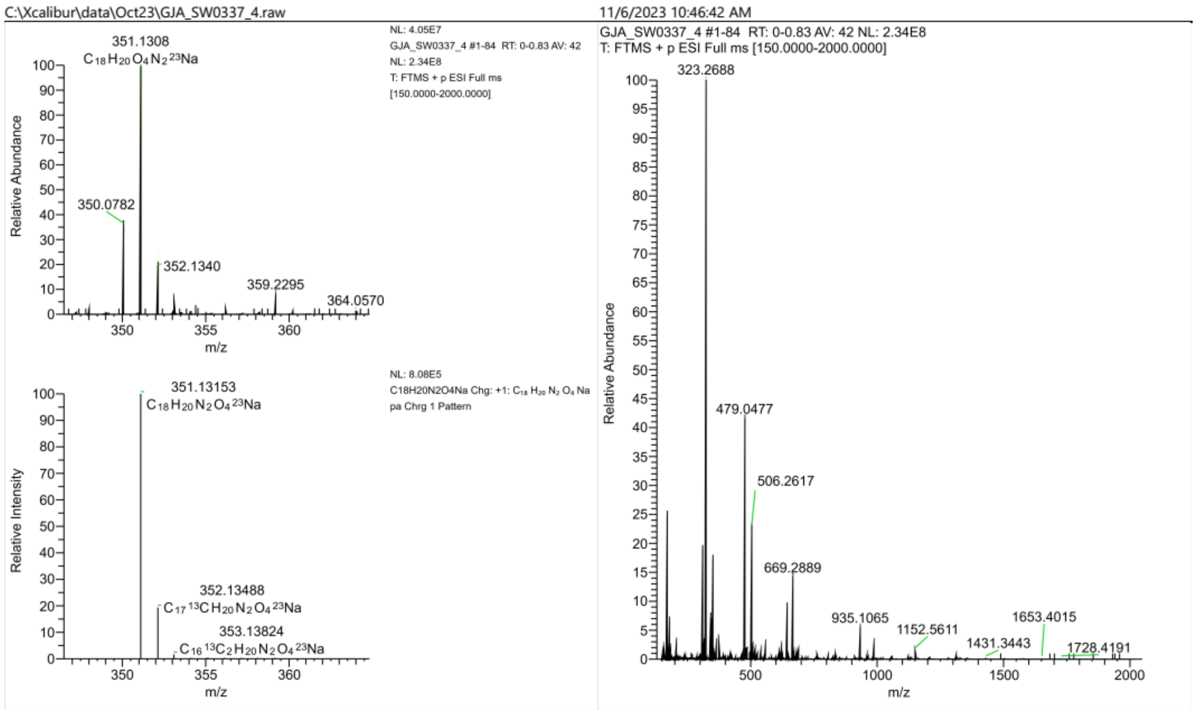

Peak Mass	Display For...	S Fit	RDB	Delta [ppm]	Theo. mass	Rank	Combined S...	# Matched I...	# Missed Iso.	MS Cov. [%]	Pattern Cov....	MSMS Matc...
351.1308	$C_{16}H_{15}O_2N_8$	56.54546579 27383	13.50	-1.31	351.13125	1	88.96	4	0	90.76	100	(Collection)
351.1308	$C_{18}H_{20}O_4N_2{}^{23}Na$	43.28784135 74526	9.50	-2.11	351.13153	2	88.15	4	0	90.64	99.8	(Collection)

Mass matching for adduct **27** + Na^+ (1 hour).

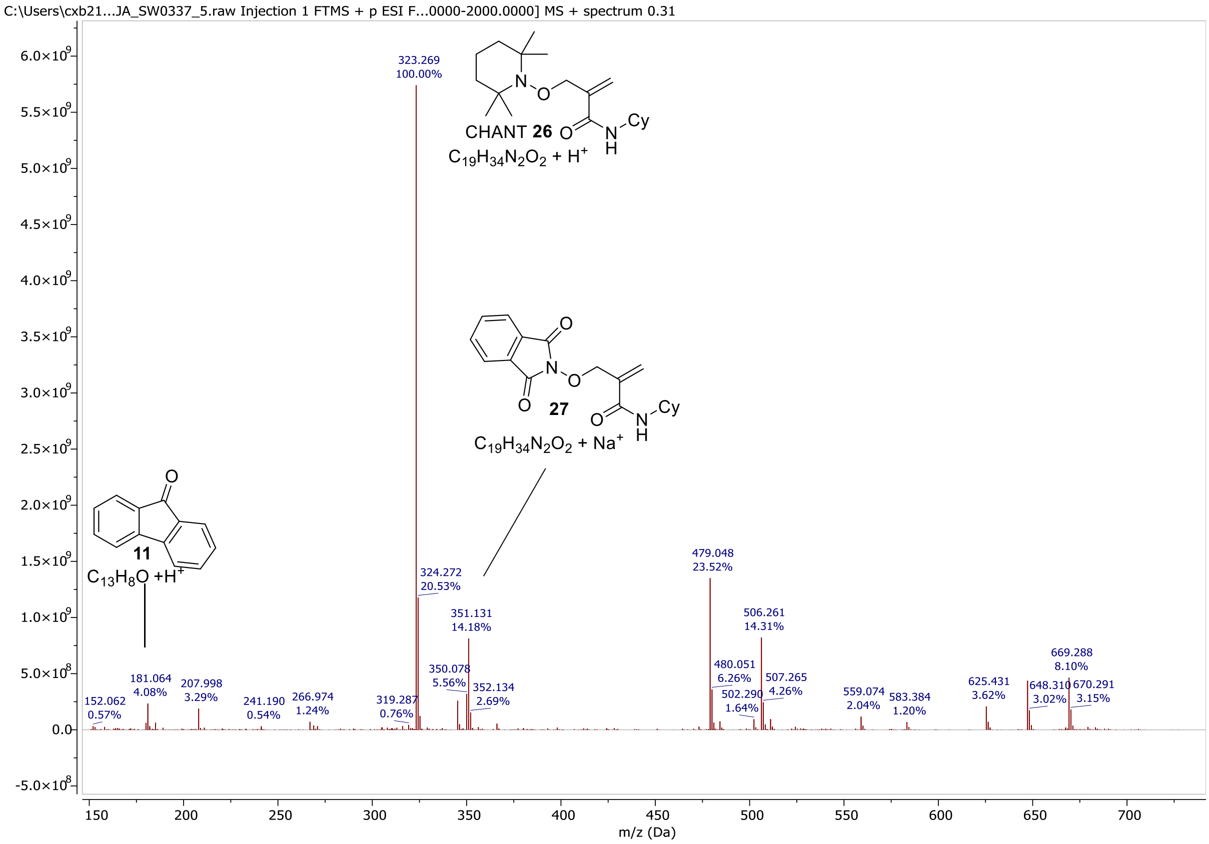

Copy of mass spectrum from reaction sample after 19 hours.

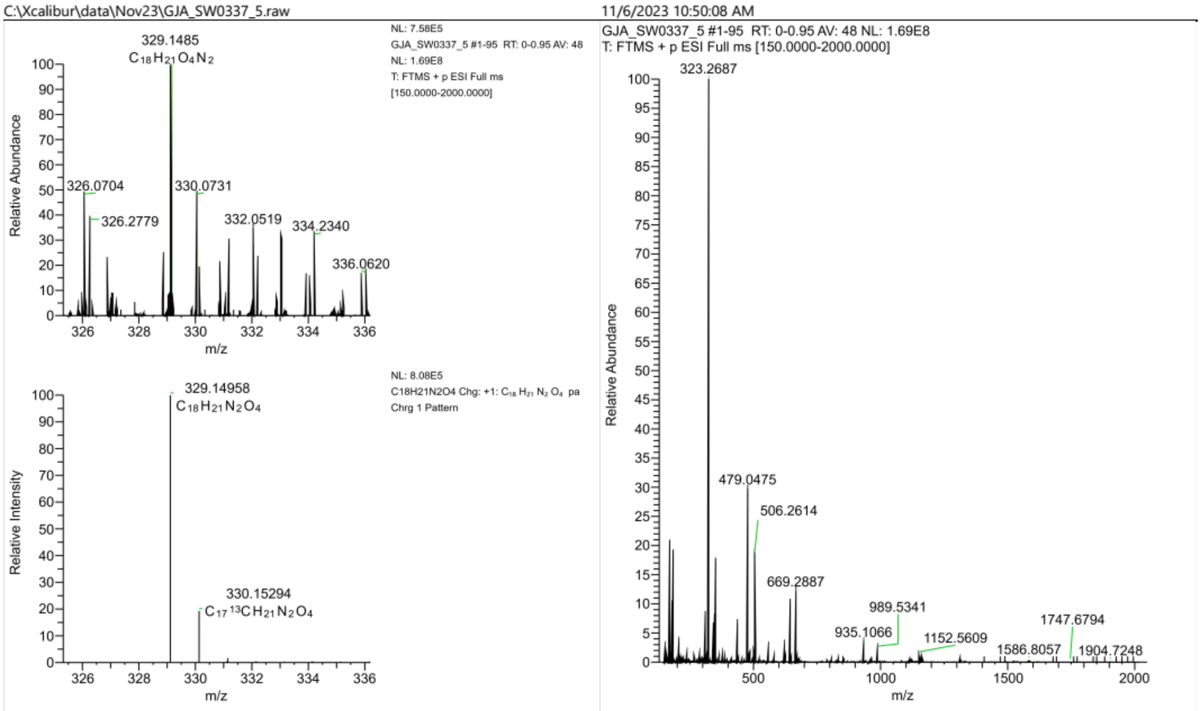

Peak Mass	Display For...	S Fit	RDB	Delta [ppm]	Theo. mass	Rank	Combined S...	# Matched I...	# Missed Iso.	MS Cov. [%]	Pattern Cov....	MSMS Matc...
329.1485	$C_{14}H_{17}O_2N_8$	9.38877493188216	10.50	4.81	329.14690	2	40.83	3	0	42.57	85.67	(Collection)
329.1485	$C_{18}H_{21}O_4N_2$	31.7117293765914	9.50	-3.35	329.14958	1	49.08	3	0	50.04	98.08	(Collection)

Mass matching for adduct **27** + H$^+$ (19 hours).

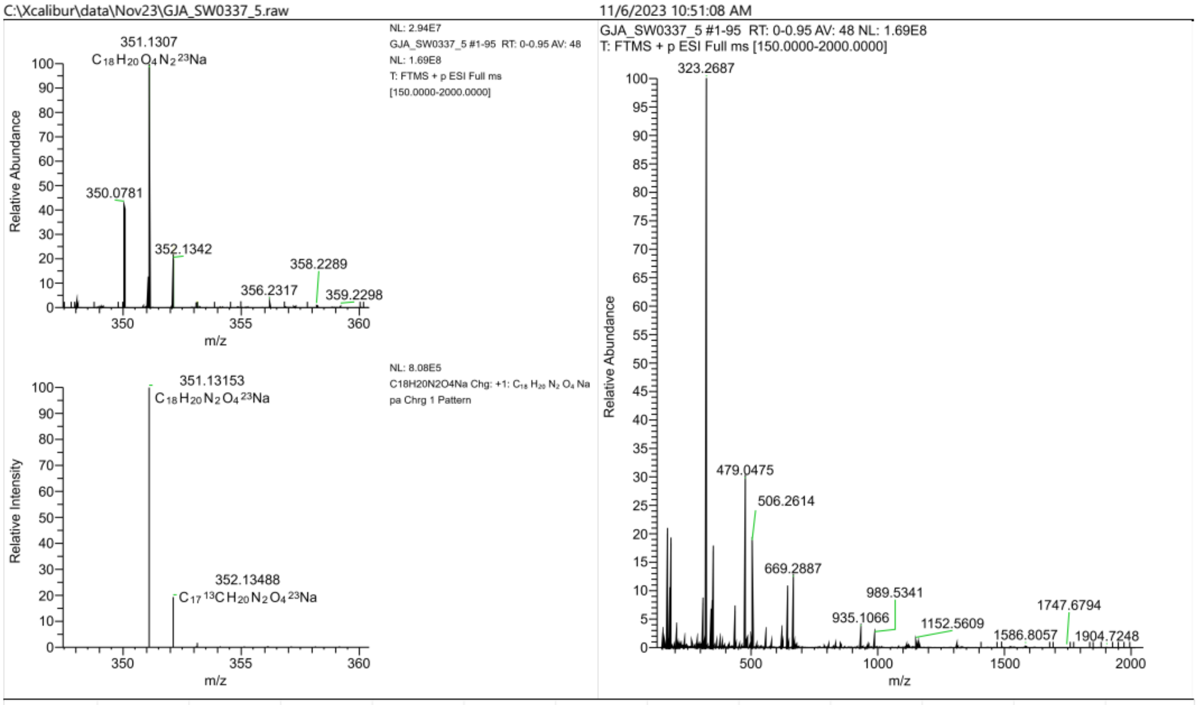

Peak Mass	Display For...	S Fit	RDB	Delta [ppm]	Theo. mass	Rank	Combined S...	# Matched I...	# Missed Iso.	MS Cov. [%]	Pattern Cov....	MSMS Matc...
351.1307	$C_{16}H_{15}O_2N_8$	70.21279318 89808	13.50	-1.43	351.13125	1	94.89	4	0	96.26	100	(Collection)
351.1307	$C_{18}H_{20}O_4N_2{}^{23}Na$	42.24410133 08871	9.50	-2.23	351.13153	2	93.31	4	0	96.15	99.8	(Collection)

Mass matching for adduct **27** + Na^+ (19 hours).

General Procedure for Raman Experiments

A solution of fluorene **10** (0.125 M), NHPI **1** (0.012 M), acetic acid (0.125 M) and 1,4-dioxane (0.129 M) in acetonitrile was added to a 250 mL jacketed vessel. The mixture was heated to 50 °C using a recirculating water bath. The Raman probe was positioned on the side of the vessel and the vessel was covered in blackout fabric. Aqueous sodium chlorite (0.375 M) was added to the mixture *via* a dropping funnel and Raman spectra acquired using a Kaiser RXN1 Raman spectrometer with a 785 nm laser, fibre-coupled to a PhAT probe with a collimated beam and 6 mm diameter laser spot every 30 or 60 seconds. The spectra were analysed between 700–1650 cm^{-1}. The raw data was processed by taking a first derivative, applying a Savitzky-Golay filter (window length = 21, polynomial order = 2, as implemented in SciPy) and scaling the signal intensity by the 1,4-dioxane signal. The concentration of fluorene **10**, fluorenone **11** and chlorine dioxide was determined based on the relative intensity of diagnostic signals relative to the 1,4-dioxane signal at 829.80 cm^{-1}. Each experiment was conducted in duplicate and the results averaged to give a time-course.

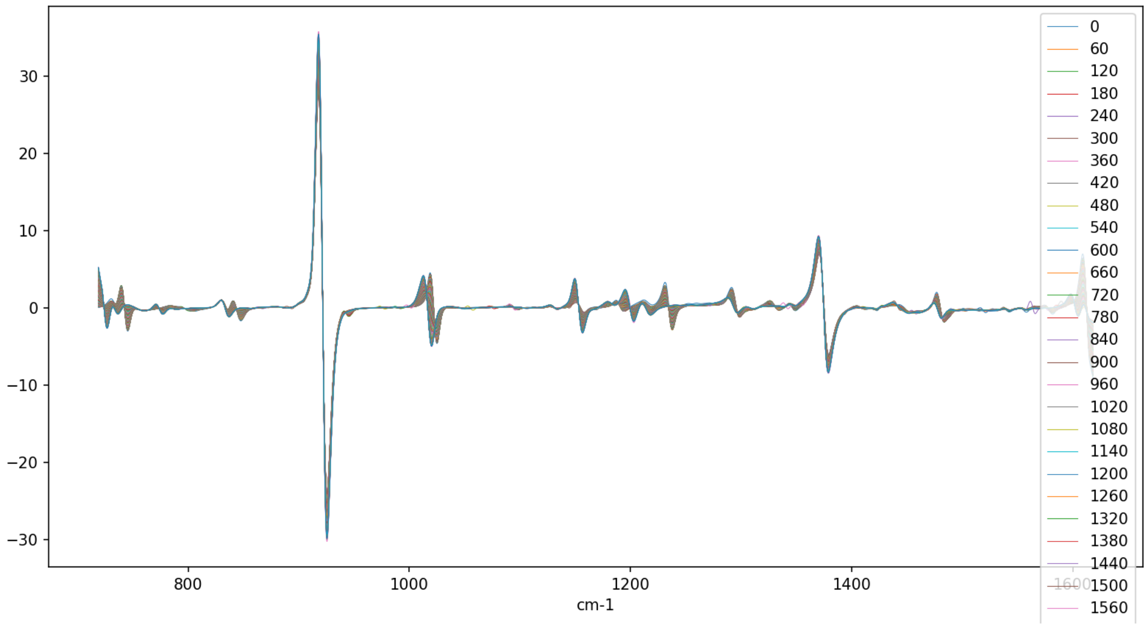

Typical Raman time course trace: **10** 1 equiv, NHPI **1** 10 mol%, $NaClO_2$ 1.5 equiv, AcOH 1 equiv, $MeCN:H_2O$ (2:1), 50 °C.

Peaks at 745.20, 1237.80 and 1608.90 cm^{-1} correspond to fluorene **10**.

Peaks at 718.80, 726.30 and 1012.50 cm^{-1} correspond to fluorenone **11**.

Peak at 945.60 cm^{-1} corresponds to chlorine dioxide.

Raman calibration curves were prepared showing relative intensity of the above signals (*vs.* 1,4-dioxane) for serial dilutions of standard solutions of fluorene **10**, fluorenone **11** and chlorine dioxide.

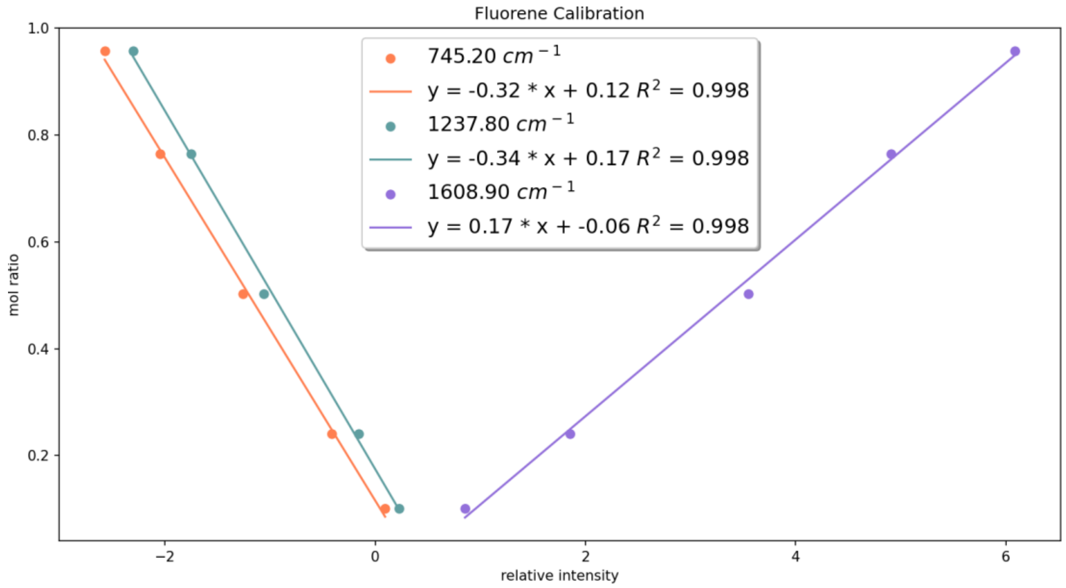
Fluorene Calibration
745.20 cm^{-1}
y = -0.32 * x + 0.12 R^2 = 0.998
1237.80 cm^{-1}
y = -0.34 * x + 0.17 R^2 = 0.998
1608.90 cm^{-1}
y = 0.17 * x + -0.06 R^2 = 0.998
mol ratio
relative intensity

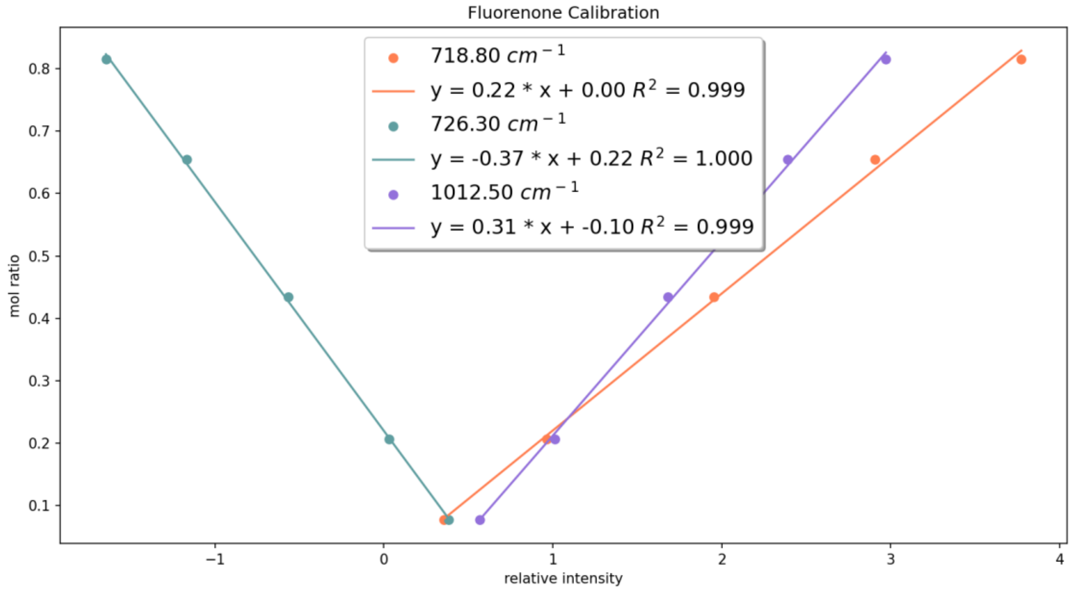
Fluorenone Calibration
718.80 cm^{-1}
y = 0.22 * x + 0.00 R^2 = 0.999
726.30 cm^{-1}
y = -0.37 * x + 0.22 R^2 = 1.000
1012.50 cm^{-1}
y = 0.31 * x + -0.10 R^2 = 0.999
mol ratio
relative intensity

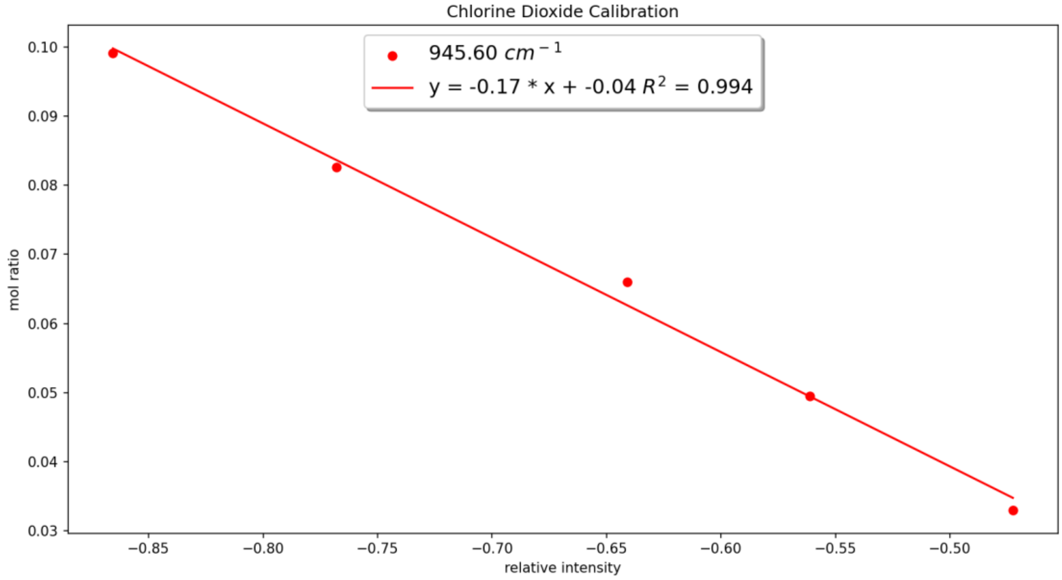
Chlorine Dioxide Calibration
945.60 cm^{-1}
y = -0.17 * x + -0.04 R^2 = 0.994
mol ratio
relative intensity

Raman Experiments

Temperature Study: 10 1 equiv, NHPI **1** 10 mol%, $NaClO_2$ 1.5 equiv, AcOH 1 equiv, MeCN:H_2O (2:1).

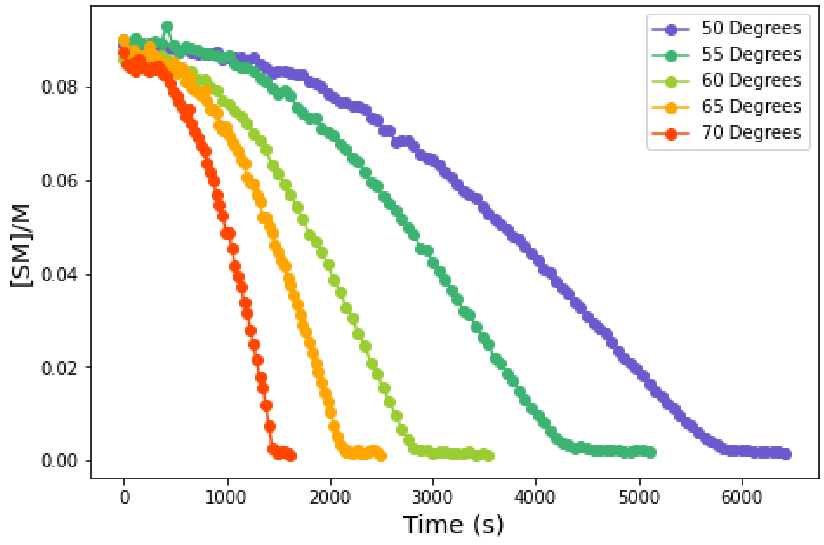

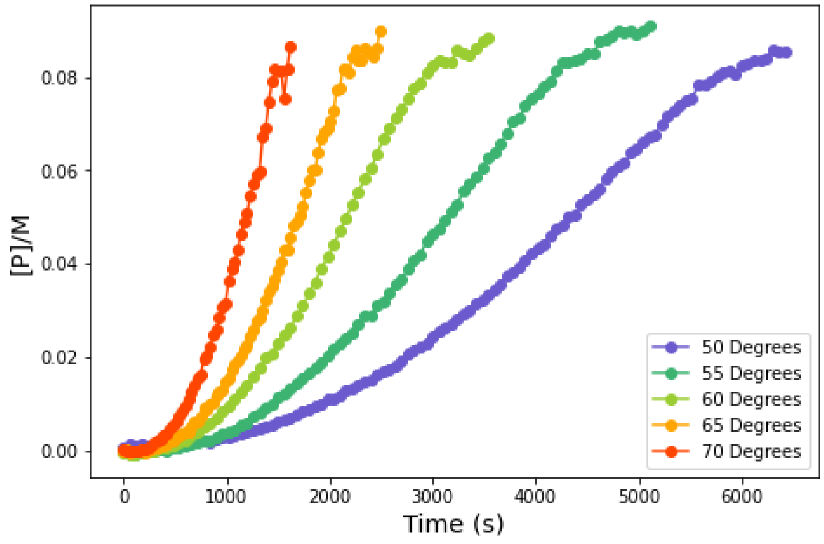

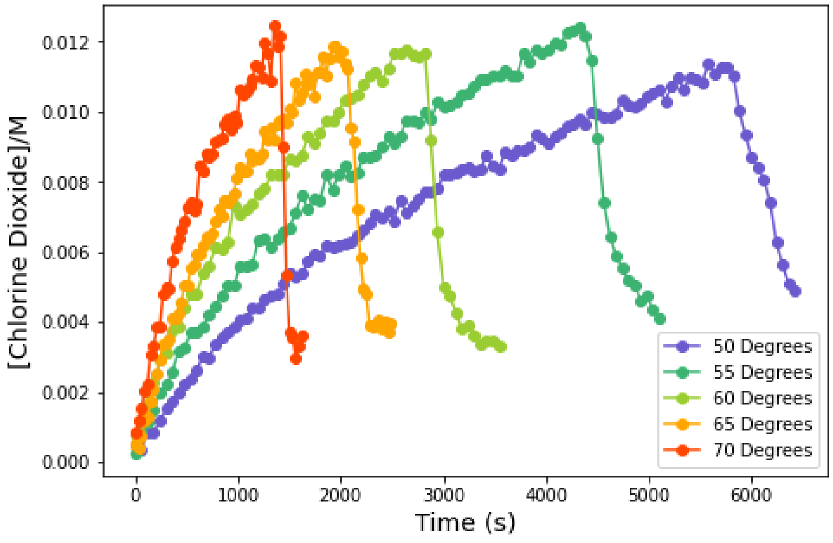

pH 4.5 Buffer: 10 1 equiv, NHPI **1** 10 mol%, $NaClO_2$ 1.5 equiv, MeCN:pH 4.5 buffer (2:1), 50 °C.

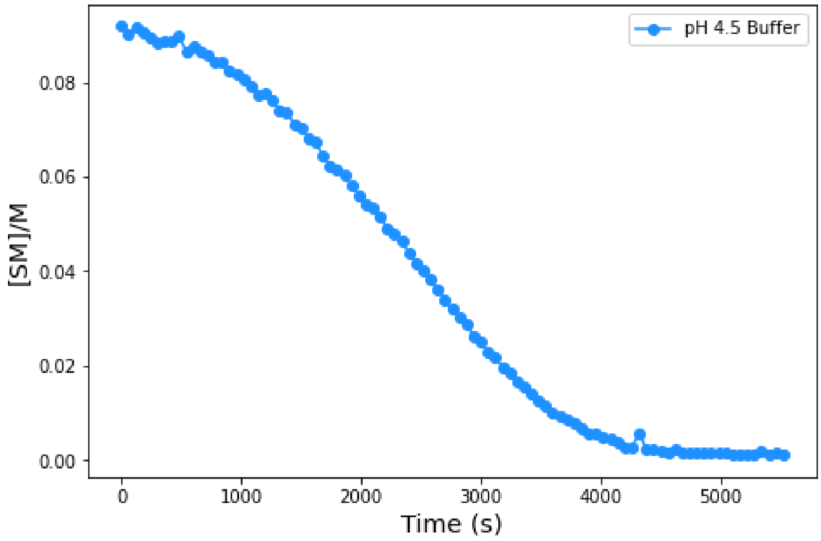

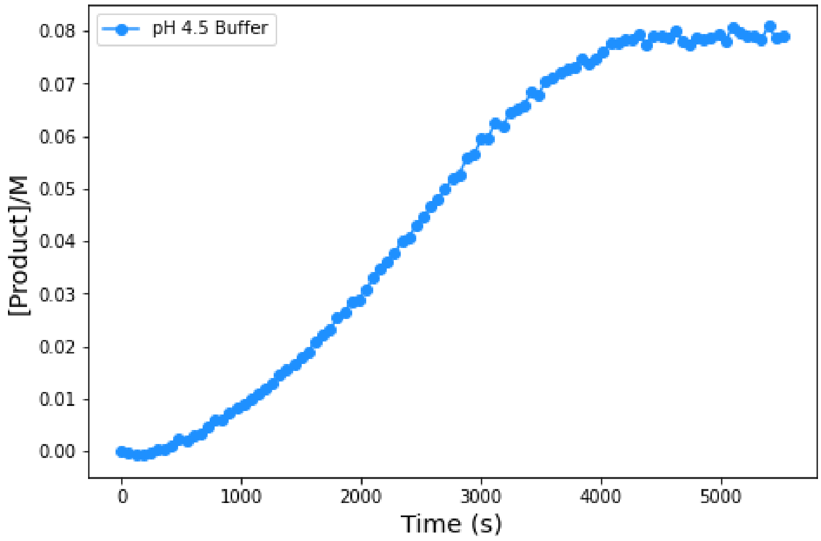

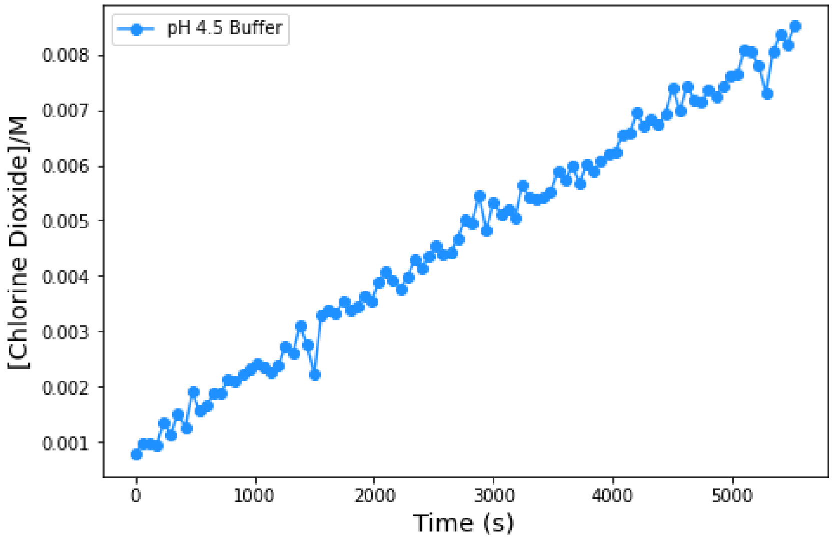

NaClO Additive: 10 1 equiv, NHPI **1** 10 mol%, $NaClO_2$ 1.5 equiv, NaClO 10 mol%, MeCN:pH 4.5 buffer (2:1), 50 °C.

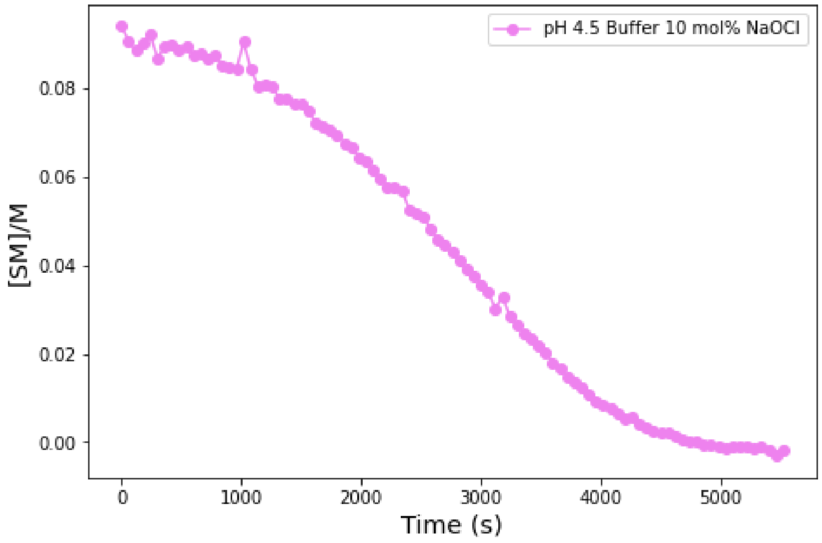

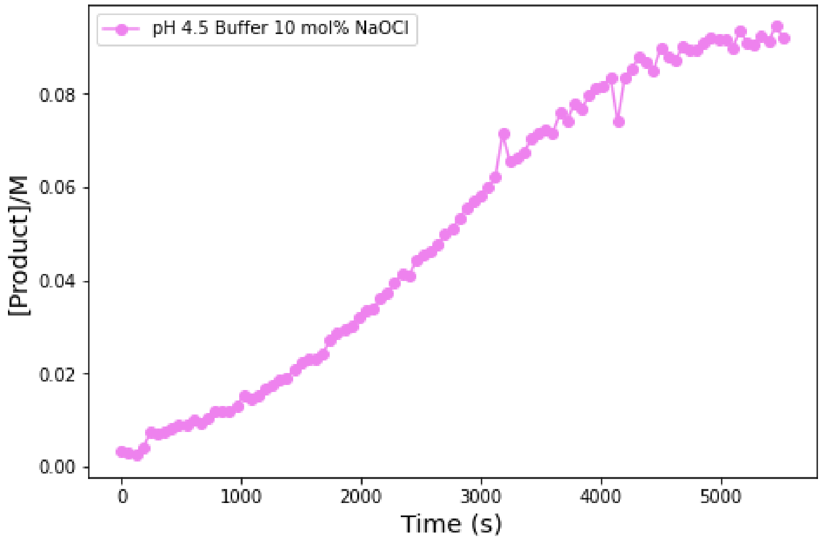

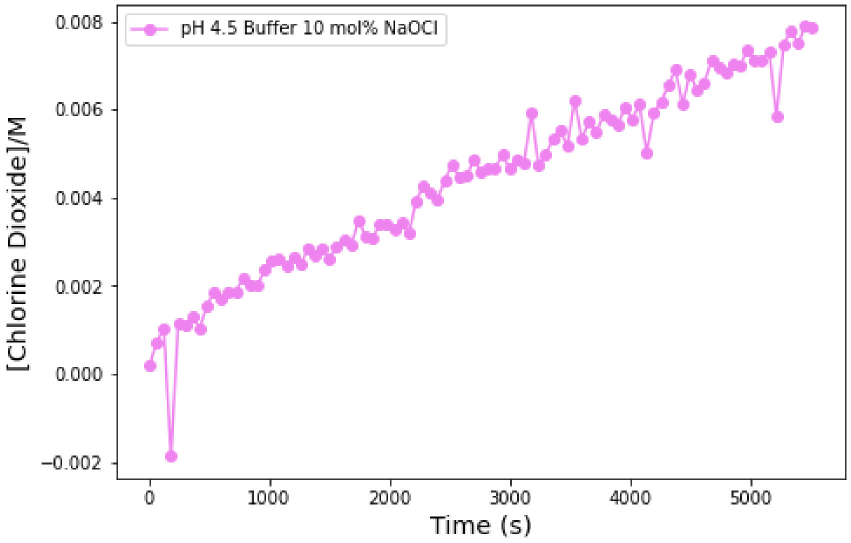

Reaction of sodium chlorite sourced from different suppliers

Sigma Aldrich: 10 1 equiv, NHPI **1** 10 mol%, $NaClO_2$ 1.5 equiv, AcOH 1 equiv, MeCN:H_2O (2:1), 50 °C.

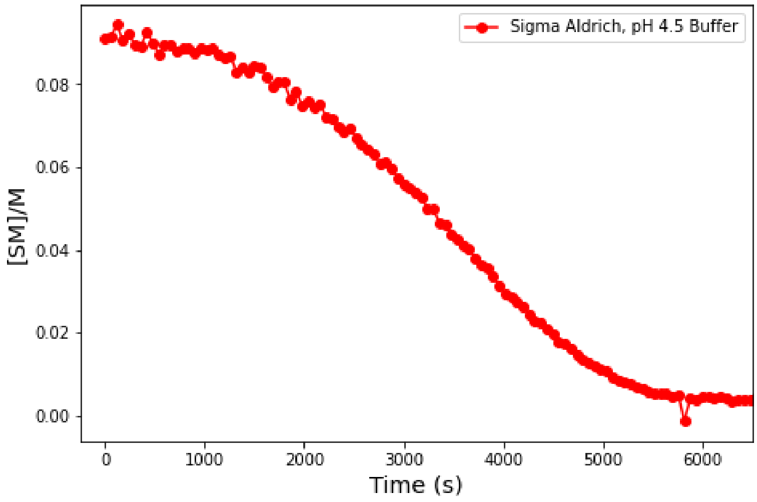

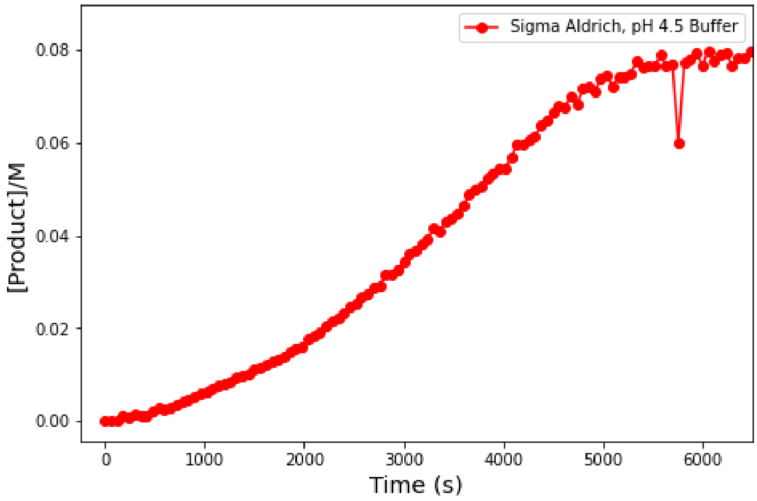

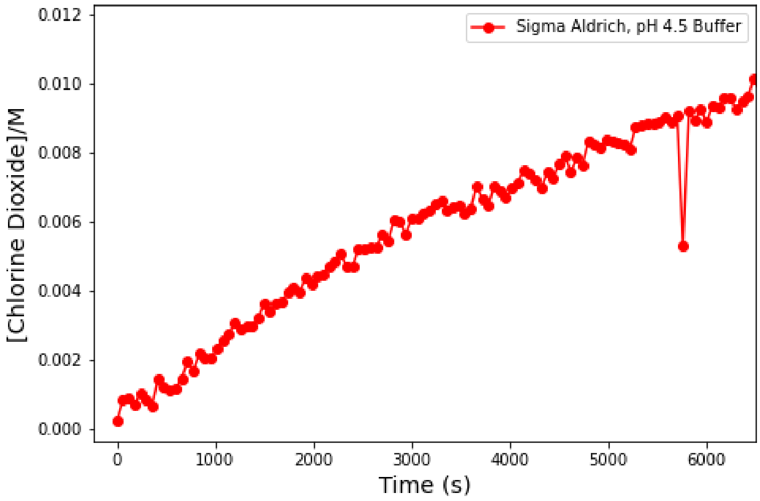

Thermo Fisher: 10 1 equiv, NHPI **1** 10 mol%, $NaClO_2$ 1.5 equiv, AcOH 1 equiv, $MeCN:H_2O$ (2:1), 50 °C.

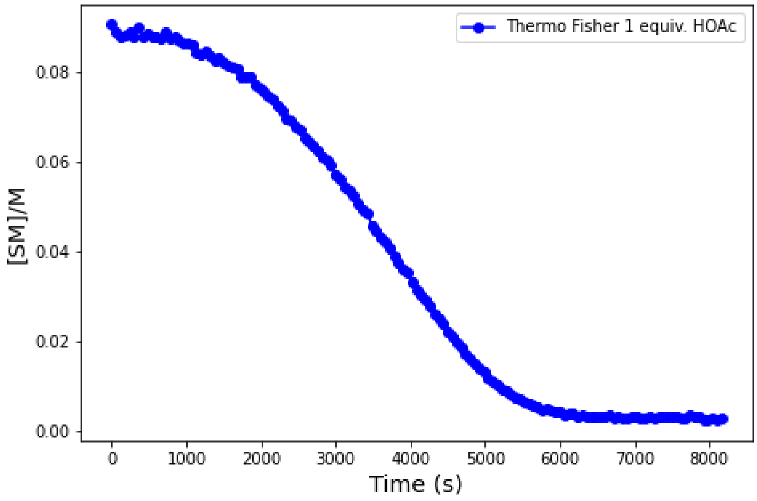

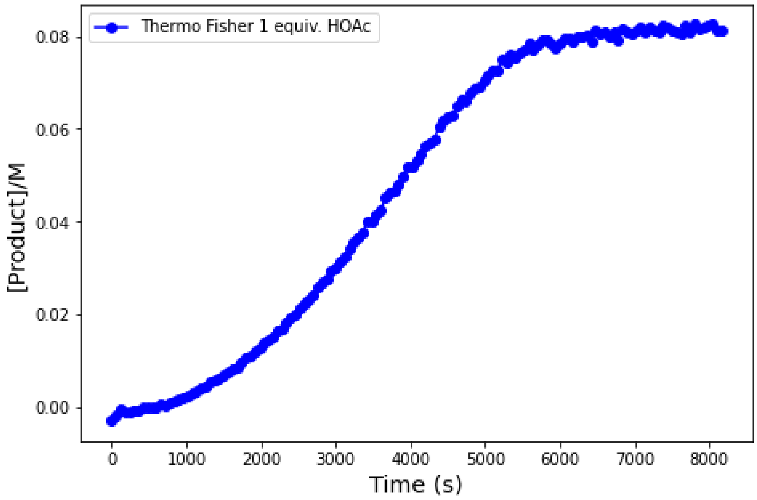

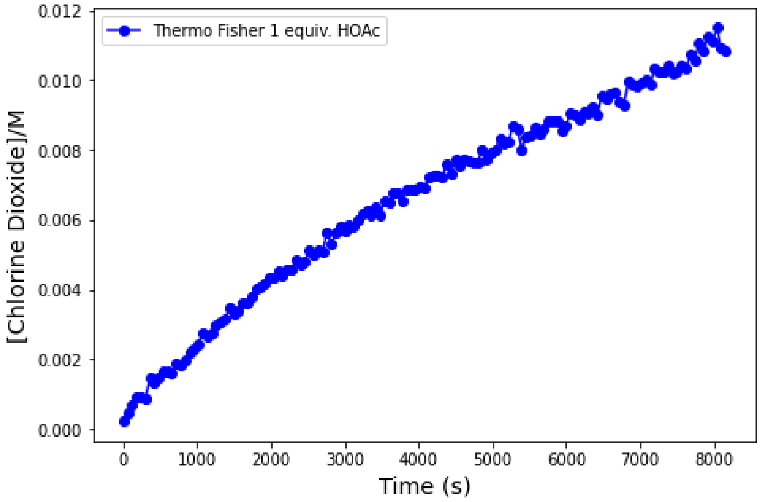

Fluka: 10 1 equiv, NHPI **1** 10 mol%, $NaClO_2$ 1.5 equiv, AcOH 1 equiv, MeCN:H_2O (2:1), 50 °C.

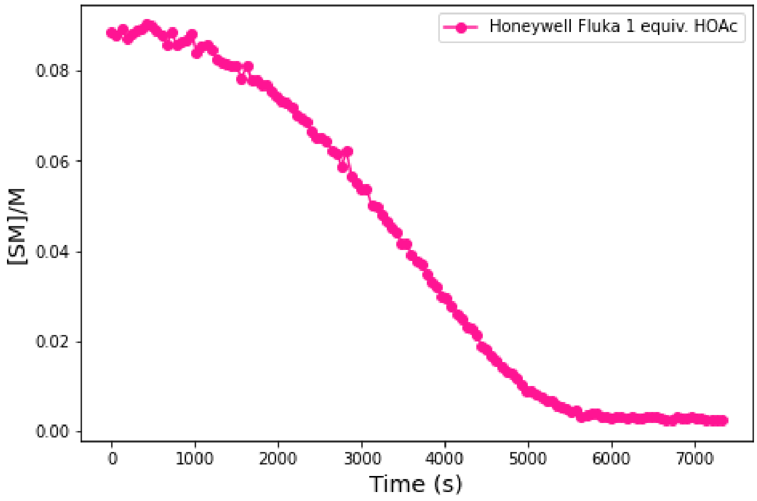

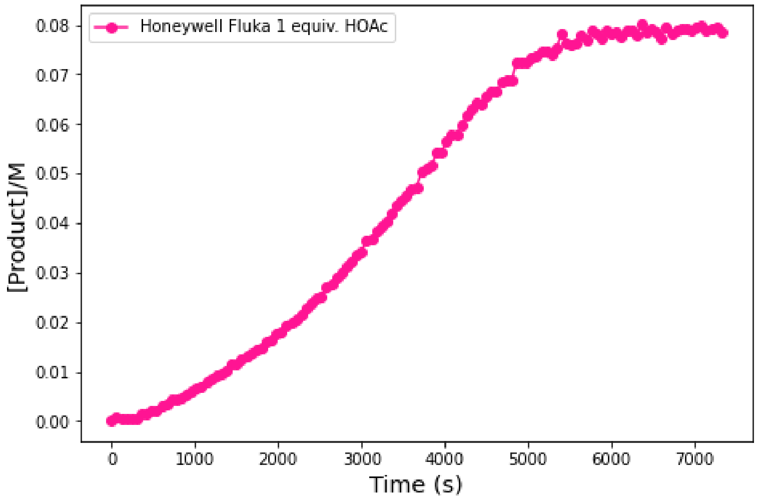

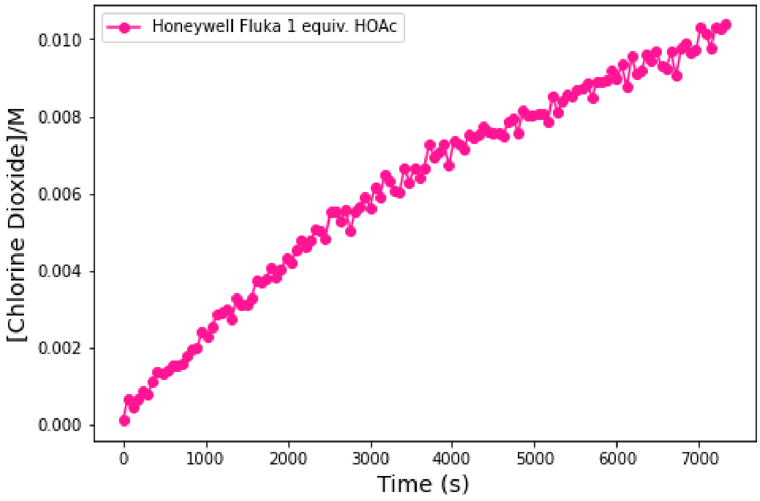

Sigma Aldrich: 10 1 equiv, NHPI **1** 10 mol%, $NaClO_2$ 1.5 equiv, MeCN:pH 4.5 buffer (2:1), 50 °C.

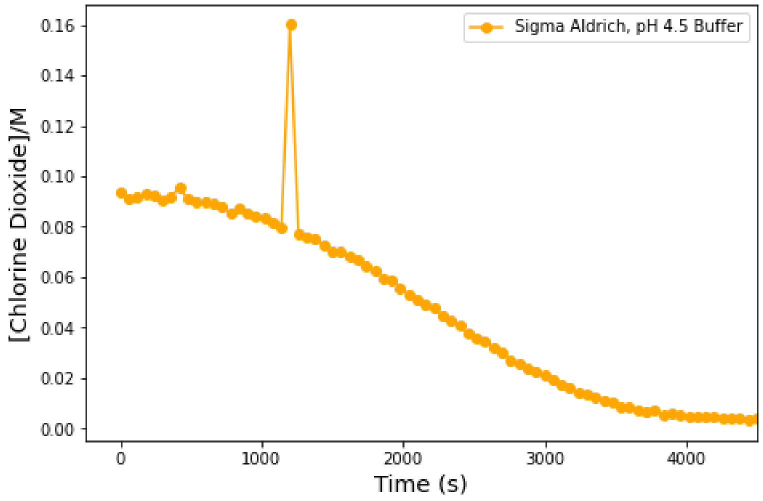

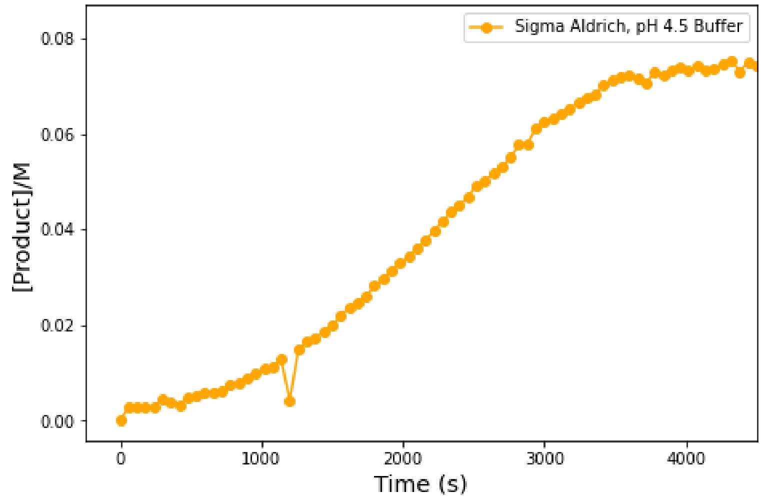

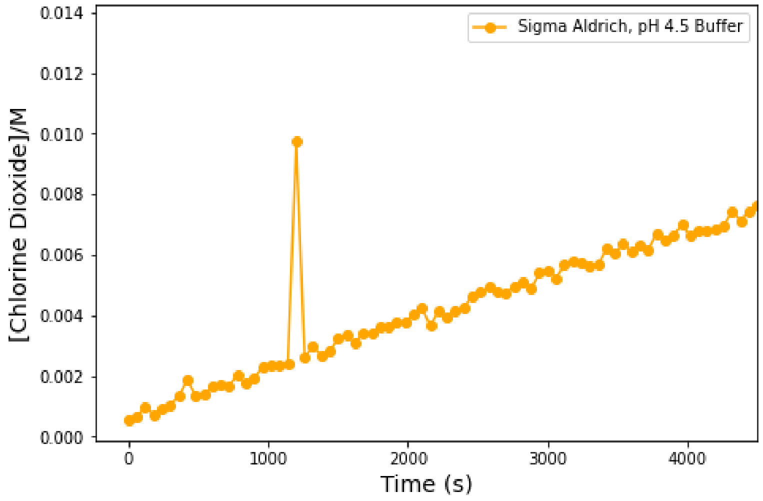

Thermo Fisher: 10 1 equiv, NHPI **1** 10 mol%, $NaClO_2$ 1.5 equiv, MeCN:pH 4.5 buffer (2:1), 50 °C.

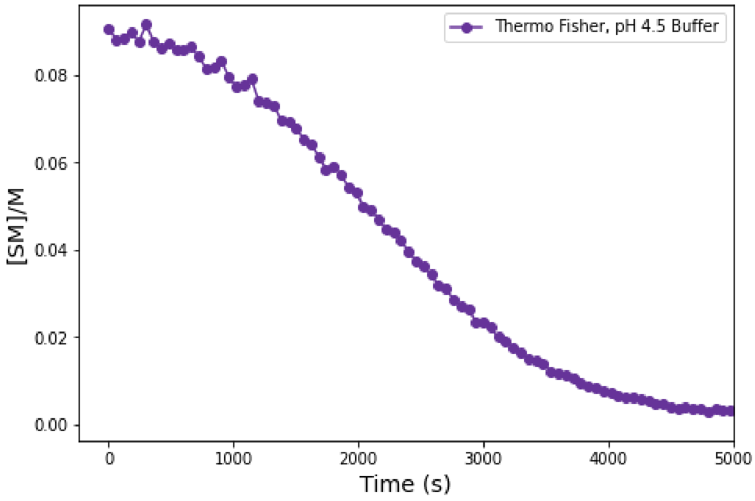

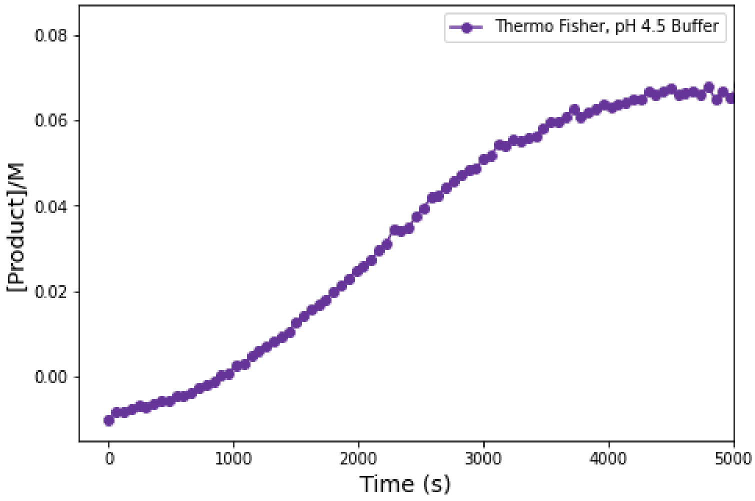

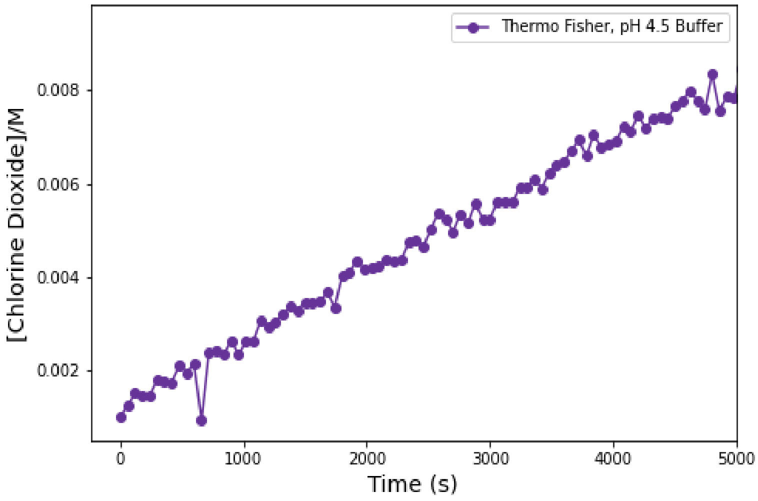

Reaction under the conditions reported by Salvador.

10 1 equiv, NHPI **1** 10 mol%, $NaClO_2$ 1.5 equiv, MeCN:H_2O (2:1), 50 °C.

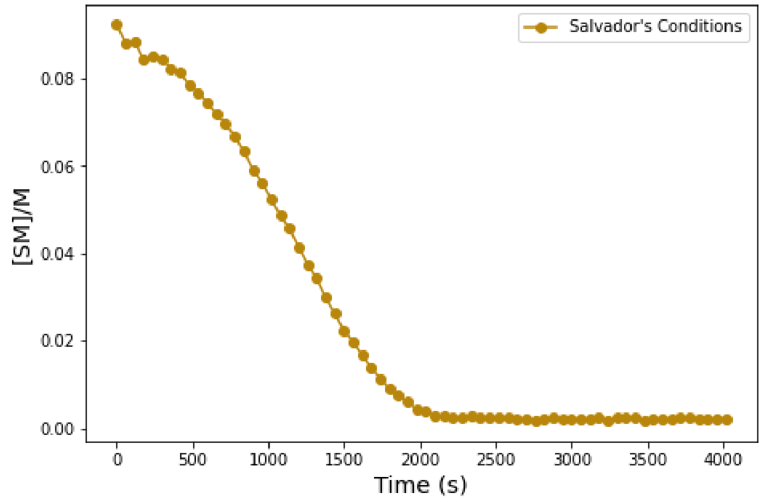

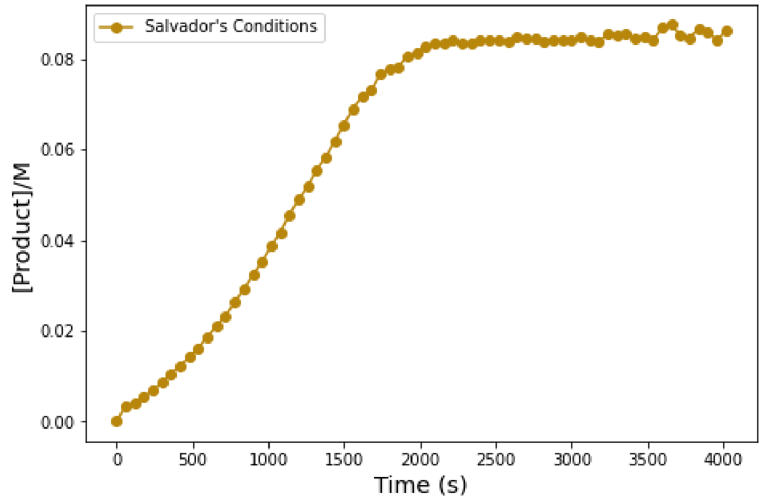

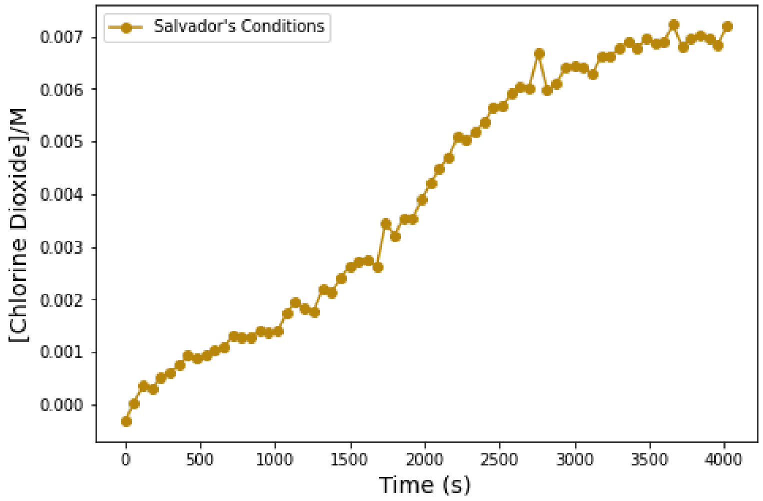

Reaction in the presence of 20 mol% NHPI catalyst **1**.

10 1 equiv, NHPI **1** 20 mol%, $NaClO_2$ 1.5 equiv, AcOH 1 equiv, MeCN:H_2O (2:1), 50 °C.

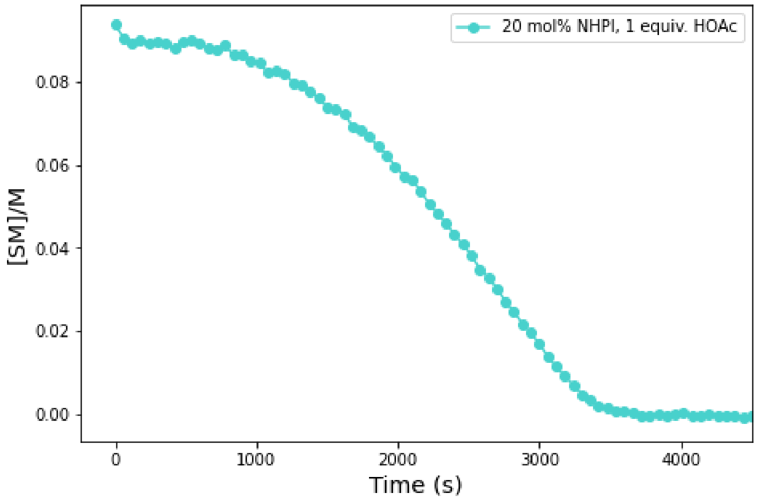

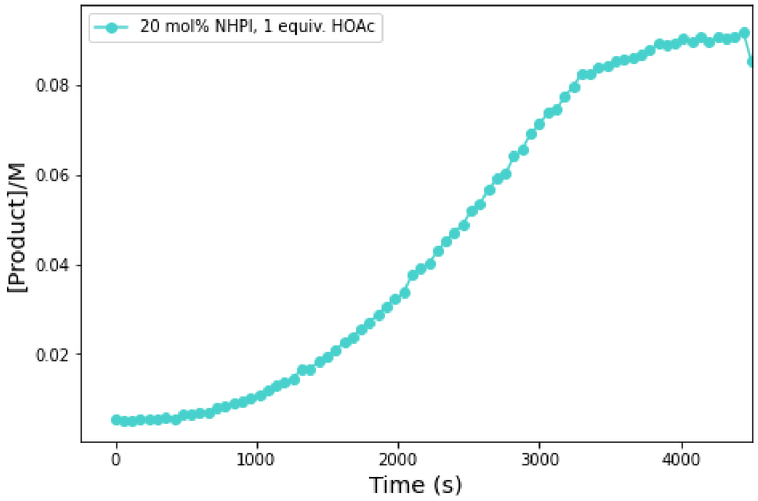

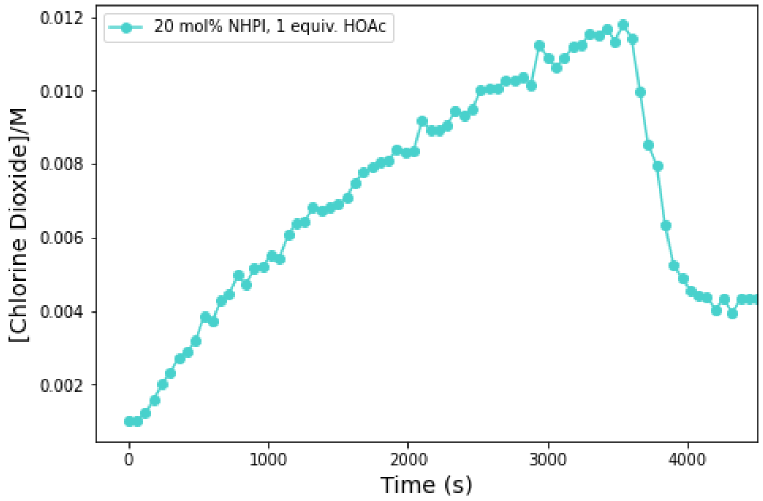

Computational Methods

Calculations were conducted using GAUSSIAN16 software package. Geometry optimisation followed by frequency calculations using UM06-2X-D3/6-311++G(d,p) with C-PCM acetonitrile implicit solvation model. Optimised structures were confirmed as energy minima by absence of imaginary frequencies in the vibrational analysis. Transition states were confirmed as first order saddle points on the potential energy surface by the presence of only one imaginary frequency in the vibrational analysis. Calculated transition states were confirmed as true by following the intrinsic reaction coordinate (IRC).

Compound	Calculation Type	File Ref
Fluorene **10**	Ground state	SW3_2
Chlorine dioxide	Ground state	SW7_3
NHPI **1**	Ground state	SW42_2
PINO anion **20**	Ground state	SW347
Chlorous acid	Ground state	SW47_2
Fluorene radical **14**	Ground state	SW4_2
Fluorene-chlorine dioxide adduct **19**	Ground state	SW416
Fluorenone **11**	Ground state	SW446
Hypochlorous acid	Ground state	SW445
Chlorite anion	Ground state	SW349
NHPI + chlorine dioxide -> PINO radical HAT **TS1**$_{\textbf{(OH HAT)}}$	Transition state	SW359_8
PINO radical + Fluorene -> NHPI + Fluorene radical **TS2**	Transition state	SW102_5
Chlorine dioxide + Fluorene -> Chlorous Acid + Fluorene radical **TS1**$_{\textbf{(CH HAT)}}$	Transition state	SW88_2
Fluorene radical + Chlorine Dioxide -> Fluorene-Chlorine Dioxide Adduct **TS3**	Transition state	SW441
Fluorene-chlorine dioxide adduct -> Fluorenone + Hypochlorous acid **TS4**	Transition state	SW417
Chlorine dioxide in chlorite anion geometry	Nielsen Four Point Analysis	SW350
Chlorite anion in chlorine dioxide geometry	Nielsen Four Point Analysis	SW348
PINO anion in PINO radical geometry	Nielsen Four Point Analysis	SW351
PINO radical in PINO anion geometry	Nielsen Four Point Analysis	SW346

The single electron transfer from the PINO anion **20** to chlorine dioxide was predicted using Nelsen's four point Marcus treatment of the single electron oxidation using equation 1.[7]

$$\Delta G^{\ddagger} = \frac{\lambda_i}{4}\left(1 + \frac{\Delta G}{\lambda_i}\right)^2 \quad (1)$$

Where $\Delta G^{\ddagger}$ is the barrier to single electron transfer, ΔG is the relative free energy of the products (the chlorite anion and PINO radical **2**) to the starting materials and λ_i is the internal reorganization energy of the whole system, given by equation 2.

$$\lambda_i = \frac{1}{2}(\lambda_i PINO + \lambda_i Chlorine\ Dioxide) \quad (2)$$

The internal reorganization energy, λ_i, of each species is calculated using equation 3. Where $E_s(R_R)$ is the energy of the starting species in the geometry of its product, $E_s(R_S)$ is the energy of the starting species in its optimized geometry, $E_R(R_s)$ is the energy of the product species in the optimized geometry of the starting species and $E_R(R_R)$ is the energy of the product species in its optimized geometry.

$$\lambda_i Species = \left(E_s(R_R) - E_s(R_S)\right) + \left(E_R(R_S) - E_R(R_R)\right) \quad (3)$$

Fluorene 10

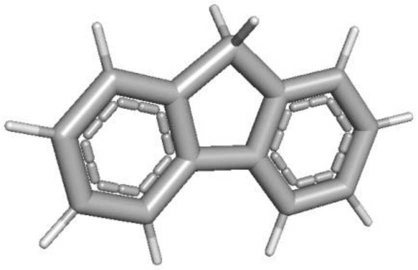

Center Number	Atomic Number	Atomic Type	Coordinates (Angstroms) X	Y	Z
1	6	0	1.208908	-0.000015	3.007004
2	6	0	1.502561	-0.000057	1.645442
3	6	0	0.448294	0.000125	0.735794
4	6	0	-0.883829	-0.000081	1.179616
5	6	0	-1.171092	0.000021	2.537187
6	6	0	-0.115286	0.000105	3.449624
7	1	0	2.015491	-0.000136	3.730748
8	1	0	2.532341	-0.000216	1.305547
9	1	0	-2.197764	0.000174	2.886811
10	1	0	-0.324429	0.000288	4.512952
11	6	0	-1.828170	-0.000191	0.000000
12	1	0	-2.478440	0.879812	0.000000
13	1	0	-2.478503	-0.880056	-0.000000
14	6	0	-0.883829	-0.000081	-1.179616
15	6	0	0.448294	0.000125	-0.735794
16	6	0	-1.171092	0.000021	-2.537187
17	6	0	1.502561	-0.000057	-1.645442
18	6	0	-0.115286	0.000105	-3.449624
19	1	0	-2.197764	0.000174	-2.886811
20	6	0	1.208908	-0.000015	-3.007004
21	1	0	2.532341	-0.000216	-1.305547
22	1	0	-0.324429	0.000288	-4.512952
23	1	0	2.015491	-0.000136	-3.730748

Energy: -501.333979781

Zero-point correction=	0.188791 (Hartree/Particle)
Thermal correction to Energy=	0.197733
Thermal correction to Enthalpy=	0.198677
Thermal correction to Gibbs Free Energy=	0.154637

Chlorine Dioxide

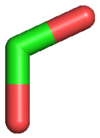

Center Number	Atomic Number	Atomic Type	Coordinates (Angstroms) X	Y	Z
1	8	0	0.000000	1.261553	-0.413684
2	17	0	0.000000	-0.000000	0.389350
3	8	0	-0.000000	-1.261553	-0.413684

Energy: -610.398036067

Zero-point correction= 0.005701 (Hartree/Particle)

Thermal correction to Energy= 0.008868

Thermal correction to Enthalpy= 0.009812

Thermal correction to Gibbs Free Energy= -0.019390

NHPI 1

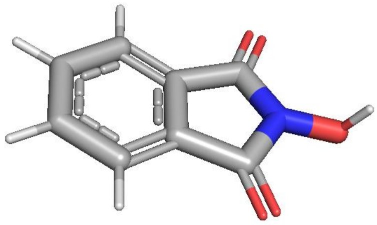

Center Number	Atomic Number	Atomic Type	X	Y	Z
1	6	0	-2.876719	0.697344	0.000179
2	6	0	-1.680802	1.421016	0.004440
3	6	0	-0.505385	0.696300	0.005820
4	6	0	-0.505382	-0.696300	0.005847
5	6	0	-1.680795	-1.421022	0.004492
6	6	0	-2.876716	-0.697355	0.000205
7	1	0	-3.820885	1.227769	-0.003687
8	1	0	-1.674145	2.503966	0.005037
9	1	0	-1.674134	-2.503971	0.005127
10	1	0	-3.820880	-1.227784	-0.003641
11	6	0	0.907686	-1.173204	0.008000
12	6	0	0.907678	1.173212	0.008006
13	7	0	1.668225	-0.000011	0.098579
14	8	0	1.352929	2.286376	-0.049806
15	8	0	1.352940	-2.286350	-0.049853
16	8	0	3.012827	-0.000016	-0.110288
17	1	0	3.425514	0.000073	0.764754

(Coordinates in Angstroms)

Energy: -588.202569814

Zero-point correction= 0.120638 (Hartree/Particle)

Thermal correction to Energy= 0.129801

Thermal correction to Enthalpy= 0.130745

Thermal correction to Gibbs Free Energy= 0.086193

PINO Anion 20

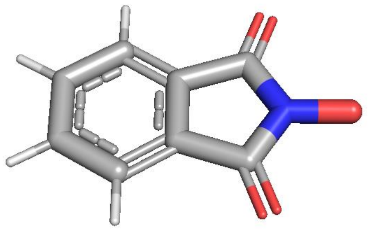

Center Number	Atomic Number	Atomic Type	Coordinates (Angstroms) X	Y	Z
1	6	0	0.000000	-2.843084	-0.695467
2	6	0	-0.000001	-1.640310	-1.418725
3	6	0	-0.000002	-0.467794	-0.695456
4	6	0	-0.000002	-0.467794	0.695456
5	6	0	-0.000001	-1.640310	1.418725
6	6	0	0.000000	-2.843084	0.695467
7	1	0	0.000002	-3.786495	-1.227981
8	1	0	-0.000001	-1.634475	-2.502184
9	1	0	-0.000001	-1.634475	2.502184
10	1	0	0.000002	-3.786495	1.227981
11	6	0	0.000000	0.960226	1.147422
12	6	0	0.000000	0.960226	-1.147422
13	7	0	0.000004	1.735755	0.000000
14	8	0	0.000000	1.379918	-2.287245
15	8	0	0.000000	1.379918	2.287245
16	8	0	-0.000001	3.063062	0.000000

Energy: -587.734190098

Zero-point correction= 0.107304 (Hartree/Particle)

Thermal correction to Energy= 0.116025

Thermal correction to Enthalpy= 0.116969

Thermal correction to Gibbs Free Energy= 0.073184

PINO Radical 2

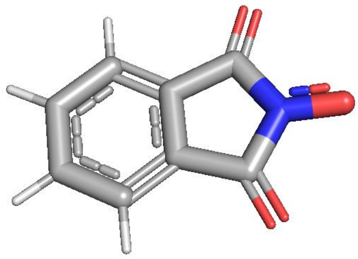

Center Number	Atomic Number	Atomic Type	Coordinates (Angstroms) X	Y	Z
1	6	0	-0.000033	-2.827089	-0.698595
2	6	0	0.000027	-1.634305	-1.421395
3	6	0	0.000040	-0.454653	-0.698390
4	6	0	0.000040	-0.454653	0.698390
5	6	0	0.000027	-1.634305	1.421395
6	6	0	-0.000033	-2.827089	0.698595
7	1	0	-0.000072	-3.771625	-1.228070
8	1	0	0.000020	-1.626940	-2.504076
9	1	0	0.000020	-1.626940	2.504076
10	1	0	-0.000072	-3.771625	1.228070
11	6	0	0.000029	0.936878	1.196973
12	6	0	0.000029	0.936878	-1.196973
13	7	0	-0.000097	1.751947	0.000000
14	8	0	0.000004	1.391219	-2.299528
15	8	0	0.000004	1.391219	2.299528
16	8	0	-0.000003	3.003003	0.000000

Energy: -587.563284086

Zero-point correction= 0.108183 (Hartree/Particle)

Thermal correction to Energy= 0.116923

Thermal correction to Enthalpy= 0.117867

Thermal correction to Gibbs Free Energy= 0.073380

Chlorous Acid

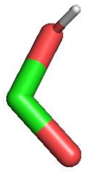

Center Number	Atomic Number	Atomic Type	Coordinates (Angstroms) X	Y	Z
1	8	0	-1.348272	0.343615	-0.120024
2	1	0	-1.584670	0.636259	0.775446
3	8	0	1.280142	0.556195	-0.009624
4	17	0	0.125277	-0.460867	0.015396

Energy: -611.040448044

Zero-point correction=	0.016609 (Hartree/Particle)
Thermal correction to Energy=	0.020344
Thermal correction to Enthalpy=	0.021289
Thermal correction to Gibbs Free Energy=	-0.009122

Fluorene Radical 14

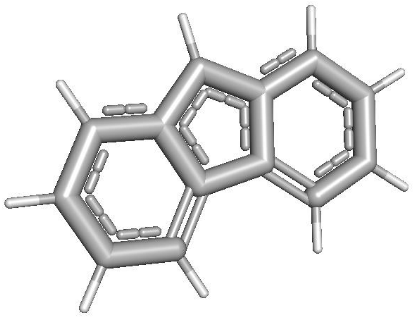

Center Number	Atomic Number	Atomic Type	Coordinates (Angstroms) X	Y	Z
1	6	0	-1.158722	-0.000005	3.023777
2	6	0	-1.483382	-0.000022	1.662761
3	6	0	-0.456077	-0.000008	0.734502
4	6	0	0.904134	0.000025	1.159110
5	6	0	1.215555	0.000047	2.523235
6	6	0	0.174502	0.000056	3.446651
7	1	0	-1.952149	-0.000030	3.761636
8	1	0	-2.521472	-0.000062	1.349204
9	1	0	2.248165	0.000060	2.853479
10	1	0	0.397345	0.000092	4.506935
11	6	0	-0.456077	-0.000008	-0.734502
12	6	0	0.904134	0.000025	-1.159110
13	6	0	-1.483382	-0.000022	-1.662761
14	6	0	1.215555	0.000047	-2.523235
15	6	0	-1.158722	-0.000005	-3.023777
16	1	0	-2.521472	-0.000062	-1.349204
17	6	0	0.174502	0.000056	-3.446651
18	1	0	2.248165	0.000060	-2.853479
19	1	0	-1.952149	-0.000030	-3.761636
20	1	0	0.397345	0.000092	-4.506935
21	6	0	1.746145	-0.000173	0.000000
22	1	0	2.827233	-0.000189	0.000000

Energy: -500.694181914

Zero-point correction= 0.175951 (Hartree/Particle)

Thermal correction to Energy= 0.184732

Thermal correction to Enthalpy= 0.185676

Thermal correction to Gibbs Free Energy= 0.141391

Fluorene-ClO_2 Adduct 19

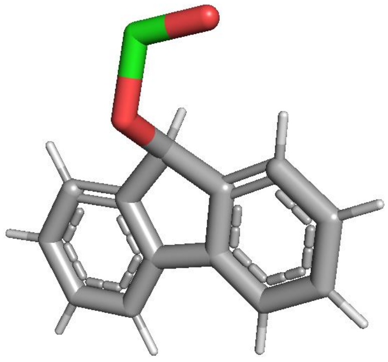

Center Number	Atomic Number	Atomic Type	Coordinates (Angstroms) X	Y	Z
1	6	0	-3.794534	-0.953710	-0.113850
2	6	0	-3.139340	0.278013	-0.131815
3	6	0	-1.758970	0.294849	0.022886
4	6	0	-1.053124	-0.900773	0.204195
5	6	0	-1.698717	-2.124462	0.221466
6	6	0	-3.085316	-2.142606	0.056519
7	1	0	-4.870616	-0.987776	-0.236142
8	1	0	-3.698438	1.196634	-0.267246
9	1	0	-1.143564	-3.045881	0.356905
10	1	0	-3.615252	-3.087283	0.063762
11	6	0	0.419209	-0.607311	0.381284
12	1	0	0.780852	-0.926079	1.363916
13	8	0	1.156993	-1.327830	-0.637831
14	8	0	3.665874	-0.817407	0.056108
15	17	0	2.630843	-1.949540	-0.106012
16	6	0	-0.796437	1.413558	0.023381
17	6	0	0.493100	0.896138	0.205722
18	6	0	1.603400	1.722634	0.216319
19	1	0	2.597957	1.307658	0.339467
20	6	0	1.407210	3.095823	0.049462
21	1	0	2.260254	3.763607	0.050839
22	6	0	0.124516	3.616119	-0.118966

23	1	0	-0.006504	4.684464	-0.243879
24	6	0	-0.992011	2.779778	-0.133571
25	1	0	-1.985869	3.190427	-0.269829

Energy: -1111.17188268

Zero-point correction= 0.186447 (Hartree/Particle)

Thermal correction to Energy= 0.199053

Thermal correction to Enthalpy= 0.199997

Thermal correction to Gibbs Free Energy= 0.145828

Fluorenone 11

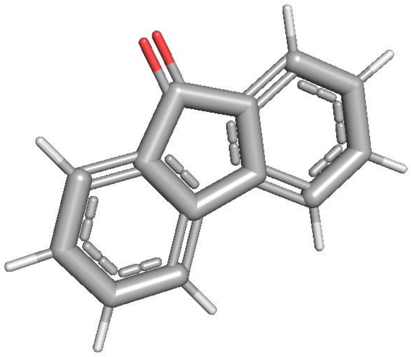

Center Number	Atomic Number	Atomic Type	Coordinates (Angstroms) X	Y	Z
1	6	0	-3.018462	-1.389990	-0.000019
2	6	0	-1.654750	-1.705893	0.000014
3	6	0	-0.742873	-0.664148	0.000036
4	6	0	-1.190387	0.664775	0.000034
5	6	0	-2.535669	0.982864	-0.000012
6	6	0	-3.457569	-0.067557	-0.000038
7	1	0	-3.747775	-2.191457	-0.000037
8	1	0	-1.331964	-2.740346	0.000017
9	1	0	-2.860546	2.017097	-0.000057
10	1	0	-4.519505	0.144929	-0.000085
11	6	0	0.742887	-0.664146	0.000010
12	6	0	1.190393	0.664782	-0.000028
13	6	0	1.654771	-1.705884	0.000026
14	6	0	2.535674	0.982881	-0.000039
15	6	0	3.018479	-1.389968	0.000002
16	1	0	1.331994	-2.740340	0.000067
17	6	0	3.457579	-0.067532	-0.000034
18	1	0	2.860542	2.017118	-0.000066
19	1	0	3.747798	-2.191431	0.000020
20	1	0	4.519514	0.144959	-0.000051
21	6	0	-0.000030	1.573449	0.000030
22	8	0	-0.000039	2.782209	0.000039

Energy: -575.351476115

Zero-point correction= 0.170431 (Hartree/Particle)

Thermal correction to Energy= 0.180029

Thermal correction to Enthalpy= 0.180973

Thermal correction to Gibbs Free Energy= 0.135221

Hypochlorous Acid

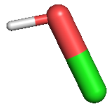

Center Number	Atomic Number	Atomic Type	Coordinates (Angstroms) X	Y	Z
1	8	0	0.036014	1.099824	-0.000000
2	1	0	-0.900344	1.343703	-0.000000
3	17	0	0.036014	-0.596606	0.000000

Energy: -535.947732955

Zero-point correction= 0.013350 (Hartree/Particle)

Thermal correction to Energy= 0.016282

Thermal correction to Enthalpy= 0.017227

Thermal correction to Gibbs Free Energy= -0.009586

Chlorite

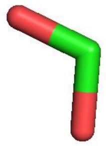

Center Number	Atomic Number	Atomic Type	Coordinates (Angstroms) X	Y	Z
1	8	0	0.000000	1.317385	-0.468792
2	17	0	-0.000000	-0.000000	0.441216
3	8	0	-0.000000	-1.317385	-0.468792

Energy: -610.589289146

Zero-point correction= 0.004450 (Hartree/Particle)

Thermal correction to Energy= 0.007780

Thermal correction to Enthalpy= 0.008724

Thermal correction to Gibbs Free Energy= -0.020281

Transition States

NHPI -> PINO ClO_2 HAT TS1$_{(OH\ HAT)}$

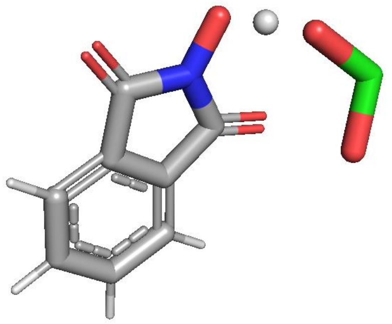

Center Number	Atomic Number	Atomic Type	Coordinates (Angstroms) X	Y	Z
1	6	0	3.952278	-0.426536	-0.654899
2	6	0	3.162224	0.724926	-0.639309
3	6	0	1.886592	0.608704	-0.120891
4	6	0	1.402708	-0.607223	0.365669
5	6	0	2.177150	-1.750984	0.353664
6	6	0	3.468565	-1.641868	-0.167346
7	1	0	4.958115	-0.377633	-1.052802
8	1	0	3.530704	1.670591	-1.016648
9	1	0	1.795986	-2.690894	0.732673
10	1	0	4.107472	-2.515678	-0.194440
11	6	0	0.012650	-0.417985	0.848515
12	6	0	0.833999	1.644717	0.019137
13	7	0	-0.265234	0.949348	0.608075
14	8	0	0.816588	2.805468	-0.264233
15	8	0	-0.778053	-1.181093	1.327563
16	8	0	-1.382232	1.523754	0.945217
17	1	0	-2.224032	1.216135	0.181715
18	8	0	-3.006706	0.830523	-0.635825
19	17	0	-3.409199	-0.677918	-0.377800
20	8	0	-2.366125	-1.631886	-0.936679

Energy: -1198.58345835

Zero-point correction= 0.121560 (Hartree/Particle)

Thermal correction to Energy= 0.134666

Thermal correction to Enthalpy= 0.135610

Thermal correction to Gibbs Free Energy= 0.078740

Imaginary Frequency: -8776.20

PINO + Fluorene -> Fluorene Radical + NHPI TS2

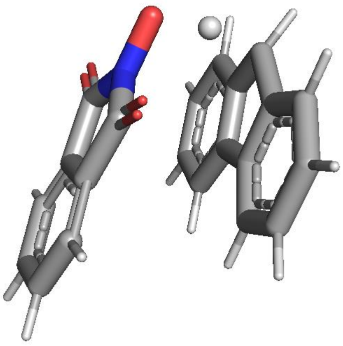

Center Number	Atomic Number	Atomic Type	Coordinates (Angstroms) X	Y	Z
1	6	0	0.415186	-3.031850	-1.603446
2	6	0	0.250719	-1.659866	-1.801922
3	6	0	1.075372	-0.787979	-1.104221
4	6	0	2.042880	-1.278957	-0.198513
5	6	0	2.207978	-2.648076	-0.014522
6	6	0	1.390411	-3.519596	-0.729512
7	1	0	-0.218635	-3.729217	-2.138438
8	1	0	-0.504678	-1.288761	-2.486128
9	1	0	2.947431	-3.028093	0.681251
10	1	0	1.502909	-4.589351	-0.600028
11	6	0	2.678008	-0.140769	0.491194
12	1	0	3.700281	-0.196533	0.857183
13	1	0	1.987424	-0.105036	1.589226
14	6	0	2.169018	1.061714	-0.194109
15	6	0	1.155147	0.680963	-1.102119
16	6	0	2.479986	2.404397	-0.004609
17	6	0	0.431187	1.638921	-1.798717
18	6	0	1.763014	3.361352	-0.718457
19	1	0	3.254470	2.700383	0.693853
20	6	0	0.743129	2.984411	-1.596214
21	1	0	-0.358037	1.353520	-2.485796
22	1	0	1.990240	4.412302	-0.585468

23	1	0	0.190247	3.747721	-2.130967
24	6	0	-3.904955	-0.502344	-0.765819
25	6	0	-2.961453	-1.273855	-0.085855
26	6	0	-1.952759	-0.599620	0.577968
27	6	0	-1.880725	0.791348	0.576372
28	6	0	-2.814125	1.564481	-0.089680
29	6	0	-3.832461	0.892892	-0.767810
30	1	0	-4.708076	-0.991842	-1.302719
31	1	0	-3.008642	-2.356008	-0.081449
32	1	0	-2.749263	2.645699	-0.088218
33	1	0	-4.580555	1.461382	-1.306371
34	6	0	-0.694325	1.217712	1.363914
35	6	0	-0.816111	-1.144145	1.365917
36	7	0	-0.071836	0.001875	1.753936
37	8	0	-0.546478	-2.271420	1.668852
38	8	0	-0.311099	2.312069	1.665289
39	8	0	1.030953	-0.055161	2.454293

Energy: -1088.88605488

Zero-point correction= 0.292979 (Hartree/Particle)

Thermal correction to Energy= 0.311455

Thermal correction to Enthalpy= 0.312399

Thermal correction to Gibbs Free Energy= 0.245826

Imaginary Frequency: -1976.49

ClO_2 + Fluorene -> Fluorene Radical + Chlorous Acid TS1$_{(CH\ HAT)}$

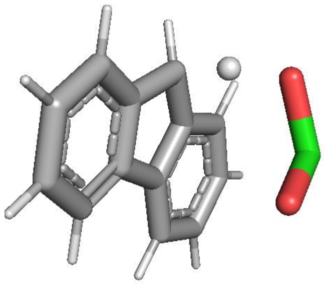

Center Number	Atomic Number	Atomic Type	Coordinates (Angstroms) X	Y	Z
1	6	0	-2.563652	-2.023355	-0.653578
2	6	0	-1.201520	-1.939370	-0.954776
3	6	0	-0.382732	-1.201437	-0.114316
4	6	0	-0.918981	-0.552257	1.023205
5	6	0	-2.281026	-0.635714	1.313013
6	6	0	-3.097176	-1.383326	0.467926
7	1	0	-3.217624	-2.596244	-1.300152
8	1	0	-0.800866	-2.443693	-1.826582
9	1	0	-2.694102	-0.127895	2.177252
10	1	0	-4.156406	-1.466706	0.677981
11	6	0	0.139001	0.226443	1.682236
12	1	0	0.144282	0.349647	2.763868
13	1	0	-0.127803	1.390053	1.295280
14	6	0	1.384621	-0.115116	0.951281
15	6	0	1.063259	-0.931600	-0.152439
16	6	0	2.692722	0.291161	1.174831
17	6	0	2.047218	-1.343905	-1.036992
18	6	0	3.684453	-0.131769	0.285879
19	1	0	2.941118	0.924973	2.018489
20	6	0	3.364883	-0.934015	-0.807811
21	1	0	1.808842	-1.974648	-1.885922
22	1	0	4.712222	0.170711	0.446164
23	1	0	4.147732	-1.248060	-1.487722

24	8	0	-0.532553	2.504964	0.647717
25	17	0	-1.211567	2.080388	-0.721767
26	8	0	-0.185844	1.957639	-1.857637

Energy: -1111.71191344

Zero-point correction= 0.189436 (Hartree/Particle)

Thermal correction to Energy= 0.202655

Thermal correction to Enthalpy= 0.203600

Thermal correction to Gibbs Free Energy= 0.147041

Imaginary Frequency: - 1763.87

Fluorene Radical + ClO_2 -> Fluorene-ClO_2 Adduct TS3

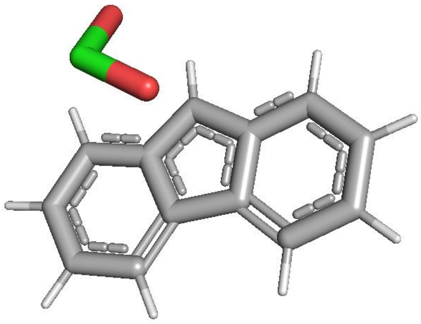

Center Number	Atomic Number	Atomic Type	Coordinates (Angstroms) X	Y	Z
1	6	0	-1.841635	2.794934	-0.296354
2	6	0	-0.473269	2.532812	-0.543705
3	6	0	0.070139	1.401602	0.002842
4	6	0	-0.729070	0.536331	0.798941
5	6	0	-2.078541	0.787252	1.024889
6	6	0	-2.630289	1.945957	0.467720
7	1	0	-2.286537	3.685491	-0.724506
8	1	0	0.109326	3.213529	-1.152095
9	1	0	-2.675150	0.091086	1.602370
10	1	0	-3.675850	2.177172	0.623415
11	6	0	1.430000	0.805072	-0.059707
12	6	0	1.393196	-0.397603	0.694540
13	6	0	2.591883	1.206799	-0.663251
14	6	0	2.518128	-1.197916	0.847623
15	6	0	3.732739	0.384893	-0.515134
16	1	0	2.652337	2.123209	-1.237339
17	6	0	3.702092	-0.790418	0.222714
18	1	0	2.475296	-2.112927	1.426162
19	1	0	4.658583	0.686901	-0.990232
20	1	0	4.597338	-1.391154	0.315368
21	6	0	0.075984	-0.556228	1.231246
22	1	0	-0.241070	-1.329282	1.916000
23	8	0	-0.513544	-1.620247	-1.437731

24	17	0	-1.971562	-1.863405	-0.859177
25	8	0	-1.819689	-2.403136	0.631817

Energy: -1111.10245434

Zero-point correction= 0.182688 (Hartree/Particle)

Thermal correction to Energy= 0.195738

Thermal correction to Enthalpy= 0.196682

Thermal correction to Gibbs Free Energy= 0.141740

Imaginary Frequency: - 124.32

Fluorene-ClO_2 Adduct -> Fluorenone + Hypochlorous Acid TS4

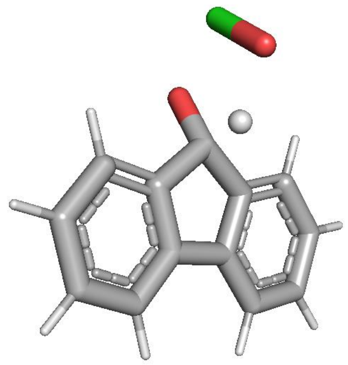

Center Number	Atomic Number	Atomic Type	Coordinates (Angstroms) X	Y	Z
1	6	0	0.745079	-0.002451	-0.128838
2	1	0	1.303768	-0.004120	0.895921
3	17	0	3.418563	-0.008505	-0.110846
4	8	0	2.876296	-0.007376	1.339633
5	8	0	1.704247	-0.005346	-1.072102
6	6	0	-0.189363	-1.194764	-0.087337
7	6	0	-1.503917	-0.733792	0.060774
8	6	0	0.101066	-2.545330	-0.160435
9	6	0	-2.555067	-1.640680	0.152635
10	6	0	-0.956600	-3.452696	-0.078278
11	1	0	1.122116	-2.890280	-0.281208
12	6	0	-2.268309	-3.002970	0.081835
13	1	0	-3.577640	-1.299963	0.269107
14	1	0	-0.758065	-4.516088	-0.137333
15	1	0	-3.074974	-3.723394	0.147302
16	6	0	-1.499682	0.741837	0.060704
17	6	0	-2.545559	1.654819	0.152426
18	6	0	-2.250870	3.015428	0.081649
19	6	0	-0.936564	3.457534	-0.078406
20	6	0	-0.182483	1.195179	-0.087395
21	6	0	0.115818	2.544026	-0.160492
22	1	0	1.138876	2.883051	-0.281155

23	1	0	-0.731851	4.519756	-0.137454
24	1	0	-3.053351	3.740516	0.147088
25	1	0	-3.570096	1.320046	0.268811

Energy: -1111.16056813

Zero-point correction= 0.183577 (Hartree/Particle)

Thermal correction to Energy= 0.195647

Thermal correction to Enthalpy= 0.196591

Thermal correction to Gibbs Free Energy= 0.144084

Imaginary Frequency: - 443.28

Nielsen Four Point Analysis

Chlorine Dioxide in Chlorite Geometry

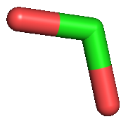

Center Number	Atomic Number	Atomic Type	Coordinates (Angstroms) X	Y	Z
1	8	0	0.000000	1.317385	-0.468792
2	17	0	-0.000000	0.000000	0.441216
3	8	0	-0.000000	-1.317385	-0.468792

Energy: -610.383909993

Chlorite in Chlorine Dioxide Geometry

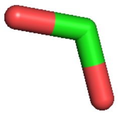

Center Number	Atomic Number	Atomic Type	Coordinates (Angstroms) X	Y	Z
1	8	0	0.000000	1.261553	-0.413684
2	17	0	0.000000	0.000000	0.389350
3	8	0	-0.000000	-1.261553	-0.413684

Energy: -610.574875163

PINO Anion in PINO Radical Geometry

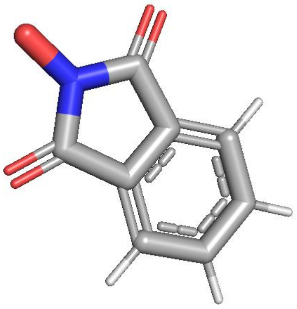

Center Number	Atomic Number	Atomic Type	Coordinates (Angstroms) X	Y	Z
1	6	0	0.000000	-2.843084	-0.695467
2	6	0	-0.000001	-1.640310	-1.418725
3	6	0	-0.000002	-0.467794	-0.695456
4	6	0	-0.000002	-0.467794	0.695456
5	6	0	-0.000001	-1.640310	1.418725
6	6	0	0.000000	-2.843084	0.695467
7	1	0	0.000002	-3.786495	-1.227981
8	1	0	-0.000001	-1.634475	-2.502184
9	1	0	-0.000001	-1.634475	2.502184
10	1	0	0.000002	-3.786495	1.227981
11	6	0	0.000000	0.960226	1.147422
12	6	0	0.000000	0.960226	-1.147422
13	7	0	0.000004	1.735755	0.000000
14	8	0	0.000000	1.379918	-2.287245
15	8	0	0.000000	1.379918	2.287245
16	8	0	-0.000001	3.063062	0.000000

Energy: -587.551502427

PINO Radical in PINO Anion Geometry

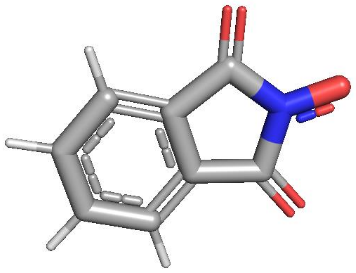

Center Number	Atomic Number	Atomic Type	Coordinates (Angstroms) X	Y	Z
1	6	0	-0.000033	-2.827089	-0.698595
2	6	0	0.000027	-1.634305	-1.421395
3	6	0	0.000040	-0.454653	-0.698390
4	6	0	0.000040	-0.454653	0.698390
5	6	0	0.000027	-1.634305	1.421395
6	6	0	-0.000033	-2.827089	0.698595
7	1	0	-0.000072	-3.771625	-1.228070
8	1	0	0.000020	-1.626940	-2.504076
9	1	0	0.000020	-1.626940	2.504076
10	1	0	-0.000072	-3.771625	1.228070
11	6	0	0.000029	0.936878	1.196973
12	6	0	0.000029	0.936878	-1.196973
13	7	0	-0.000097	1.751947	-0.000000
14	8	0	0.000004	1.391219	-2.299528
15	8	0	0.000004	1.391219	2.299528
16	8	0	-0.000003	3.003003	-0.000000

Energy: -587.721898951

Copies of NMR Spectra

Fluorenone 11

^{1}H NMR, $CDCl_3$, 400 MHz.

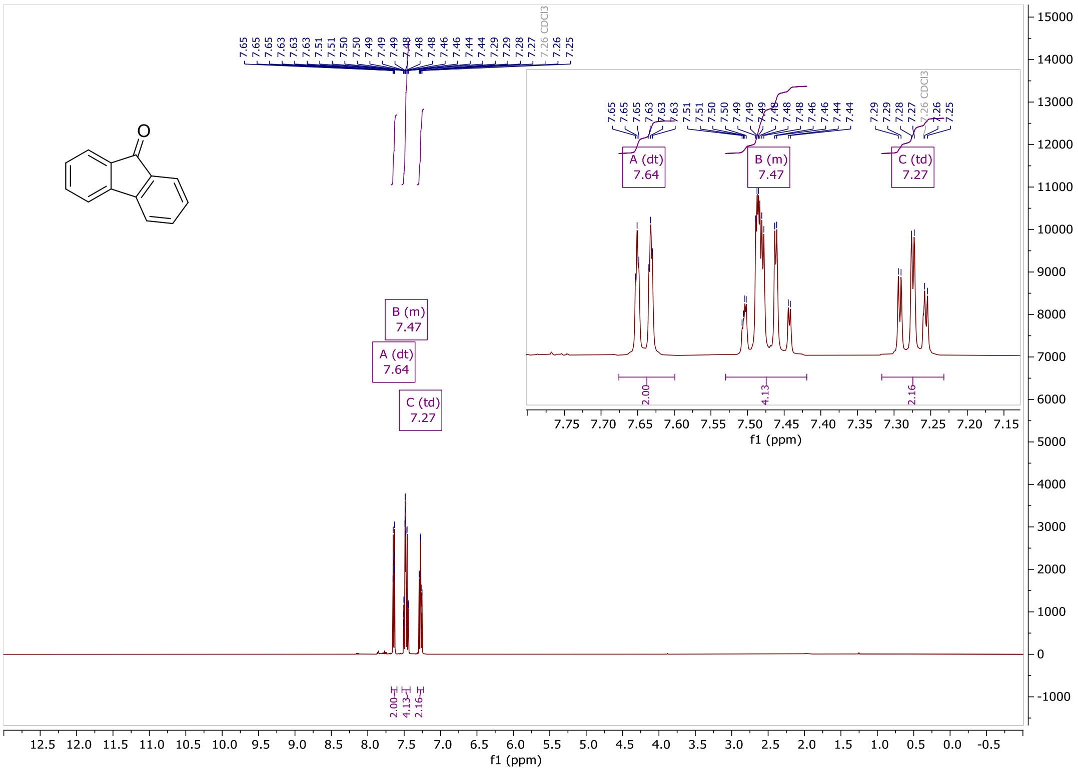

^{13}C{^{1}H} NMR, $CDCl_3$, 101 MHz.

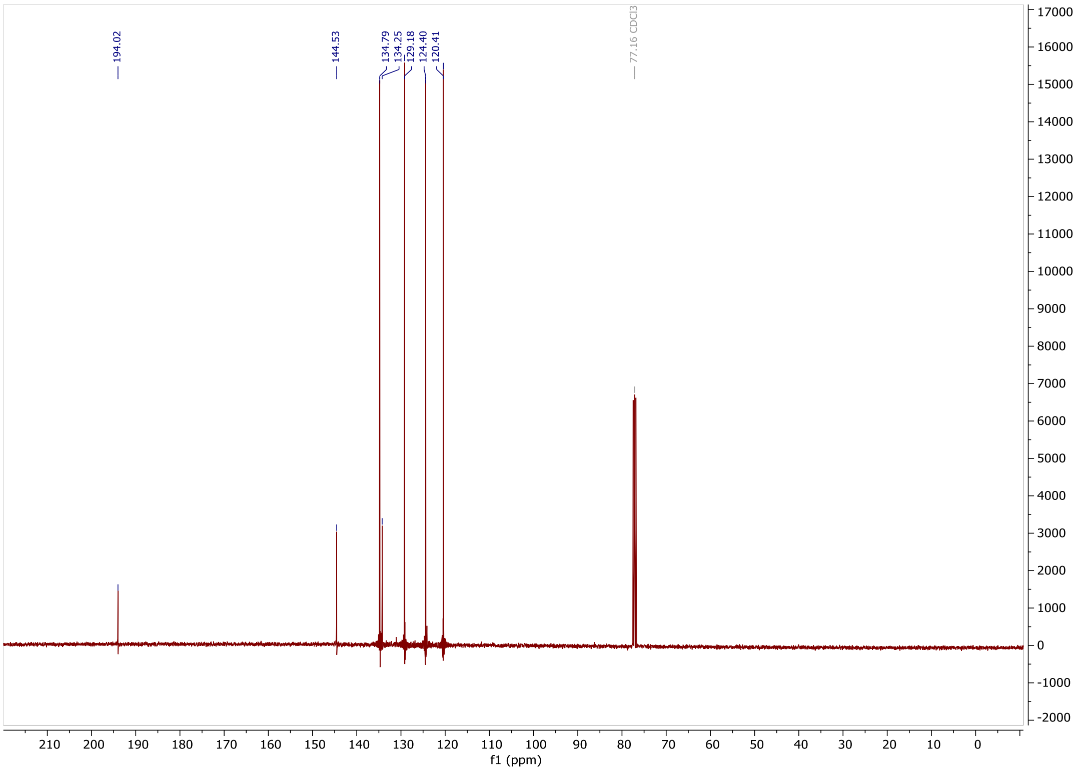

Bis(1,3-dioxoisoindolin-2-yl) phthalate 12

^1H NMR, $CDCl_3$, 400 MHz.

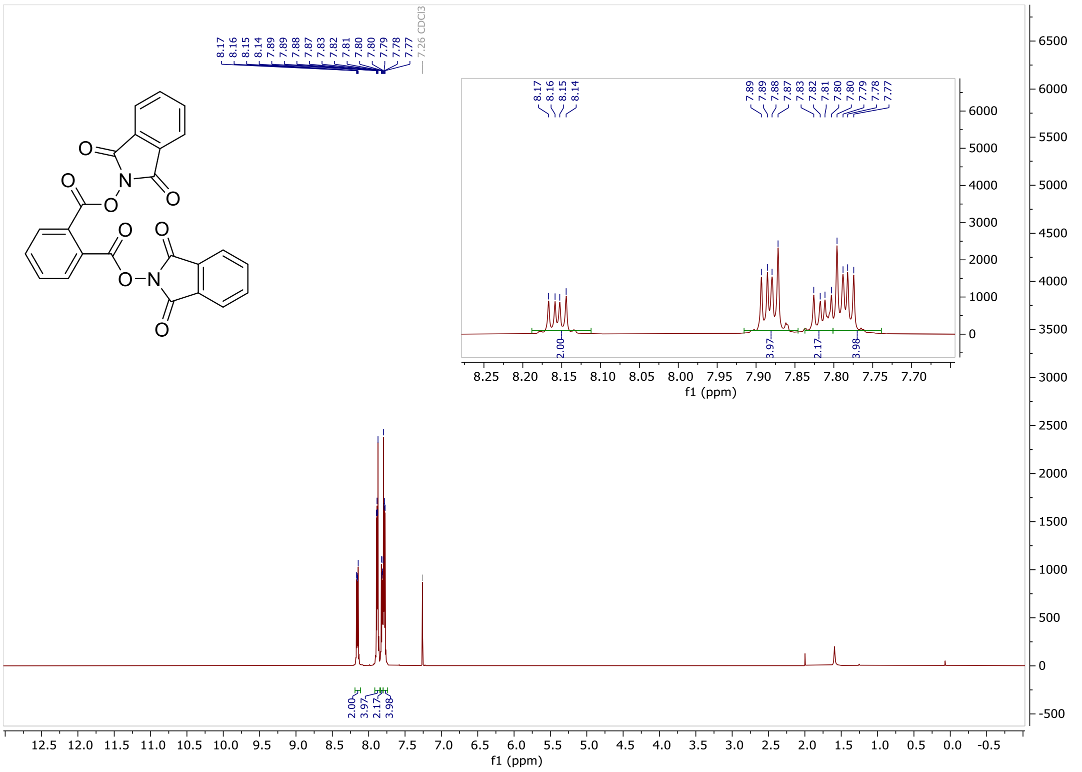

$^{13}C\{^1H\}$ NMR, $CDCl_3$, 101 MHz.

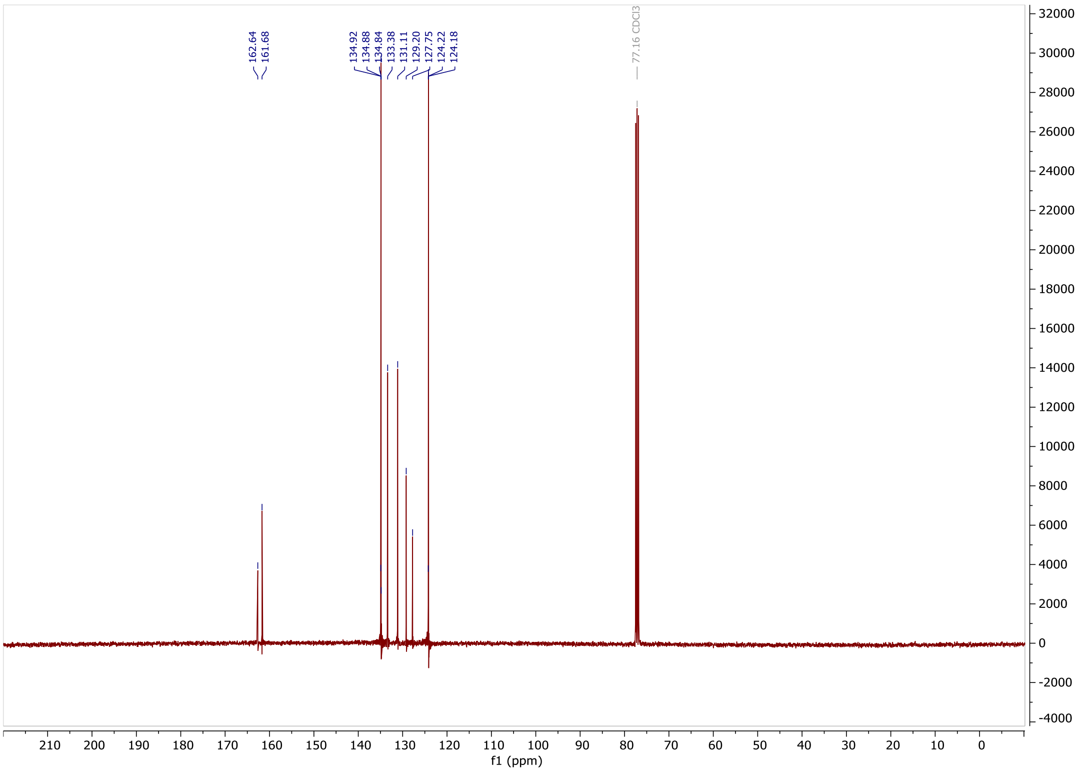

Phthalide 23

^{1}H NMR, $CDCl_3$, 400 MHz.

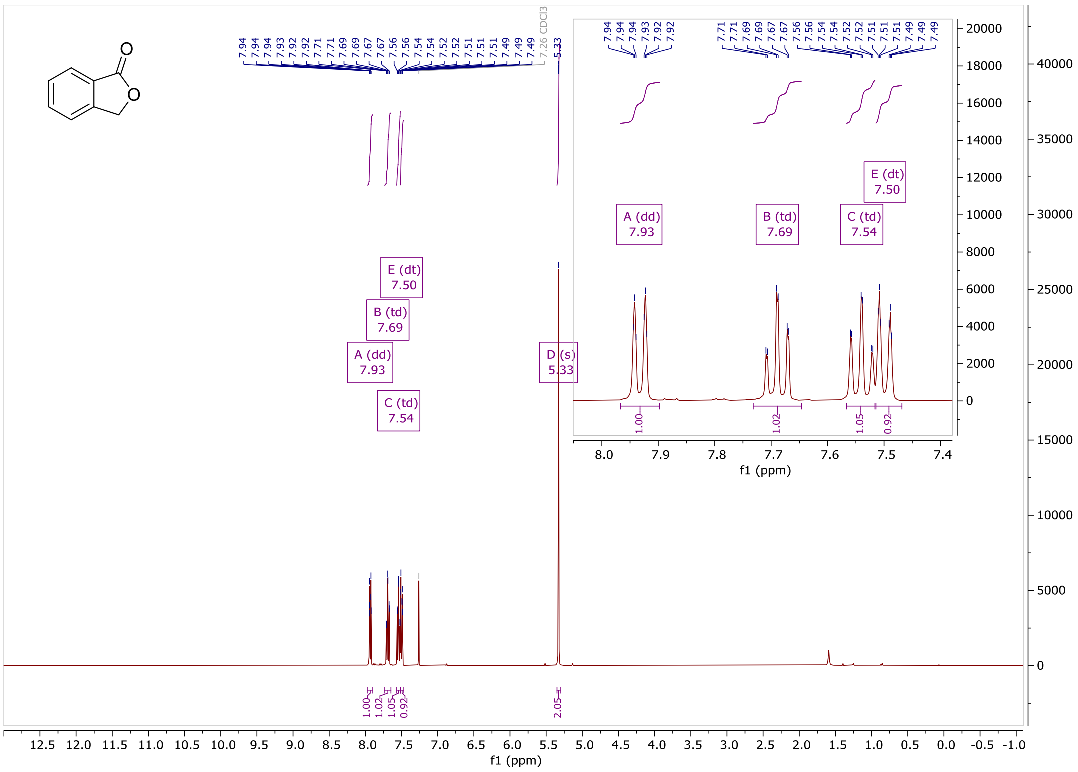

$^{13}C\{^{1}H\}$ NMR, $CDCl_3$, 101 MHz.

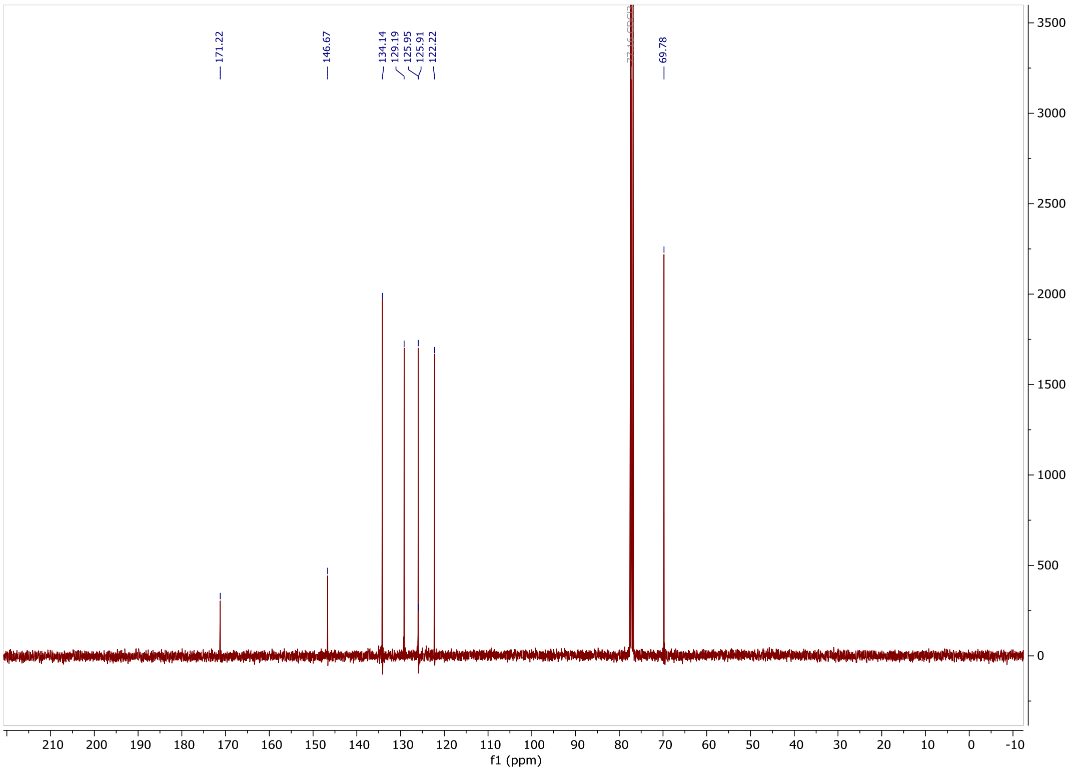

References

1 Hanson, G. R.; Gates, K. E.; Noble, C. J.; Griffin, M.; Mitchell, A.; Benson, S. XSophe-Sophe-XeprView®. A computer simulation software suite (v. 1.1.3) for the analysis of continuous wave EPR spectra. *J. Inorg. Biochem*. **2004**, *98*, 903-916.

2 Silvestre, S. M.; Salvador, J. A. R. Allylic and benzylic oxidation reactions with sodium chlorite. *Tetrahedron* **2007**, *63*, 2439–2445.

3 Okamoto, K.; Sakata, N.; Ohe, K. Copper-Catalyzed Cyanation of Aryl- and Alkenylboronic Reagents with Cyanogen Iodide. *Org. Lett.* **2015**, *17*, 4670–4673.

4 Ueda, C.; Noyama, M.; Ohmori, H.; Masui, M. Reactivity of Phthalimide-N-oxyl: A Kinetic Study. *Chem. Pharm. Bull*. **1987**, *35*, 1372–1377.

5 Zdilla, M. J.; Lee, A. Q.; Abu-Omar, M. M. Concerted Dismutation of Chlorite Ion: Water-Soluble Iron-Porphyrins As First Generation Model Complexes for Chlorite Dismutase. *Inorg. Chem*. **2009**, *48*, 2260–2268.

6 Sutton, S. C.; Cleland, W. E.; Hammer, N. I. Introducing Students to a Synthetic and Spectroscopic Study of the Free Radical Chlorine Dioxide. *J. Chem. Educ.* **2017**, *94*, 515–520.

7 Nelsen, S. F.; Blackstock, S. C.; Kim, Y. Estimation of inner shell Marcus terms for amino nitrogen compounds by molecular orbital calculations. *J. Am. Chem. Soc.* **1987**, *109*, 677−682.
